# Kinetic Pathway Control in the Synthesis of Well‐Defined Ruthenium Coordination Oligomers

**DOI:** 10.1002/smsc.202400504

**Published:** 2025-02-03

**Authors:** Tilman Schneider, Florian Seebauer, Frank Würthner, Florian Beuerle

**Affiliations:** ^1^ Institut für Organische Chemie Universität Würzburg Am Hubland 97074 Würzburg Germany; ^2^ Center for Nanosystems Chemistry (CNC) Universität Würzburg Am Hubland 97074 Würzburg Germany; ^3^ Institut für Organische Chemie Eberhard Karls Universität Tübingen Auf der Morgenstelle 18 72076 Tübingen Germany

**Keywords:** coordination oligomers, kinetic pathway control, metallosupramolecular chemistry, ruthenium bda complexes, water oxidation catalysts

## Abstract

Ruthenium complexes with 2,2′‐bipyridine‐6,6′‐dicarboxylate (bda) ligands have emerged as highly potent catalysts for water oxidation. In this context, the accumulation of active Ru centers in macrocyclic arrays or coordination oligomers and polymers has proven to be very beneficial for an enhanced stability under operating conditions and to facilitate surface adhesion in heterogeneous systems. For a better insight into structure–activity relationships though, well–defined systems with a precise control over stoichiometry and constitution are highly desired. Herein, the synthesis and characterization of a series of structurally precise and monodisperse linear coordination oligomers [(Ru(bda))_*n*_ *L*_*n−*1_ *pic*_2_] (*n* = 4 or 5, *L* = 4,4′‐bipyridine or 1,4‐bis‐(pyridine‐4‐yl)benzene derivatives, *pic* = 4‐picoline), in excellent purity and yields are reported. Based on detailed mechanistic investigations, a complex network of interconnected and competing reactions is proposed that fully explains both the high overall turnover and the kinetic pathway selection between alternative endcapping and dissociation–elongation sequences.

## Graphical abstract

Ru(bda) coordination oligomers with defined length have been prepared from bidentate linkers and pyridine endcaps. While the high yield of the reactions is driven by the thermodynamically favorable formation of strong Ru–pyridine bonds, the oligomer length is kinetically controlled by varying rates for the various dissociation, elongation, and capping steps.

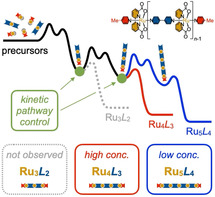

## 1 Introduction

Photo‐^[^ ^1^ ^]^ or electrochemical^[^ ^2^ ^]^ water splitting is a key process toward a sustainable energy production via the conversion of sunlight or renewable energy into solar or e‐fuels.^[^ ^3^ ^]^ Typically, the water oxidation half reaction^[^ ^4^ ^]^ is considered as the bottleneck of this process^[^ ^5^ ^]^ due to complex kinetics for the four‐electron transfer and high overpotentials.^[^ ^4^, ^5^, ^6^ ^]^ Over the years, Ru(bda) complexes^[^ ^7^ ^]^ (bda = 2,2′‐bipyridine‐6,6′‐dicarboxylate) have emerged as highly potent molecular water oxidation catalysts (WOCs).^[^ ^8^ ^]^ While mononuclear complexes are easily accessible via coordination chemistry and allow for detailed mechanistic studies in solution, they are prone to decomposition via axial ligand dissociation and any pronounced solubility might expedite device disintegration and hamper durability in real‐time applications. In particular, the integration of active Ru(bda) sites as electrode support in electrochemical setups required multinuclear compounds such as linear oligomers^[^ ^9^ ^]^ or macrocyclic assemblies.^[^ ^10^ ^]^ In recent years, we successfully applied supramolecular approaches toward diverse Ru(bda) assemblies^[8b]^ such as macrocycles,^[^ ^11^ ^]^ water‐soluble polymers,^[^ ^12^ ^]^ heterogeneous crystalline frameworks,^[^ ^13^ ^]^ or monomeric WOCs immobilized in porous cages^[^ ^14^ ^]^ to investigate WOCs in chemical, photochemical, or electrochemical setups.

Just recently, we reported a monodisperse Ru‐*4*‐mer [Ru_4_(bda)_4_ *bipy*_3_ *pic*_2_], which showed unprecedented performance and stability as active electrode support in electrochemical water oxidation.^[^ ^15^ ^]^ During synthesis though, we observed an unexpected and, to date, not understood scrambling of supposedly innocent Ru–*N* coordination bonds. For further improvement of this system, a detailed understanding of the formation mechanism and a better control on ligand scrambling during synthesis is inevitable. Generally, Ru–*N* complexes are considered kinetically inert and the exchange of weakly bound leaving groups, for example, dimethylsulfoxide (*dmso*), via a dissociative mechanism is largely considered an irreversible process.^[^ ^16^ ^]^ In combination with the strong directionality of bidentate coordination to Ru, this pronounced stability allowed the isolation of metallosupramolecular assemblies with up to 20 Ru centers.^[^ ^17^ ^]^ However, in‐depth knowledge about thermodynamics and kinetics is rare^[^ ^18^ ^]^ but would be essential for full structural control in the synthesis of precise coordination oligomers or more complex structures. Here, we provide a detailed study on the formation and product selection for a series of Ru(bda) coordination oligomers with various bis(pyridine) bridging ligands. Via detailed mechanistic studies, we revealed a striking kinetic pathway selection for the thermodynamically highly favorable oligomer formation, which ultimately decides the oligomer length by a subtle and, in some cases fully switchable, balance between termination via endcapping and elongation via ligand scrambling.

## 2 Results and Discussion

**Figure 1:**
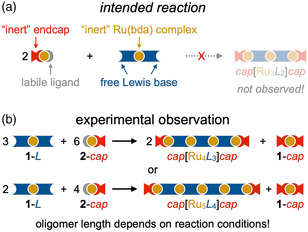
a) Expected and b) experimentally observed products in modular oligomerization reactions for Ru(bda) complexes.

Aiming for monodisperse Ru(bda)‐*n*‐mers *X*[**Ru**_***n***_ ***L***_***n*** **−1**_]*X* with precisely adjustable length, we originally envisioned a modular synthetic capping approach. For the readers’ convenience, we will use a simplified notation that puts the linear repetition unit [Ru(bda)–*L*–]_*n*−1_ in shortened form as bold in square brackets [**Ru**_***n***_ ***L***_***n*** **−1**_] and highlights the two terminal ligands X outside of this core unit. To distinguish from the equatorial bda unit, all axial ligands are typed in italics. As the length‐determining motif, we planned to use well‐defined precursors *L*[**Ru**_***n***_ ***L***_***n*** **−1**_]*L* in which a specific number of Ru(bda) complexes are cross‐linked and saturated with linear bidentate linkers *L* at the axial positions. As the shortest derivatives of such a series, symmetrical mononuclear complexes *L*[**Ru**]*L* (**1**−*L*) are easily accessible by reaction of **1**‐*dmso* with a twentyfold excess of bidentate linkers *L* in degassed MeOH at 70 °C for 1 h (see Supporting Information for synthetic procedures and analytical data). As capping reagent, unsymmetrical mononuclear complex *pic*[**Ru**]*dmso* (**2**‐*pic*)^[^ ^19^ ^]^ was employed, in which the inert *pic* serves as endcap while the very labile leaving group *dmso* should be readily replaced by the free 4‐pyridyl (*pyr*) sites of the core units. As we assumed all Ru–*pyr* bonds to be strong and inert under reaction conditions, only endcapping should take place with a defined oligomer *pic*[**Ru**_**3**_ ***L***_**2**_]*pic* as the only feasible product (**Figure** **1**a).

**Scheme 1:**
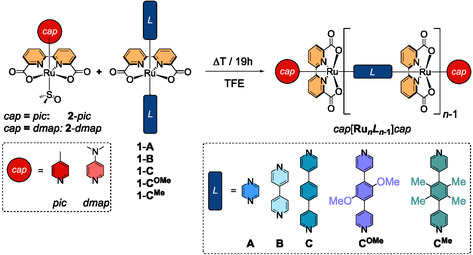
Synthesis of well‐defined coordination oligomers based on Ru(bda) nodes and bidentate linkers.

As we recently reported though,^[^ ^15^ ^]^ a longer oligomer *pic*[**Ru**_**4**_ ***B***_**3**_]*pic* (***B*** = 4,4′‐bipyridine) was the only isolated product instead of the envisioned *pic*[**Ru**_**3**_ ***B***_**2**_]*pic* for the reaction of **1**‐**B** with **2**‐*pic*. This unexpected outcome can only be explained if scrambling of the *apparently not–so–inert* Ru–*B* coordinative bonds is considered. To get a more detailed mechanistic insight, we further investigated this reaction and expanded the scope to shorter and longer linkers pyrazine (*pyz*, **A**) and 1,4‐di(pyridin‐4‐yl)benzene (**C**),^[^ ^20^ ^]^ respectively. To probe for electronic and steric effects, derivatives of **1**‐**C** containing 4,4′‐(2,5‐dimethoxy‐1,2‐phenylene)dipyridine (**C** ^**OMe**^)^[^ ^21^ ^]^ and 4,4′‐(2,3,5,6‐tetra‐methyl‐1,2‐phenylene)dipyridine (**C** ^**Me**^)^[^ ^22^ ^]^ have also been synthesized (see Supporting Information for synthetic procedures and analytical data). For all precursors **1**‐*L*, a detailed screening of reaction conditions for oligomer formation was performed (see Supporting Information for more details). While reaction at 60 °C only led to incomplete oligomer formation (Figure S48, Supporting Information), full conversion was obtained in refluxing 2,2,2‐trifluoroethanol (TFE) at 78 °C and the reaction outcome, in most cases, did not significantly change for reactions in a microwave reactor at 100 °C (Table S6, Supporting Information). Any specific temperature effect in certain reactions will be addressed later in the mechanistic discussion. Regarding the stoichiometry of the two precursors, we realized that any larger deviation from the optimal 1:1 ratio between free *pyr* sites in **1**‐*L* and the *dmso* leaving groups in **2**‐*pic* severely hampered the isolation of defined oligomers. Instead, only soluble mixtures consisting of short fragments with either one or two Ru(bda) units were obtained in case of larger excess of any of the two reactants (Figure S49, Supporting Information). Apparently, all available coordination sites must have a chance to react for efficient progress of the reaction. If not, the reaction will rather stop at ill‐defined mixtures of early intermediates. In a typical procedure (**Scheme** **1**), a mixture of precursors **1**‐*L* and **2**‐*pic* in 1:2.1 molar ratio in TFE was heated to 80 °C for about 19 h. According to NMR monitoring experiments, this time period proved sufficient for full consumption of the starting materials (see Figure S39 and S40, Supporting Information). After evaporation of the solvent, the obtained solids were purified by sequential washing with MeOH, CH_2_Cl_2_, Et_2_O, and *n*‐pentane. Thereby, unreacted precursors, shorter fragments, and other soluble byproducts were dissolved, while oligomers remained as solid materials.

**Figure 2:**
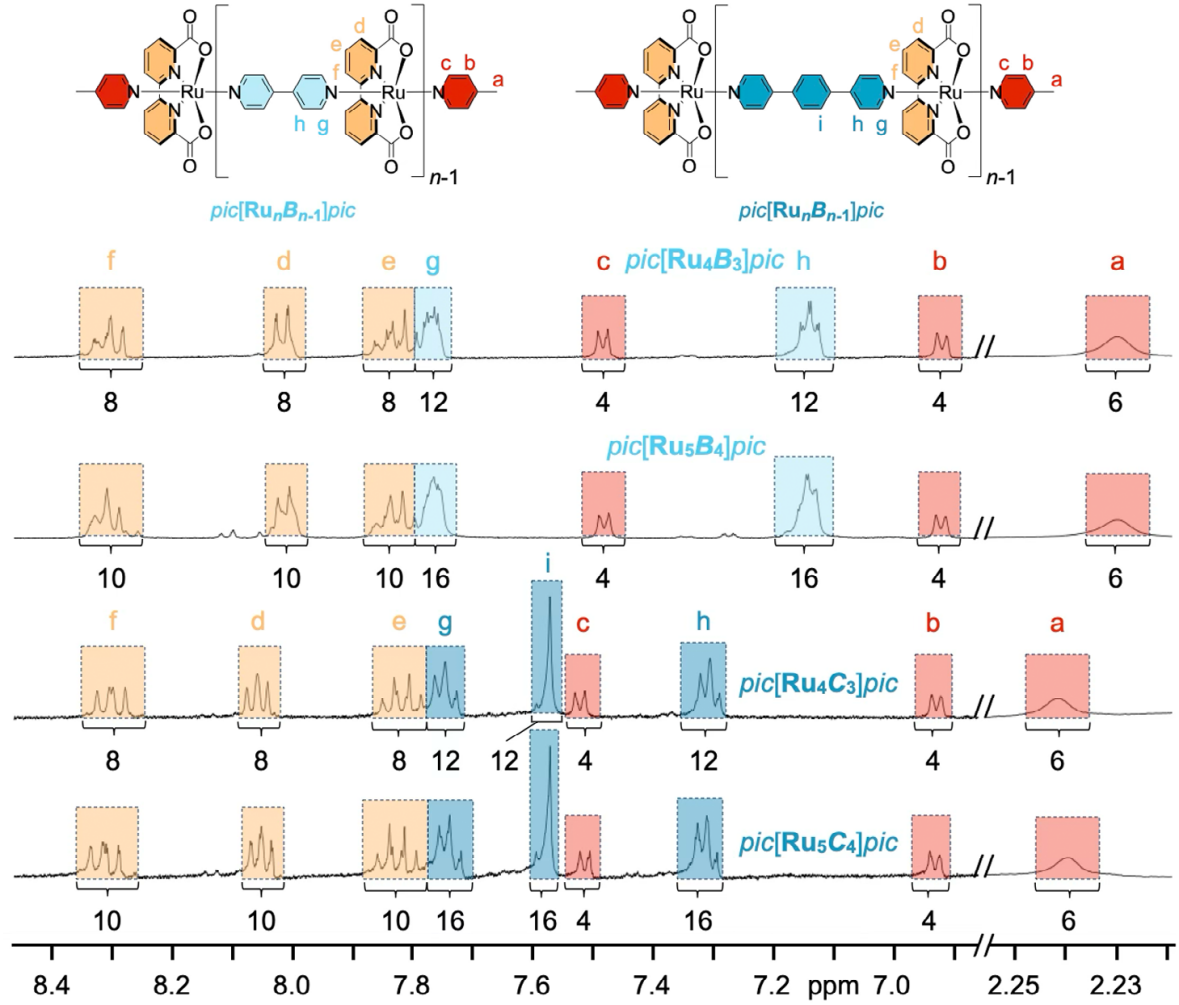
Size determination of Ru(bda) oligomers based on ligands **B** and **C** via end group analysis using ^1^H NMR spectroscopy (400 MHz, CD_2_Cl_2_/TFE‐*d*_3_ 9:1, ascorbic acid). NMR signals for the different components *pic* (red), bda (yellow), **B** (light blue) and **C** (dark blue) are highlighted in matching colors.

Typically, mixtures of smaller oligomers were obtained via this procedure, but the exact composition seemed very sensitive to even subtle changes in the experimental conditions, for example, concentration or temperature. Exemplary NMR spectra for such oligomer mixtures are displayed in Figure S42–S46, Supporting Information. After meticulous optimization though, at least one of the higher homologs *pic*[**Ru**_**4**_ ***L***_**3**_]*pic* or *pic*[**Ru**_**5**_ ***L***_**4**_]*pic* could be isolated as a pure compound for all linkers *L* except pyrazine **A** (Table S3, Supporting Information). Identification and length determination of all pure oligomers were carried out by ^1^H NMR end group analysis. As exemplarily shown in **Figure** **2** for the four oligomers obtained from linkers **B** and **C**, integer ratios between integrals for protons from either *pic* (red), bda (yellow), and **B** (light blue) or **C** (dark blue) moieties clearly indicated the integrity, defined length and exact chemical constitution for each of the isolated products (see Figure S32–S34, Supporting Information for end group analysis for the other isolated oligomers).

**Figure 3:**
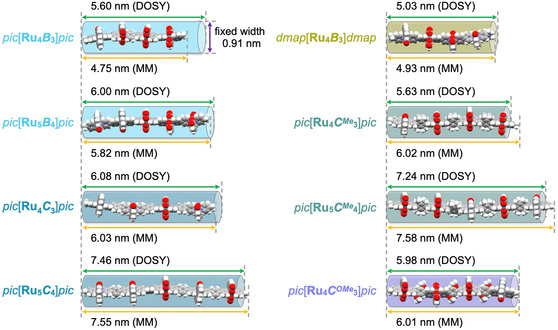
Graphical comparison of the molecular size for all isolated oligomers *cap*[**Ru**_***n***_ ***L***_***n*** **−1**_]*cap* obtained from either ^1^H DOSY NMR measurement (colored semitransparent cylinders) or molecular modeling (space‐filling models from UFF force‐field calculations, *Materials Studio*).

As a complementary technique, we used DOSY NMR for oligomer length estimation. Due to the prolate shape of the oligomers, the spherical particle approach of the Stokes–Einstein equation is not applicable.^[^ ^23^ ^]^ Instead, the aspect ratio and molecular dimensions of the oligomers were estimated with a cylindrical model introduced by Tirado et al.^[^ ^24^ ^]^ (see Supporting Information for more details). Based on molecular modeling and literature data,^[^ ^25^ ^]^ the width of the oligomer chain was fixed at 0.91 nm as the assumed expansion of one bda unit. Fitting of the experimental decay curves provided much better agreement with a monoexponential function compared to a log‐normal model (see Figure S60, Supporting Information), thus indicating the formation of well‐defined monodisperse oligomers. Based on diffusion constants in the range of 3.24–4.08 × 10^−10^ m^2^ s^−1^, oligomer lengths between 5.03 and 7.46 nm were calculated (Table S10, Supporting Information). These DOSY data are in excellent agreement with geometry‐optimized molecular models (Universal force‐field (UFF), *forcite* task in *Materials Studio*, see Supporting Information for details)^[^ ^26^ ^]^ with a mean deviation below 0.1 nm (**Figure** **3**). The somewhat larger deviation for the smallest *pic*[**Ru**_**4**_ ***A***_**3**_]*pic* with an aspect ratio of around 5 is presumably caused by geometrical limitations of the applied cylindrical model, which is only valid for aspect ratios >2. In total, both ^1^H and DOSY NMR provided strong evidence for the monodisperse nature of all isolated oligomers.

Unfortunately, the limited stability in solution for the coordinative [Ru]–*pyr* bonds led to pronounced fragmentation in ESI MS. Only fragments with up to two Ru(bda) units were detected after ionization and no information about the oligomer size could be obtained.^[^ ^15^ ^]^

Intriguingly, under the optimized conditions, these compounds were not isolated as minor side products but with typically more than 90% yield (**Table** **1**). Since the composition of the products does not reflect the stoichiometry of the starting materials, we searched for any other Ru‐containing products and identified *pic*[**Ru**]*pic* (**1**‐*pic*) as the second major product in all cases (Figure S35, Supporting Information). Apparently, oligomer formation is described by either reaction Equation (1) or (2) (see also Figure 1b) with the two products both being typically isolated in very high yields above 90% (see Table S1, Supporting Information for an exemplary calculation for *pic*[***Ru***_***5***_ ***B***_***4***_]*pic*).

(1) $$3\;L[Ru]L+6\;pic[Ru]dmso\to 2\;pic[Ru_{4}L_{3}]pic+pic[Ru]pic$$

(2) $$2\;L[Ru]L+4\;pic[Ru]dmso\to pic[Ru_{5}L_{4}]pic+pic[Ru]pic$$

**Table 1:** Main products and isolated yields for oligomerization reactions with varying linkers.

Entry	Linker	Endcap	T	**c(Ru)**_**tot**_ b)	Product (isolated yield)	
	*L*	*cap*	[°C]	[mM]	*cap*[**Ru**_**4**_ ***L***_**3**_]*cap*	*cap*[**Ru**_**5**_ ***L***_**4**_]*cap*
1	**A** (*pyz*)	*pic*			*No defined oligomers isolated*	
2	**B**	*pic*	78	11.9		*pic*[**Ru**_**5**_ ***B***_**4**_]*pic* (96%)
3	**B**	*pic*	78	23.8	*pic*[**Ru**_**4**_ ***B***_**3**_]*pic* (94%)	
4	**B**	*dmap*	100a)	3.9–12.4	*dmap*[**Ru**_**4**_ ***B***_**3**_]*dmap* (81%)	
5	**C**	*pic*	78	3.9		*pic*[**Ru**_**5**_ ***C***_**4**_]*pic* (93%)
6	**C**	*pic*	78	7.8	*pic*[**Ru**_**4**_ ***C***_**3**_]*pic* (74%)	
7	**C** ^**OMe**^	*pic*	78	3.9–7.8	*pic*[**Ru**_**4**_ ***C*** ^***OMe***^_**3**_]*pic* (91%)	
8	**C** ^**Me**^	*pic*	78	3.9–7.8		*pic*[**Ru**_**5**_ ***C*** ^***Me***^_**4**_]*pic* (74%)
9	**C** ^**Me**^	*pic*	100a)	3.9–7.8	*pic*[**Ru**_**4**_ ***C*** ^***Me***^_**3**_]*pic* (97%)	

a) Reaction carried out in a microwave reactor;

b) Total concentration of Ru(bda) complexes (sum of both starting materials).

To get further insight into the stability of such oligomers and potential scrambling pathways, we performed specific ligand exchange experiments (Table S2, Supporting Information). For symmetrical mononuclear complexes **1**‐*X*, (*X* = **B**, **C**, *pic*), only neglectable axial ligand exchange occurred even at a high excess of more than 200 equivalents of a competing ligand. For multinuclear oligomers *pic*[**Ru**_**4**_ ***C***_**3**_]*pic* and *pic*[**Ru**_**5**_ ***C***_**4**_]*pic* however, axial ligand exchange is apparently possible since an excess of either free **B** or **1**‐**B** led to full decomposition into complex mixtures of soluble fragments. As another proof of ligand scrambling, NMR experiments with oligomers dissolved in pyridine‐*d*_5_ as a coordinating solvent were carried out. Both heating of *pic*[**Ru**_**5**_ ***C***_**4**_]*pic* in pristine pyridine‐*d*_5_ (Figure S37, Supporting Information) as well as *pic*[**Ru**_**4**_ ***B***_**3**_]*pic* in pyridine‐*d*_5_/TFE‐*d*_3_ (Figure S38, Supporting Information) resulted in the emergence of signals of the respective free linker **B** or **C** after a few hours.

**Figure 4:**
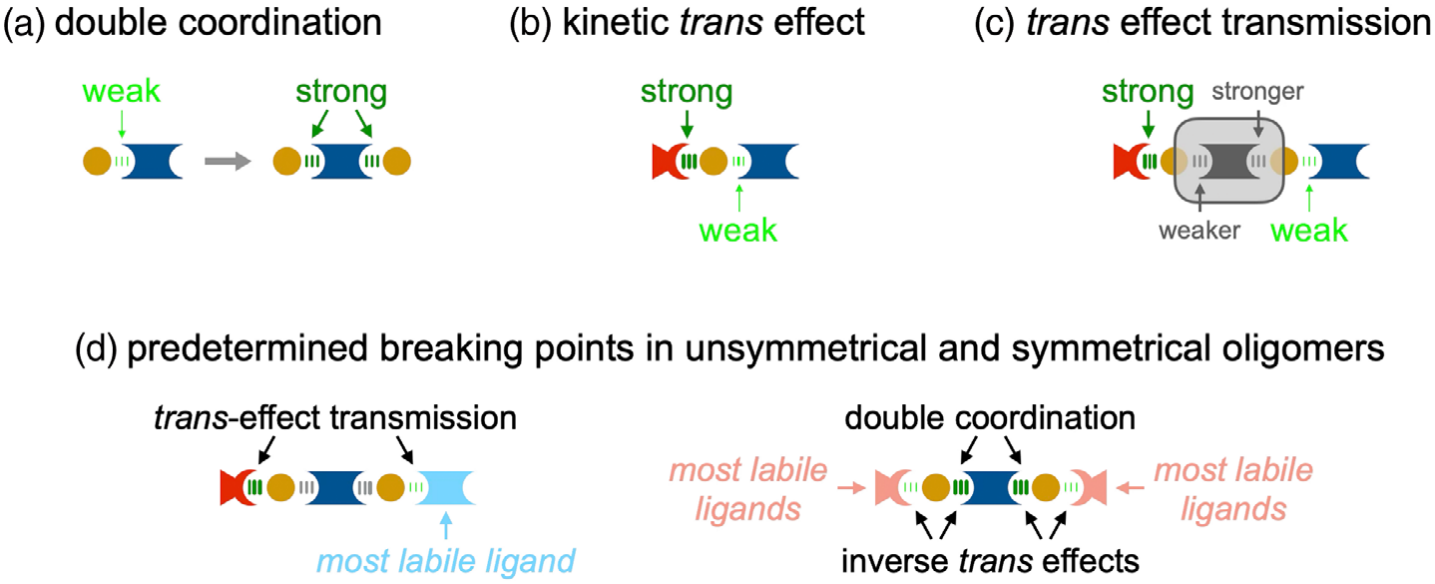
Major kinetic effects that influence the stability of axially coordinated ligands in Ru(bda) complexes: a) [Ru]–*L* bond stabilization by double coordination, b) kinetic *trans* effect in unsymmetrical complexes, and c) *trans* effect transmission in unsymmetrical oligomers; d) based on these effects, the most labile ligands are identified in unsymmetrical and symmetrical oligomers.

Based on an exhaustive literature survey, we attribute any gradual lability of axial ligands in oligomeric assemblies to three major effects (**Figure** **4**): As a first factor, the initial coordination event in communicating bidentate ligands *L* activates the second coordination site (Figure 4a). According to literature reports for the *pyz* ligand, a lower acidity, and thus, higher basicity, is observed for the second *N* atom upon complexation of the first *N* atom with Ru(II).^[^ ^27^ ^]^ The coordination to the relatively electron‐rich metal center with significant metal‐to‐ligand π‐back donation enhances the electron density at the ligand and, consequently, increases the σ‐donor strength for a second coordination. Here, we assume a similar effect for conjugated bidentate linkers *L*, thus leading to a cooperative increase in Ru–*pyr* bond stability for bis‐coordinated linkers [Ru]–*L*–[Ru] compared to mono‐coordinated *L* units [Ru]–*L* with one free pyridine site. As the second important factor, the well‐established *kinetic trans effect* (KTE)^[^ ^28^ ^]^ induces an unequal bond strength distribution in *X*–[Ru]–*Y* fragments with unsymmetric axial coordination (Figure 4b). Typically, a strong KTE is induced from a ligand X that is a strong σ‐donor or π–acceptor (or both).^[^ ^29^ ^]^ Thereby, the opposing Ru–*Y* bond to the less nucleophilic ligand Y is significantly weakened.^[^ ^30^ ^]^ For the systems applied in this work, we assume that the *pic* ligand weakens the coordination of *dmso* in **2**‐*pic* as well as monocoordinated *L* in *pic*[**Ru**]*L*. Considering the mutual stabilization in doubly coordinated *L* though, the stability is reversed and terminal *pic* becomes the more labile position compared to internal [Ru]–*L*–[Ru] sites in multinuclear assemblies such as *pic*[**Ru**_**2**_ ***L***]*pic*. As the third effect, *trans* effect transmission (TET) allows for a propagation of the electronic effects from one terminal ligand X to another remote ligand Y, which are connected via a conjugated bridging ligand *L* in *trans*‐connected binuclear complexes *X*–[Ru]–*L*–[Ru]–*Y* (Figure 4c).^[^ ^31^ ^]^ The most famous example for such an electronic communication between the Ru centers in binuclear complexes is the Creutz–Taube complex^[^ ^32^ ^]^ as a mixed‐valence compound. Based on these literature precedents, we propose similar effects for the oligomer series in this work dependent on the length and conjugation along *L*.

For all Ru(bda) oligomers covered in this work, we expect the highest displacement in bond strength distribution at the terminal Ru centers (Figure 4d). In unsymmetrical oligomers *pic*[**Ru**_***n***_ ***L***_***n*** **−1**_]*L*, which are important intermediates during oligomer formation, we propose an alternating pattern of weaker and stronger bonds along the oligomer backbone and a transmission of the KTE of the strong Ru–*pic* bond to the labile terminal Ru–*L* bond (Figure 4d) as the predetermined breaking point in these systems. If the length of the oligomer increases or the conjugation within *L* decreases, this effect becomes weaker due to the fading interaction between the two terminal Ru centers. In symmetrical oligomers, however, there is no remote *trans* effect but the bonding situation at the terminal groups depend on the relative strength of the external and internal ligands. Assuming a strong double‐coordination effect, the KTE might be inversed, thus making the *“capping”* *pic* the most labile ligand in the system.

Considering all experimental observations, we have drawn two main conclusions: (i) According to the *principle of maximum site occupancy*, there is a strong thermodynamic driving force to replace all weak Ru–*dmso* bonds with more stable Ru–*pyr* bonds and the overall reaction proceeds until all Ru sites are saturated with either *L* or *pic* at both axial positions. (ii) At least some Ru–*L* and Ru–*pic* bonds are labile enough under reaction conditions to temporarily dissociate into an equilibrium between saturated [Ru]–*X* and transient [Ru]–□ with one free coordination site at Ru (symbolized by the empty square). As long as there are enough free *pyr* sites available, this process may lead to scrambling of the axial ligands, thus facilitating oligomer elongation. While the *progress of the reaction* is driven by *thermodynamics* and the favorable formation of strong Ru–*pyr* bonds, the *outcome of the reaction* and *oligomer length* is controlled by *kinetics* and the rates of the various dissociation, elongation, and capping steps.

**Figure 5:**
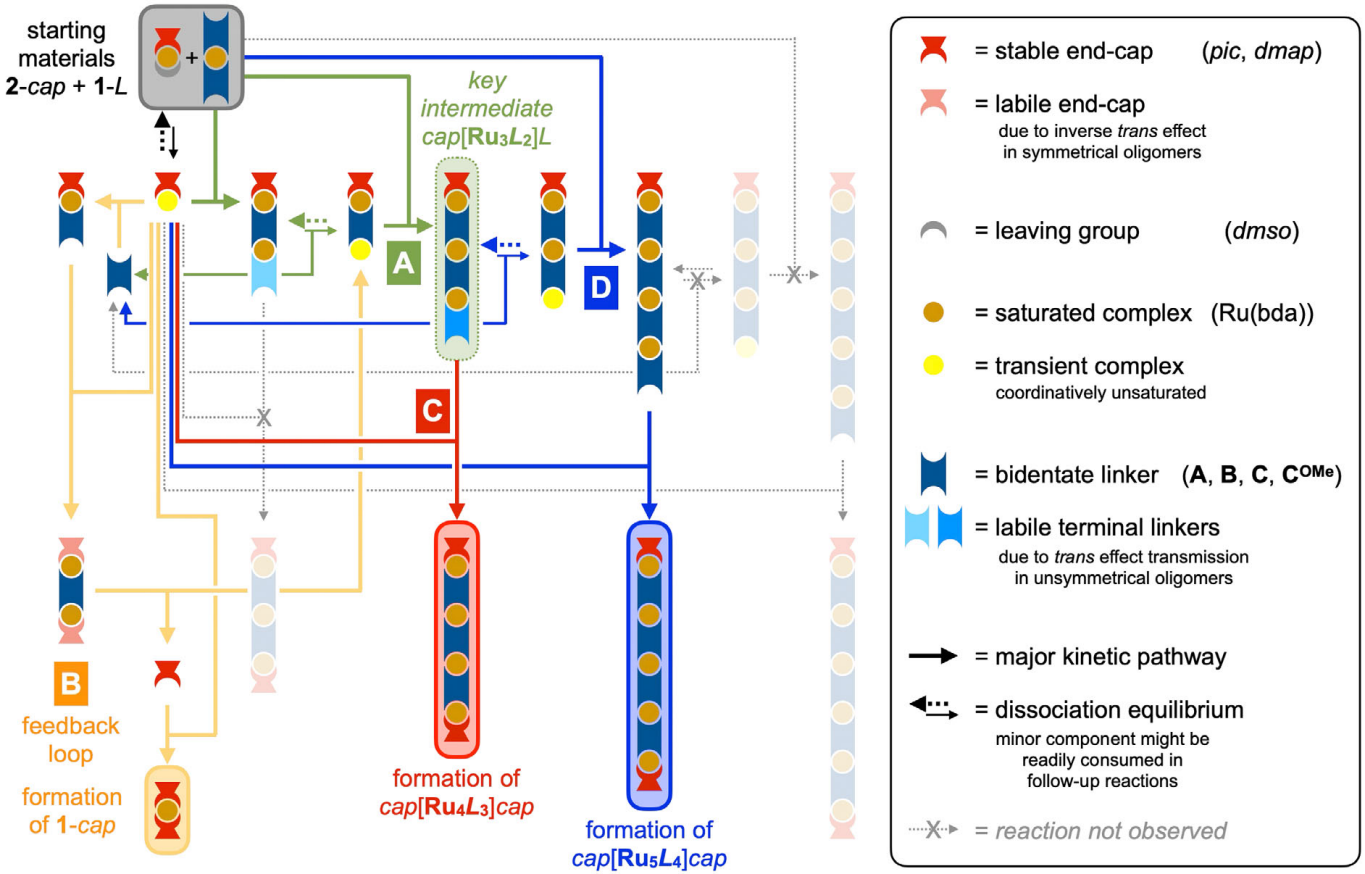
Complex kinetic reaction network for the formation of well‐defined Ru(bda) coordination oligomers consisting of the initial formation of key intermediate *cap*[**Ru**_**3**_ ***L***_**2**_]*L* (A, green), a feedback loop for the formation of byproduct **1**‐*cap* (B, yellow) and two competing pathways for the capping reaction toward shorter oligomer *cap*[**Ru**_**4**_ ***L***_**3**_]*cap* (C, red) and the dissociation–elongation sequence toward longer oligomer *cap*[**Ru**_**5**_ ***L***_**4**_]*cap* (D, blue).

Based on these considerations, we propose a comprehensive mechanism for the oligomer formation, which consists of four distinct steps (**Figure** **5**). In the following, we provide a detailed and stepwise discussion of this proposal and provide analytical evidence that this picture is fully consistent with any experimental observations. As the initial step, we postulate the formation of key intermediate *pic*[**Ru**_**3**_ ***L***_**2**_]*L* from precursors **1**‐*L* and **2**‐*pic* via pathway A (green in Figure 5). Facile dissociation of *dmso* provides activated endcap *pic*[**Ru**]–□ that readily reacts with one core unit **1**‐*L* toward *pic*[**Ru**_**2**_ ***L***]*L*. At this point, the pronounced TET makes the terminal Ru–*L* bond so labile that this intermediate cannot be captured by a second end group **2**‐*pic* but rather dissociates into free ligand *L* and transient *pic*[**Ru**_**2**_ ***L***]–□, which is coordinatively unsaturated and highly reactive toward any nucleophiles. While trivial back reaction with *L* establishes an equilibrium, **1**‐*L* is available as competing nucleophile especially at the beginning of the reaction. Thus, elongation into key intermediate *pic*[**Ru**_**3**_ ***L***_**2**_]*L* dominates over the slower capping reaction toward elusive *pic*[**Ru**_**3**_ ***L***_**2**_]*pic*, which has never been isolated or even identified in any synthetic attempts during this study. As a matter of fact, the fast initial coordination between **1**‐*L* and **2**‐*pic* followed by dissociation and elongation with another molecule of **1**‐*L* is also in line with the faster decline of signals for precursor **1**‐*L* compared to endcap **2**‐*pic* in ^1^H NMR reaction monitoring measurements (Figure S40c, Supporting Information).

In a parallel pathway B (yellow in Figure 5), any free linker *L* that has been released in pathway A readily reacts with two equivalents of **2**, thus forming the binuclear complex *pic*[**Ru**_**2**_ ***L***_**1**_]*pic*. However, the inverse *trans* effect postulated before significantly weakens the [Ru]–*pic* bonds of the endcaps,^[^ ^33^ ^]^ which makes these bonds more labile than the mutually reinforcing [Ru]–*L*–[Ru] bonds. Therefore, rapid dissociation of one *pic* ligand generates the transient *pic*[**Ru**_**2**_ ***L***]–□, which is fed back into the oligomer formation pathway upon coordination of **1**‐*L*, while the released *pic* combines with excess **2**‐*pic* as the most abundant Lewis acid into stable byproduct **1**‐*pic*. Thereby, the instant consumption of the released linker *L* activates a positive feedback loop and two molecules of key intermediate *pic*[**Ru**_**3**_ ***L***_**2**_]*L* are formed from each initial condensation between **1**‐*L* and **2**‐*pic* according to reaction Equation (3).

(3) $$3\;L[Ru]L+3\;pic[Ru]dmso\to 2\;pic[Ru_{3}L_{2}]L+pic[Ru]pic$$

Via this nonlinear process, the accelerated consumption of **1**‐*L* further limits the availability of the initial condensation product *pic*[**Ru**_**2**_ ***L***]*L* for any capping reaction toward the elusive *pic*[**Ru**_**3**_ ***L***_**2**_]*pic*. As a control to probe the postulated lability of *pic*[**Ru**_**2**_ ***L***]*pic*, we explicitly targeted the formation of *pic*[**Ru**_**2**_ ***B***]*pic* via the reaction of **B** and **2**‐*pic* in 1:2.1 molar ratio in TFE at 80 °C. Intriguingly, only *pic*[**Ru**_**4**_ ***B***_**3**_]*pic* and **1**‐*pic* were isolated, albeit with a reduced yield of around 50% for the oligomer. Hereby, it became obvious that *pic*[**Ru**_**2**_ ***L***]*pic* is indeed elusive under conditions typically applied for oligomer synthesis. On the contrary, the inertness of **1**‐*pic* and the necessity of weak *dmso* ligands to initiate the reaction was demonstrated by an attempted reaction of **1**‐**B** and **1**‐*pic* in 1:2.1 molar ratio (Figure S41, Supporting Information). Here, no reaction was observed after 20 h at room temperature, while only minimal conversion occurred after heating at 80 °C for an additional 20 h. Apparently, the symmetrical mononuclear coordination lacking any KTE apparently hampers any facile dissociation. In contrast, a mixture of **1**‐**B** and **2**‐*pic* smoothly starts to react even at room temperature, while pure samples of either starting material remain almost unchanged (Figure S40, Supporting Information). Obviously, the combination of significant amounts of reactants containing both free *pyr* sites and labile leaving groups, for example, *dmso*, are necessary for efficient ligand scrambling.

As evident from Equation (3), the initial condensation in combination with the feedback loop B facilitates the nonlinear formation of *pic*[**Ru**_**3**_ ***L***_**2**_]*L* as a metastable intermediate. As this structure possesses one free *pyr* site and there is still excess **2**‐*pic* available, the reaction is still not finished but further replacement of weakly bound *dmso* with more stable *pyr* ligands is thermodynamically favorable. At this point however, two competing pathways are feasible, and this differentiation ultimately decides the outcome of any reaction. Via pathway C (red in Figure 4), the remaining second equivalent of capping reagent **2**‐*pic* coordinates any free *pyr* sites to form *pic*[**Ru**_**4**_ ***L***_**3**_]*pic* as one of the two isolated products in this series of reactions. Following alternative pathway D (blue in Figure 4) however, the KTE of the *pic* endcap is transferred even further along three Ru centers to labilize the opposing terminal ligand *L*, thus facilitating dissociation and further elongation. If the rate constants for these two pathways happen to be in the same order of magnitude, the way this key intermediate is consumed becomes crucially dependent on reaction parameters such as concentration or availability of reactants. In particular, it has to be noted that both the formation of this intermediate via pathway A and the second elongation via pathway D compete for **2**‐*pic* as a common feedstock, hence the rate of consumption and time‐dependent availability of this starting material is crucial for the kinetic pathway selection.

Two factors are decisive to make the formation of *pic*[**Ru**_**5**_ ***L***_**4**_]*pic* via pathway D the dominant process: (i) TET over three Ru centers has to be so strong that the more remote terminal *L* is labile enough to facilitate dissociation into *pic*[**Ru**_**3**_ ***L***_**2**_]–□. (ii) The rate of formation for key intermediate *pic*[**Ru**_**3**_ ***L***_**2**_]*L* has to be balanced in a way that it is, on the one hand, fast enough to accumulate substantial amounts of this intermediate but, on the other hand, slow enough so that **1**‐*L* is still available over time for the second elongation step. To probe the effect of gradual modulation of the TET, the length and conjugation of *L* can be varied from one (**A**) to two (**B**) and three (**C**) aromatic rings. For the longest linker **C**, the significantly weakened TET requires very dilute conditions (entry 5 in Table 1, total concentration of all Ru(bda) precursors c(Ru)_tot_ = 3.9 mM) to make the dissociation–elongation sequence toward *pic*[**Ru**_**5**_ ***C***_**4**_]*pic* via pathway D the dominant process. For the bipyridine linker **B**, the stronger TET accelerates dissociation and formation of *pic*[**Ru**_**5**_ ***B***_**4**_]*pic*, which is now still faster compared to capping even at higher concentrations (entry 2 in Table 1, c(Ru)_tot_ = 11.9 mM). For the shortest **A**, the very strong communication between adjacent Ru centers via one single heteroaromatic ring, in combination with the significantly reduced nucleophilicity of more electron‐poor *pyz* compared to the *pyr* sites in **B** and **C**, makes any terminal *pyz* ligand so labile and less reactive that no defined monodisperse oligomers could be isolated even after a systematic screening of reaction conditions (entry 1 in Table 1 and Table S3, Supporting Information). Instead, only mixtures of oligomers were isolated and an average length of around four Ru centers was roughly estimated by ^1^H NMR integration.

**Figure 6:**
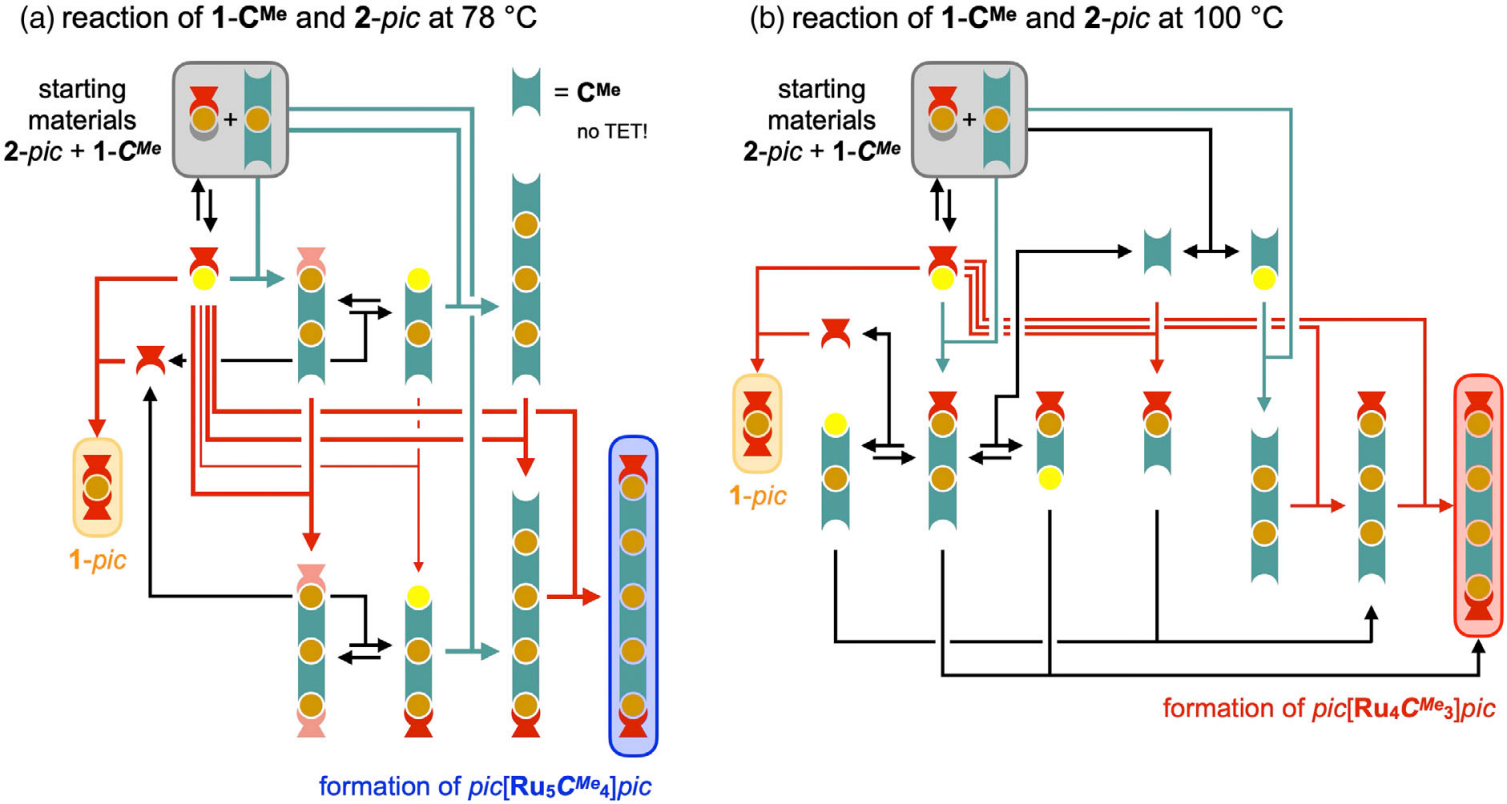
Proposed mechanism for the reaction of **1**‐**C** ^**Me**^, which has no *trans* effect transmission due to hindered conjugation, and **2**‐*pic* at a) 78 °C in refluxing TFE or at b) 100 °C in a microwave reactor.

Based on these observations, it was highly tempting to explicitly target the formation of the Ru–*3*–mers or any higher oligomers for **A** and **B** by further extending the concentration range. However, limited solubility of the precursors prevented the clean formation of *pic*[**Ru**_**3**_ ***B***_**2**_]*pic* at very high concentrations (Table S4, Supporting Information). At very high dilution however, the significant drop in overall reactivity impeded already the formation of the first intermediates, let alone the even slower synthesis of higher oligomers. As the ultimate proof for the key role of the TET in the formation of these linear coordination oligomers, we designed linker **C** ^Me^ in such a way that the exhaustive methylation of the central phenylene unit induces an orthogonal conformation of the three (hetero)aromatic rings, which basically shuts down any electronic communication between the opposite *pyr* sites (Figure S79, Supporting Information). Therefore, we initially assumed that there should be no TET or any mutual stabilization upon twofold coordination for this ligand. On the other hand, the *pyr* groups still experience a significant +I‐effect from the fourfold‐methylated central ring, which is not dependent on coordination, and may therefore permanently increase the σ–donor strength for both coordination sites via a pronounced structural *trans* effect (STE).^[^ ^30^, ^34^ ^]^ Indeed, we observed a rather different behavior of linker **C** ^**Me**^ compared to all previously discussed systems. Unlike **B** and **C**, the oligomer length for reactions between **1**‐**C** ^**Me**^ and **2**‐*pic* is not dependent on c(Ru)_tot_ but rather the reaction temperature. While standard conditions at 80 °C afforded longer *pic*[**Ru**_**5**_ ***C*** ^***Me***^_**4**_]*pic* irrespective of concentration (entry 8 in Table 1, c(Ru)_tot_ = 3.8–7.8 mM), reaction in a microwave reactor at 100 °C led to the isolation of shorter *pic*[**Ru**_**4**_ ***C*** ^***Me***^_**3**_]*pic* in excellent yield of 94% (entry 9 in Table 1, c(Ru)_tot_ = 3.8–7.8 mM). Presumably, the strong linker‐based STE reverses the electron distribution at the terminal Ru centers, which makes the [Ru]–*pic* bonds the most labile connections (**Figure** **6**a). Consequently, dissociation of the *pic* endcap is the most plausible step after initial formation of *pic*[**Ru**_**2**_ ***C*** ^***Me***^]*C* ^*Me*^. Now, the released *pic* directly reacts with **2**‐*pic* to form byproduct **1**‐*pic*. Via the transient *C* ^*Me*^[**Ru**_**2**_ ***C*** ^***Me***^]–□, fast elongation toward *C* ^*Me*^[**Ru**_**3**_ ***C*** ^***Me***^_**2**_]*C* ^*Me*^ occurs upon addition of **1**‐**C** ^**Me**^. At 80 °C, this trinuclear intermediate is stable enough, so that capping of the two free *pyr* sites with two equivalents of **2**‐*pic*, either instantly or during workup, leads to the main product *pic*[**Ru**_**4**_ ***C*** ^***Me***^_**3**_]*pic*. The somewhat lower yield of 74% might be explained by a rather slow dissociation of **C** ^**Me**^, which already makes the capping toward *pic*[**Ru**_**3**_ ***C*** ^***Me***^_**2**_]*pic* competitive. Due to higher solubility, this presumed side product might be washed away during purification.

At 100 °C however, the situation becomes more complex. The higher thermal energy of the molecules, in particular without any stabilizing dual‐coordination effect, might be sufficient to induce temporary [Ru]–*pyr* bond cleavage at both *pic* and **C** ^**Me**^ ligands, thus leading to fast fragmentation into smaller [**Ru**_**2**_] and [**Ru**_**3**_] species (see proposed mechanism in Figure 6b). Due to the fast consumption of **1**‐**C** ^**Me**^ via either coordination or fragmentation, these intermediates cannot be further elongated but rather recombine into *pic*[**Ru**_**4**_ ***C*** ^***Me***^_**3**_]*pic* as the kinetically controlled main product upon the completed exchange of weak *dmso* with free *pyr* ligands.

Concerning the subtle balance between elongation and capping for the regular mechanism, we observed a very intriguing concentration‐dependence of the oligomer length for both **B** and **C** with a complete switch of the pathway selectivity with a narrow concentration regime. As already mentioned earlier, under more dilute conditions, the first‐order dissociation into *pic*[**Ru**_**3**_ ***L***_**2**_]–□ is rate determining (under the condition that there is still enough **1**‐*L* available for scrambling), which makes the formation of *pic*[**Ru**_**4**_ ***L***_**3**_]*L* via pathway D much faster compared to the second‐order capping reaction via pathway C. After increasing c(Ru)_tot_ over a certain threshold though, the second‐order rate law for the bimolecular capping with **2**‐*pic* becomes dominant, thus facilitating the clean formation of both *pic*[**Ru**_**4**_ ***B***_**3**_]*pic* (entry 3 in Table 1, c(Ru)_tot_ = 23.8 mM) and *pic*[**Ru**_**4**_ ***C***_**3**_]*pic* (entry 6 in Table 1, c(Ru)_tot_ = 7.8 mM). In this context, it is also reasonable that the isolated yield of 74% for *pic*[**Ru**_**4**_ ***C***_**3**_]*pic* is significantly smaller compared to most other reactions. Due to the reduced TET, already the first intermediate *pic*[**Ru**_**2**_ ***C***]*C* might be stable enough to allow some capping toward the usually elusive *pic*[**Ru**_**3**_ ***C***_**2**_]*pic*, which is then removed by washing during the workup. The kinetic pathway control for all reactions is clearly evident by the observation that any reactions deviating from these privileged concentrations only resulted in the isolation of complex oligomer mixtures instead of any monodisperse products.

The decisive role of *pic*[**Ru**_**3**_ ***L***_**2**_]*L* as an *on‐pathway* intermediate for both pathways C and D was further shown by the surprising observation that the length of the isolated oligomer could also be time dependent. While the reaction between **1**‐***B*** and **2**‐*pic* at c(Ru)_tot_ = 11.9 mM for 19 h cleanly afforded *pic*[**Ru**_**5**_ ***B***_**4**_]*pic* (entry 2, Table S4, Supporting Information), quenching and workup after just 6 h, surprisingly, resulted in the isolation of pure *pic*[**Ru**_**4**_ ***B***_**3**_]*pic* in 10% yield (entry 8, Table S4, Supporting Information). We explain this further elongation over time with the assumption that *pic*[**Ru**_**3**_ ***B***_**2**_]*B* is indeed a metastable species, which is slowly elongated over the course of several hours under these conditions. When the reaction is already quenched after 6 h, *pic*[**Ru**_**3**_ ***B***_**2**_]*B* is only converted into *pic*[**Ru**_**4**_ ***B***_**3**_]*pic* via reaction with **2**‐*pic* at the increased concentrations upon removal of the solvent via rotavaporation. This *capping‐by‐concentration* approach might also help to finalize the transformation and to increase the yield in other reactions. However, under more forcing conditions, we already saw the emergence of the final oligomer before workup by in‐situ NMR monitoring (see Figure S39, Supporting Information for *pic*[**Ru**_**4**_ ***B***_**3**_]*pic*).

As another probe for the TET and the σ‐donor strength of the linkers, OMe‐substituted **1** *‐**C*** ^**OMe**^ only afforded *pic*[**Ru**_**4**_ ***C*** ^***OMe***^_**3**_]*pic* over a concentration range of 3.9–7.8 mM. We attribute this different behavior compared to parent linker **C** to two factors. On the one hand, the steric demands of the OMe groups increase the torsion angle for the inner phenylene, thus weakening the conjugation along the linker and further reducing the TET, which basically shuts off pathway D due to even slower dissociation kinetics compared to **C**. On the other hand, the inductive effect of the electron‐donating OMe groups enhances the σ‐donor strength, which makes the *pyr* sites more reactive and accelerates the capping reaction toward *pic*[**Ru**_**4**_ ***C*** ^***OMe***^_**3**_]*pic*. Even at higher dilution, we could not induce the clean formation of a Ru–*5*–mer for this ligand (Table S7, Supporting Information).

The change of the endcap from *pic* to 4‐dimethylaminopyridine (*dmap*) illustrates the importance of a fast and reliable capping reaction for facile oligomer formation. Reaction between **1**‐***B*** and **2**‐*dmap* at 80 °C showed only incomplete conversion and no pure oligomer could be isolated. However, reaction in a microwave reactor at 100 °C under otherwise identical conditions (entry 7 in Table 1) proceeded smoothly with *dmap*[**Ru**_**4**_ ***B***_**3**_]*dmap* as the only product over a concentration range of 3.9–7.8 mM (Table S5, Supporting Information). Based on the proposed mechanism, we explain this outcome by a weaker KTE for *dmap*, which is a strong σ‐donor but a π‐donor instead of a π‐acceptor.^[^ ^35^ ^]^ This effect increases the π‐back bonding of the *trans* ligand, which hampers dissociation of *dmso* in **2**‐*dmap* and requires higher temperatures for facile activation of the capping reagent. Once the transient species *dmap*[**Ru**]–□ is formed however, it is more reactive and favors, in particular at higher temperatures, capping via pathway C toward the isolated product *dmap*[**Ru**_**4**_ ***B***_**3**_]*dmap* instead of elongation via pathway D. The overall lower reactivity of this endcap is however manifested in the lower yield of only 81% for the main oligomer and a lower purity of the soluble by‐product **1**‐*dmap*. While the second product **1**‐*pic* is typically the only detectable species in ^1^H NMR spectra of the soluble fractions for all reactions with **2**‐*pic*, significant amounts of unreacted **2**‐*dmap* and some other, presumably partially reacted, fragments are still present after workup in the case of *dmap* as endcap (Figure S36, Supporting Information). As both the *dmap* endcap and elongated linkers **C** or **C** ^**Me**^ seem to favor shorter oligomers, we also tested the combination of these building blocks. Interestingly, reactions between **2**‐*dmap* and **1**‐***C*** or **1**‐***C*** ^**Me**^ at 100 °C in a microwave reactor only afforded oligomer mixtures as evidenced by broadened and undefined ^1^H NMR spectra (Figure S46, Supporting Information). In view of the pronounced signal overlap, we can only estimate that a tetranuclear oligomer and a mixture of tri‐ and tetranuclear oligomers were isolated as the main products in the **1**‐**C** ^**Me**^/**2**‐*dmap* and **1**‐**C**/**2**‐*dmap* combinations, respectively. While these observations are again in accordance with the suggested higher reactivity of the transient species *dmap*[**Ru**]–□, the initially intended Ru–*3*–mer still remains elusive.

Overall, the interacting network of competing and connected reactions of the proposed mechanism provides a comprehensive and conclusive picture for oligomer formation, which is fully consistent with all experimental results. In our view, this complex chemical system is an impressive manifestation of the universal, however sometimes neglected, fact that both kinetic and thermodynamic properties are usually crucial for the full control over multistep procedures in metallosupramolecular chemistry.

## 3 Conclusion

We have presented the synthesis and NMR characterization of a series of monodisperse tetra‐ or pentanuclear Ru(bda) coordination oligomers *pic*[**Ru**_***n***_ ***L***_***n*** **−1**_]*pic* (*n* = 4 or 5) with varying bridging ligands *L*. Instead of the intended Ru‐*3*‐mers, the reaction of mononuclear core units **1**‐*L* with endcaps **2**‐*cap* in TFE at elevated temperatures solely yielded the scrambled products bearing four or five Ru centers in high purity and excellent yields of more than 90% under precisely optimized conditions. Intriguingly, the length of the isolated oligomers crucially depends on the concentration or reaction temperature and may even be completely switched within rather narrow limits. Detailed mechanistic studies revealed a complex network of interconnected reactions that accounts for all experimental observations and fully explains the kinetic pathway control. While the conversion of the precursors is driven by the thermodynamically favored replacement of weak *dmso* with strong *pyr* ligands, the outcome and product distribution are governed by the varying kinetics of the competing dissociation‐elongation and capping pathways. Such a holistic analysis of both kinetic and thermodynamic features for multi‐step metallosupramolecular assemblies provides unprecedented insight into the complex formation mechanism. We anticipate that such a combined approach may also be transferred to various other multinuclear coordination compounds, thus allowing for a highly demanded control over product distribution and reaction outcome. For the presented oligomer series, we currently investigate the effects of oligomer length and electronic properties on the performance of such materials in electrochemical water oxidation and these results will be reported in due course.

## Conflict of Interest

The authors declare no conflict of interest.

## Author Contributions

**Tilman Schneider**: conceptualization (equal); data curation (lead); formal analysis (lead); investigation (lead); methodology (lead); writing—original draft (lead); writing—review and editing (supporting). **Florian Seebauer**: investigation (equal); methodology (equal). **Frank Würthner**: conceptualization (lead); funding acquisition (lead); project administration (lead); resources (lead); supervision (lead); validation (supporting); writing—review and editing (equal). **Florian Beuerle**: conceptualization (equal); project administration (lead); supervision (lead); validation (lead); writing—original draft (supporting); writing—review and editing (lead).

## Supporting information

Supplementary Material

## Data Availability

The data that support the findings of this study are available from the corresponding author upon reasonable request.
