# Supplementary material for: Kinetic Pathway Control in the Synthesis of Well‐Defined Ruthenium Coordination Oligomers
Source: Small Sci. 2025 Feb 3;5(5):2400504. doi: 10.1002/smsc.202400504 (PMC12087777; doi:10.1002/smsc.202400504)
Supplement: Supplementary file 1 — Supplementary Material [file SMSC-5-2400504-s001.pdf]

Supporting Information

Kinetic Pathway Control in the Synthesis of Well-Defined Ruthenium Coordination Oligomers

Tilman Schneider,[a,b] Florian Seebauer,[a] Frank Würthner*[a,b] and Florian Beuerle,*[a,b,c]

[a] Julius-Maximilians-Universität Würzburg, Institut für Organische Chemie, Am Hubland, 97074 Würzburg, Germany

[b] Julius-Maximilians-Universität Würzburg, Center for Nanosystems Chemistry (CNC), Theodor-Boveri-Weg, 97074 Würzburg, Germany

[c] Eberhard Karls Universität Tübingen, Institut für Organische Chemie, Auf der Morgenstelle 18, 72076 Tübingen, Germany

*E-mail: wuerthner@uni-wuerzburg.de, florian.beuerle@uni-tuebingen.de,

Content

1 **Materials** S2

2 **Technical Equipment** S2

3 **Synthetic Procedures and Characterization** S3

4 **Screening of Reaction Conditions** S35

5 **Diffusion-Ordered NMR Spectroscopy** S47

6 **Molecular Modelling** S63

7 **References** S69

1 Materials

All reactions were carried out in standard glass equipment. All chemicals were purchased from usual commercial suppliers and used without further purification, unless stated otherwise. Conventional solvents were distilled prior to use. Silica gel 60 M (particle size: 0.04−0.063 mm) for column chromatography was purchased from MACHERY-NAGEL. Alumina (EcoChrom™ MP Alumina N, activity level I) for column chromatography was obtained from MP BIOMEDICALS GERMANY and deactivated to activity level V ("AlOx V") by addition of water (15 wt%) prior to use. Deuterated solvents for NMR spectroscopy were purchased from EURISOTOP, SIGMA-ALDRICH, MERCK MILLIPORE and DEUTERO.

2 Technical Equipment

NMR spectroscopy: BRUKER AVANCE 400; DOSY-NMR: BRUKER AVANCE 600. Chemical shifts are given in ppm in relation to the residual protonated solvent signal as internal standard (^{1}H-NMR: 7.26 ppm for $CDCl_3$, 2.50 ppm for DMSO-d_6, 5.32 ppm for dichloromethane-d_2, 3.31 ppm for methanol-d_4, 7.58 ppm for pyridine-d_5, 3.88 ppm for trifluoroethanol-d_3; ^{13}C-NMR: 53.84 ppm for dichloromethane-d_2, 49.00 ppm for methanol-d_4). [S1]

NMR spectra of Ru oligomers were recorded with addition of a small quantity of ascorbic acid in order to prevent the formation of paramagnetic Ru^{III}, which would hamper NMR measurements. [S2]

Signal multiplicities are reported as s (singlet), d (doublet), t (triplet), q (quartet) and m (multiplet) with the chemical shift at the center of the signal. Complex signals are reported as the range of the occurring signals. The addition *(br)* indicates a broad signal. Processing of the raw data was performed with the program Topspin (versions 3.5 or 4.1, respectively). [S3]

MALDI-TOF mass spectrometry: ultrafleXtreme BRUKER DALTONICS, matrix: DCTB (*trans*-2-(3-(4-*t*-Butylphenyl)-2-methyl-2-propenylidene)malononitrile).

ESI mass spectrometry: micrOTOF-Q III BRUKER DALTONICS, positive mode, solvent: $CHCl_3$/MeCN 1:1.

IR spectroscopy: FT/IR-4600 JASCO, ATR mode.

Elemental analysis: unicube CHNS ELEMENTAR.
Melting points: SMP50, STUART.

Microwave-assisted synthesis: Discover, CEM.

3 Synthetic Procedures and Characterization

General remarks: All reactions were carried out under an inert atmosphere of argon. The following literature-known substances and precursors were synthesized according to reported procedures: [Ru(bda)(dmso)$_2$] (**1**-*dmso*) [S4] (including precursors *bda* [S5] and [$RuCl_2(dmso)_4$] [S6]), **1-B**, [S7] **C**, [S8] **C**$^{\mathbf{Me}}$, [S9] and **C**$^{\mathbf{OMe}}$. [S10] Analytical data for all reproduced syntheses were in good accordance with reported information.

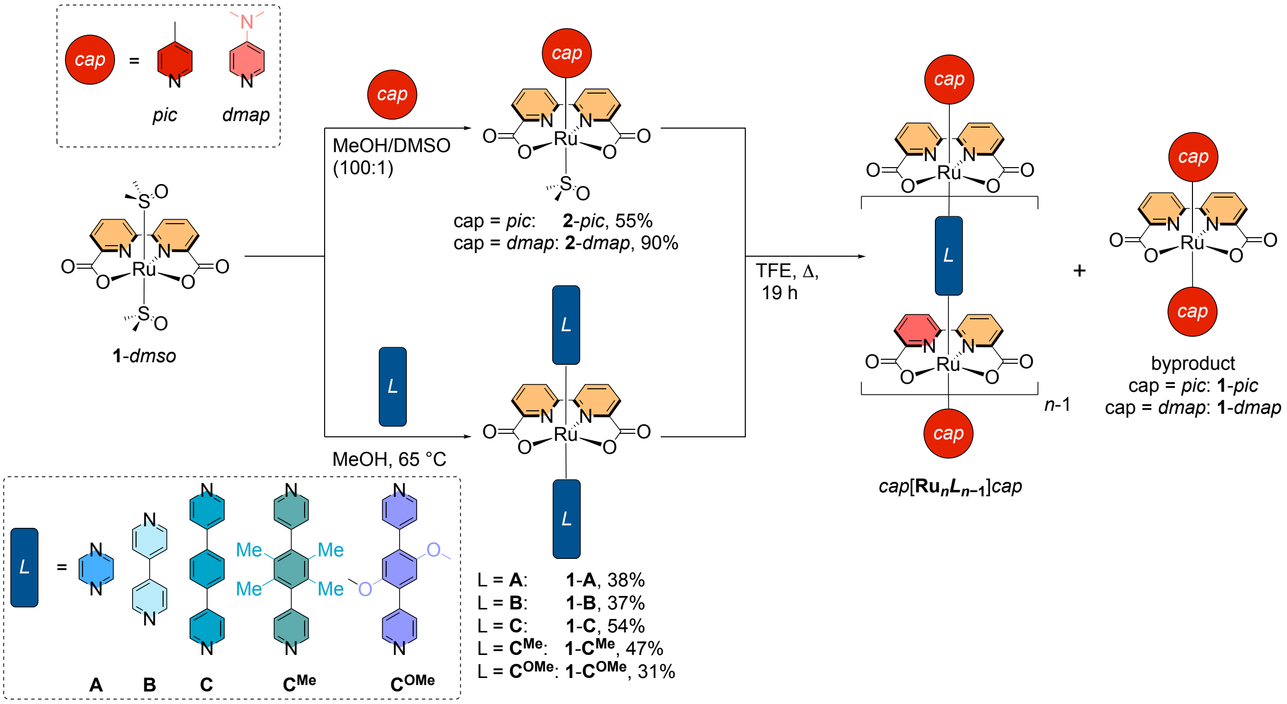

Scheme S1. General scheme for the synthesis of Ru(bda) coordination oligomers.

[Ru(bda)(pic)(dmso)], *pic*[**Ru**]*dmso* (**2**-*pic*)

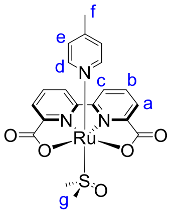

2-*pic*
Chemical Formula: $C_{20}H_{19}N_3O_5RuS$
Molecular Weight: 514.52

Synthesis was carried out similar to a literature procedure and analytical data were in good accordance with reported information. [S11]

[Ru(bda)(dmso)$_2$] (**1**-*dmso*) (250 mg, 500 µmol, 1.0 eq.) and 4-picoline (50.0 µL, 0.9548 g cm^{-3}, 500 µmol, 1.0 eq.) were dissolved in degassed methanol (24 mL) and anhydrous DMSO (240 µL). The mixture was heated at 65 °C for 25 minutes. After cooling down to room temperature, the solvent was removed under reduced pressure and the residue was purified by column chromatography (Al_2O_3, 15% w/w H_2O, DCM to DCM/methanol 95:5).

Yield: 141 mg (275 µmol, 55%) of a dark red solid.

^{1}H-NMR (400 MHz, methanol-*d*$_4$): δ = 8.56-8.60 (m, 2H, H_a), 8.15-8.05 (m, 4H, $H_{b,c}$), 7.75 (d, $^3J_{d,e}$ = 6.4 Hz, 2H, H_d), 7.18 (d, $^3J_{d,e}$ = 6.4 Hz, 2H, H_e), 2.89 (s, 6H, H_g), 2.31 (s, 3H, H_f) ppm.

[Ru(bda)(dmap)(dmso)], *dmap*[**Ru**]*dmso* (**2**-*dmap*)

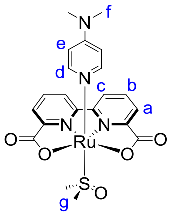

2-*dmap*
Chemical Formula: $C_{21}H_{22}N_4O_5RuS$
Molecular Weight: 543.56

[Ru(bda)(dmso)$_2$] (**1**-*dmso*) (100 mg, 200 µmol, 1.0 eq.) and *N*,*N*-dimethyl-4-aminopyridine (24.4 mg, 200 µmol, 1.0 eq.) were dissolved in a degassed mixture of methanol (10 mL) and DMSO (0.1 mL). The reaction mixture was heated to 65 °C for 25 min in a pre-heated oil bath. Afterwards, the reaction mixture was cooled to room temperature, filtered and the solvent was removed under reduced pressure. The crude product was purified by column chromatography (Al_2O_3, 15% w/w H_2O, gradient DCM/MeOH 1-20%).

Yield: 97.6 mg (180 µmol, 90%) of a brown solid.

^{1}H-NMR (400 MHz, methanol-d_4): δ = 8.55-8.53 (m, 2H, H_a), 8.12-8.06 (m, 4H, $H_{b,c}$), 7.32 (d, $^3J_{d,e}$ = 7.4 Hz, 2H, H_d), 6.48 (d, $^3J_{d,e}$ = 7.4 Hz, 2H, H_e), 2.94 (s, 6H, H_f), 2.88 (s, 6H, H_g) ppm.

^{13}C-NMR (101 MHz, methanol-d_4): δ = 174.6, 159.7, 156.6, 155.6, 149.5, 136.1, 127.4, 127.3, 125.7, 108.6, 42.4, 39.2 ppm.

MS (MALDI-TOF, DCTB, chloroform/methanol, pos): m/z = 544.04117 [M^+]; calc. 544.035433.

M.p.: 310 °C (decomp.).

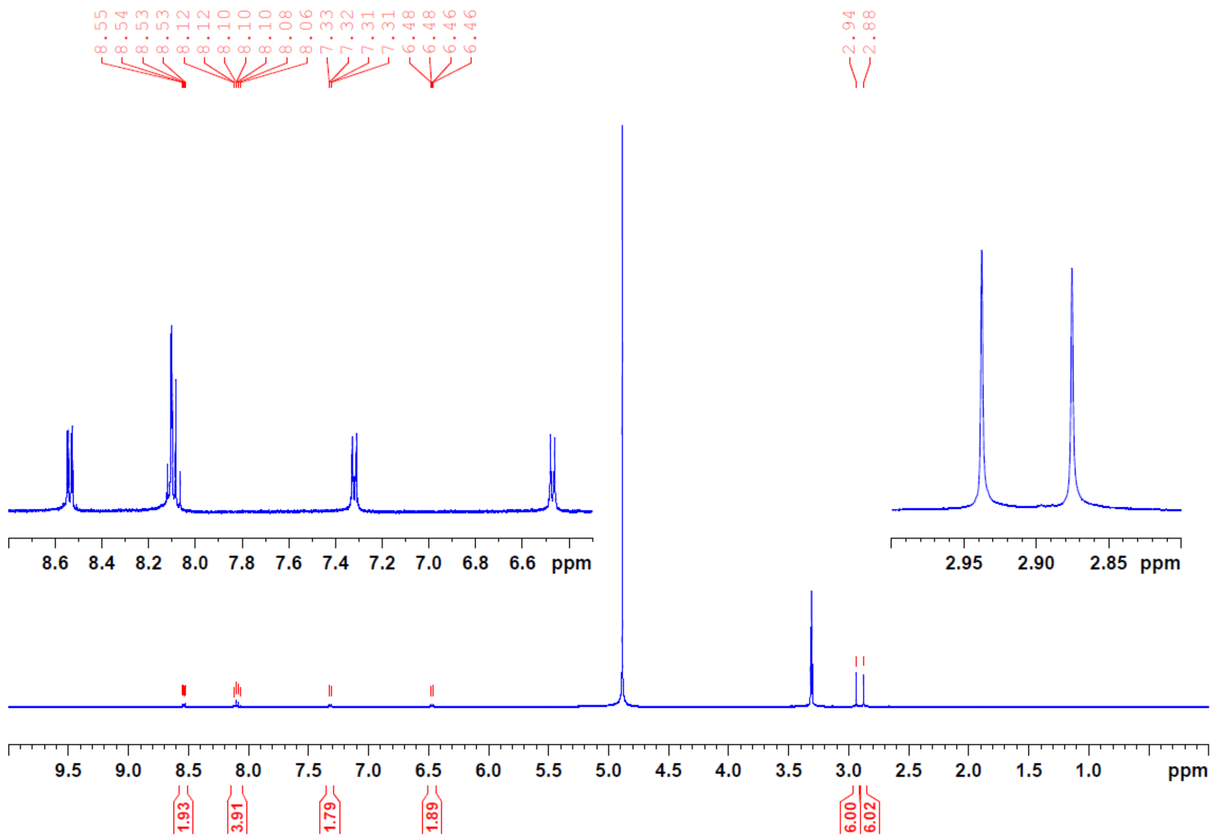

Figure S1. ^{1}H-NMR spectrum (400 MHz, methanol-d_4, rt) of complex **2**-*dmap*.

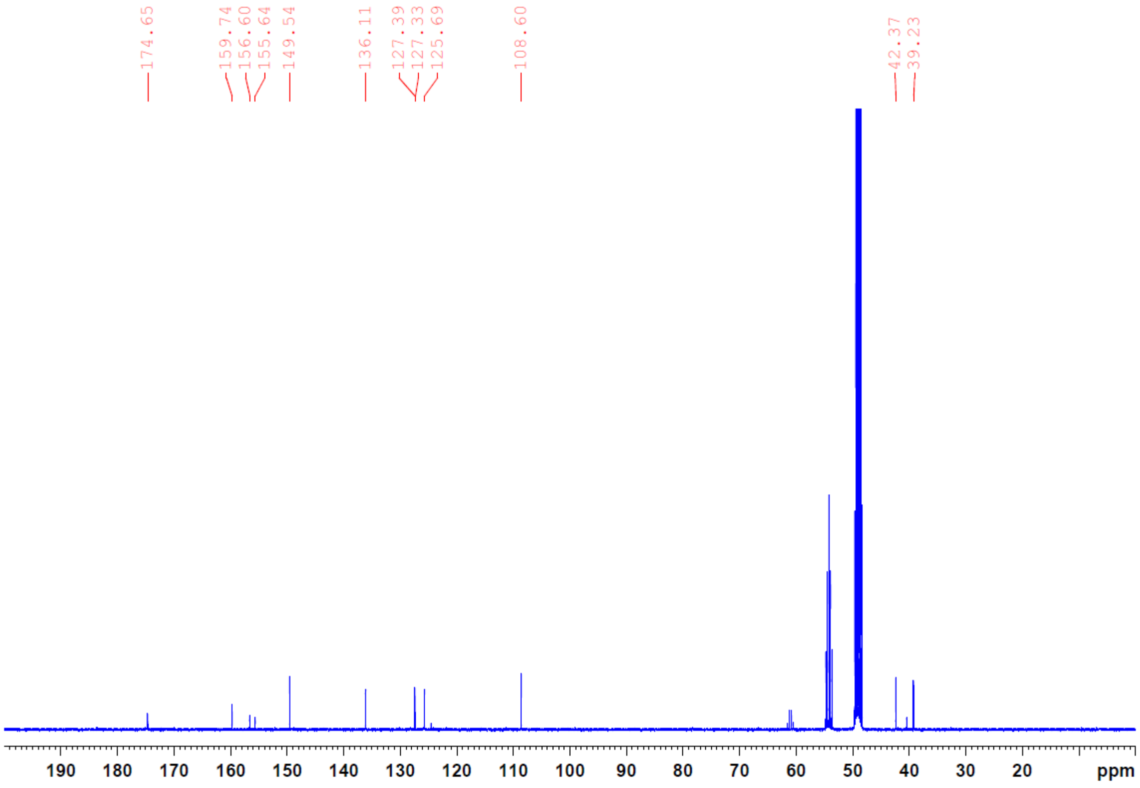

Figure S2. $^{13}C\{^{1}H\}$-NMR spectrum (101 MHz, methanol-d_4, rt) of complex **2**-*dmap*.

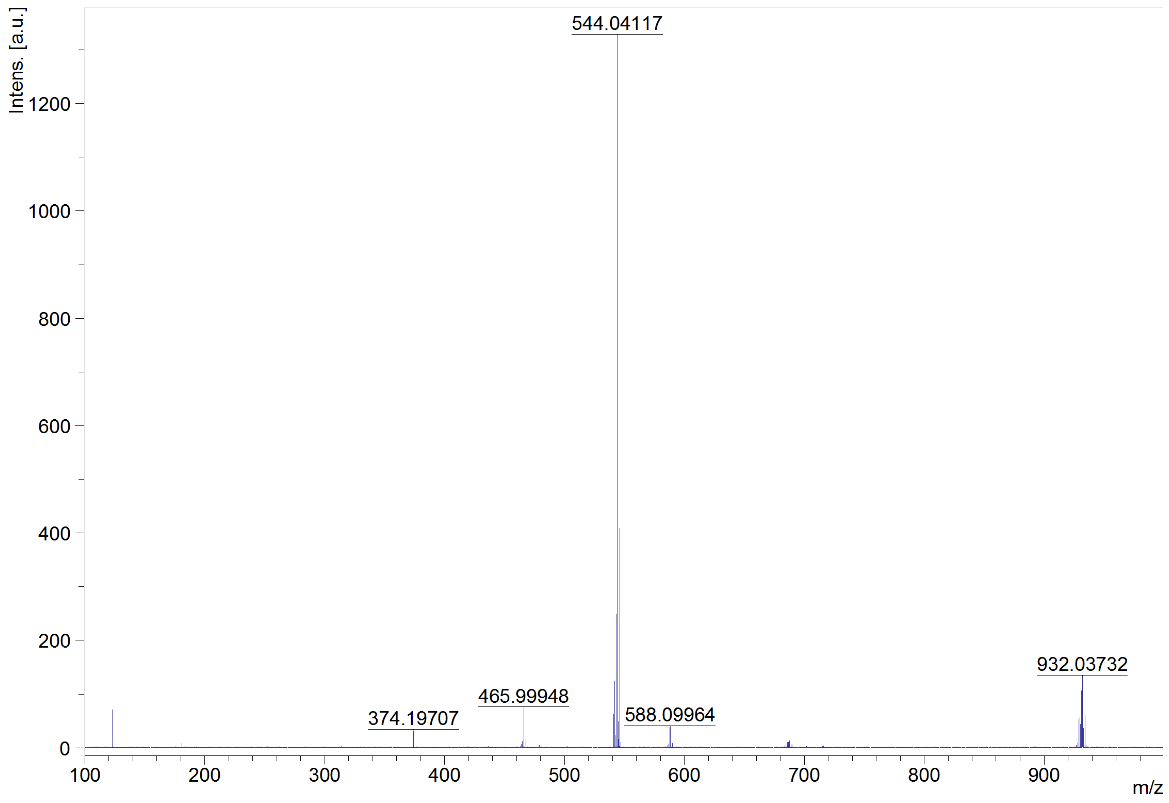

Figure S3. MALDI-TOF spectrum (DCTB, MeOH/$CHCl_3$, positive mode) of complex **2**-*dmap*.

[Ru(bda)(1,4-bis-(pyridin-4‘-yl)benzene)$_2$], *C*[**Ru**]*C* (**1-C**)

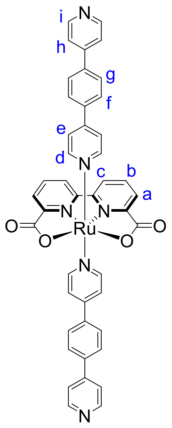

1-C
Chemical Formula: $C_{44}H_{30}N_6O_4Ru$
Molecular Weight: 807.83

1-*dmso* (150 mg, 300 µmol, 1.0 eq.) and 1,4-bis-(pyridin-4’-yl)benzene (**C**) (1.40 g, 10.0 mmol, 20.0 eq.) were dissolved in degassed methanol (50 mL) and stirred for 1 hour at 70 °C. After cooling to room temperature, the solvent was removed under reduced pressure and the residue was purified by twofold column chromatography (1. Al_2O_3, 15% w/w H_2O, DCM to DCM/methanol 95:5, 2. SiO_2, DCM to DCM/methanol 95:5).

Yield: 65.3 mg (81.0 µmol, 54%) of a dark red solid.

^{1}H-NMR (400 MHz, CD_2Cl_2/CD_3OD 4:1, ascorbic acid): δ = 8.57 (d, ^3J = 6.2 Hz, 4H, H_i), 8.38 (d, ^3J = 7.8 Hz, 2H, H_c), 8.07 (d, ^3J = 7.8 Hz, 2H, H_a), 7.86 (d, ^3J = 6.2 Hz, 4H, H_e), 7.83 (t, ^3J = 7.8 Hz, 2H, H_b), 7.75 (d, ^3J = 8.5 Hz, 4H, H_g), 7.67 (d, ^3J = 8.5 Hz, 4H, H_f), 7.59 (d, ^3J = 6.2 Hz, 4H, H_h), 7.39 (d, ^3J = 6.2 Hz, 4H, H_d) ppm.

^{13}C-NMR (100 MHz, CD_2Cl_2 / CD_3OD (4:1), ascorbic acid): δ = 174.3, 172.6, 160.4, 155.1, 153.7, 152.9, 149.9, 148.5, 137.7, 132.4, 128.5, 128.3, 126.8, 123.0, 122.6, 119.2 ppm.

MS (ESI, pos., MeOH/DCM 1:5): *m/z* calculated for $[M]^{+\cdot}$: 831.12761, found: 831.12512.

M.p.: > 400 °C.

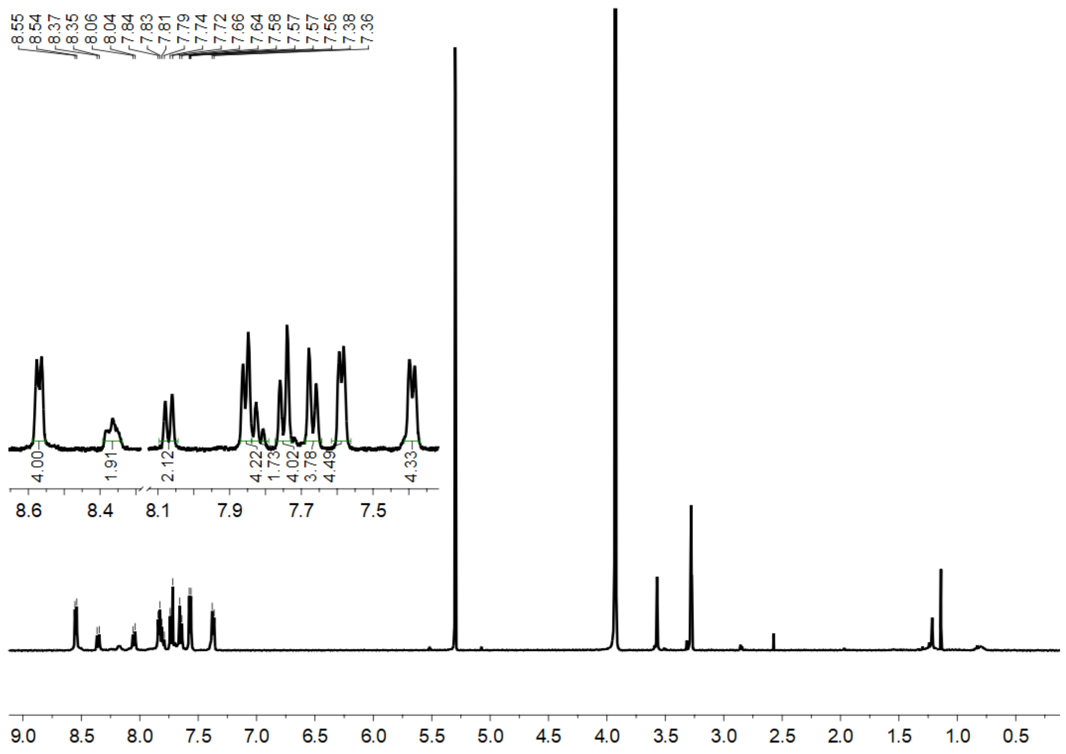

Figure S4. ^{1}H-NMR spectrum (400 MHz, CD_2Cl_2/CD_3OD 4:1, ascorbic acid, 298 K) of **1-C**.

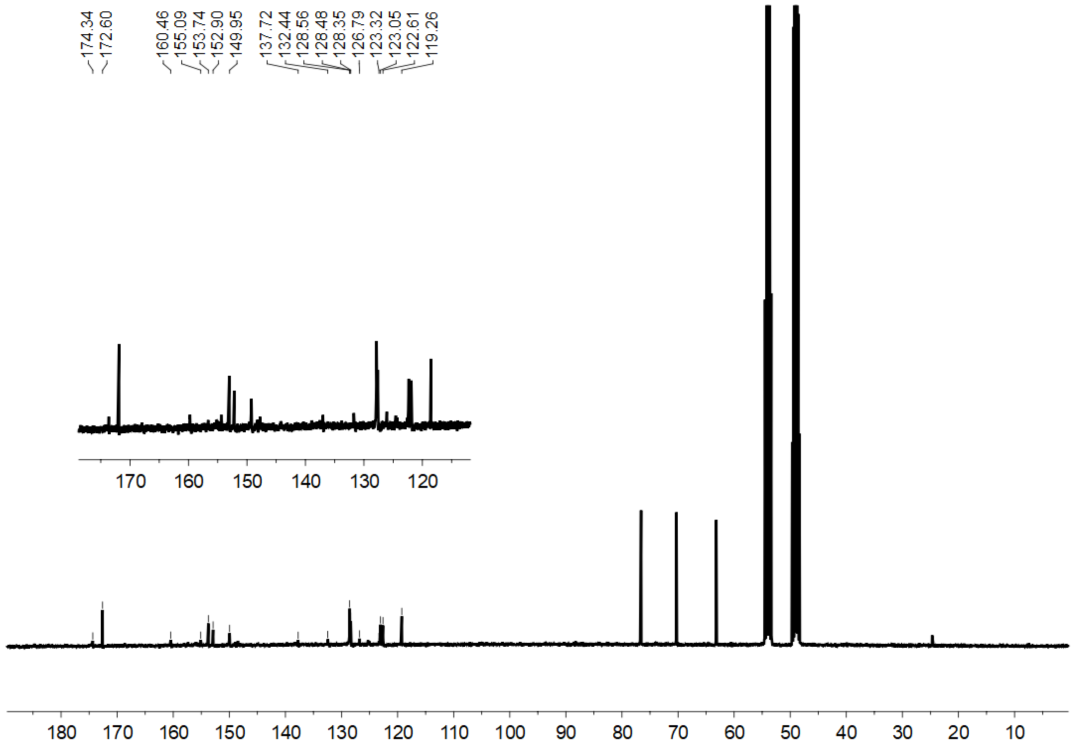

Figure S5. ^{13}C{^{1}H}-NMR spectrum (101 MHz, CD_2Cl_2/CD_3OD 4:1, ascorbic acid, 298 K) of **1-C**.

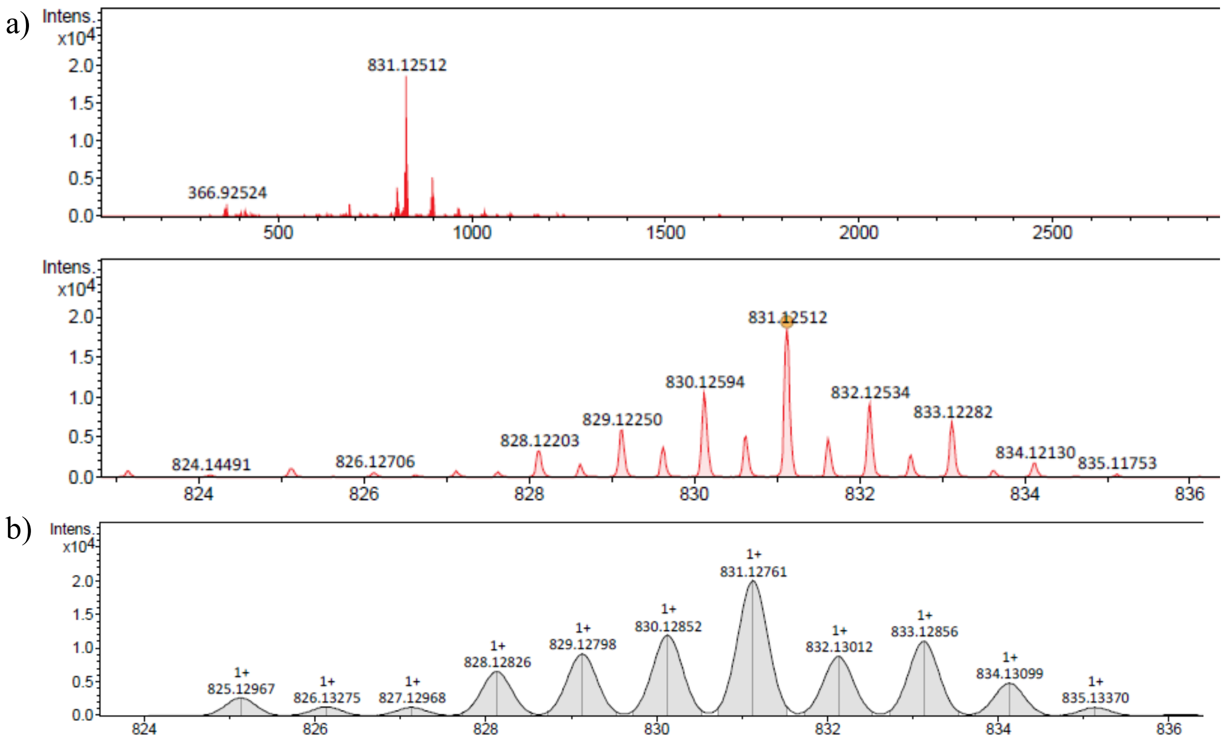

Figure S6. a) ESI-MS spectrum (MeCN:$CHCl_3$ 1:1, positive mode) of **1-C** and b) calculated isotopic mass pattern.

[Ru(bda)($\mathbf{C^{Me}}$)$_2$], C^{Me}[**Ru**]C^{Me} (**1-C$^{\mathbf{Me}}$**)

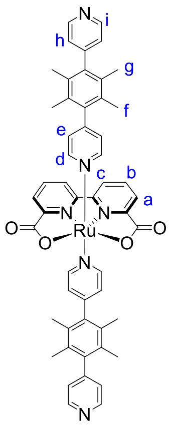

1-C$^{\mathbf{Me}}$

Chemical Formula: $C_{52}H_{46}N_6O_4Ru$

Molecular Weight: 920.05

[Ru(bda)(dmso)$_2$] (**1**-*dmso*) (86.6 mg, 173 µmol, 1.0 eq.) and $\mathbf{C^{Me}}$ (1.0 g, 3.47 mmol, 20 eq.) were dissolved in degassed MeOH (50 mL) and heated to reflux for 1 hour in a pre-heated oil bath. Afterwards, the solvent was removed under reduced pressure and the crude product was purified by column chromatography (Al_2O_3, 15% w/w H_2O, gradient DCM to DCM/MeOH 5%).

Yield: 75.0 mg (81.5 µmol, 47%) of a brown solid.

^{1}**H-NMR** (400 MHz, DCM-d_2/methanol-d_4 4:1): δ = 8.63 (dd, 4H, H_i), 8.23 (d, 2H, H_c), 8.14 (d, 2H, H_a), 7.89 (d, 4H, H_d), 7.77 (t, 2H, H_b), 7.04 (dd, 4H, H_h), 6.92 (d, 4H, H_e), 1.84 (s, 12H, H_g), 1.78 (s, 12H, H_f) ppm.

^{13}**C-NMR** (101 MHz, DCM-d_2/methanol-d_4 4:1): δ = 173.1, 160.2, 158.3, 152.2, 151.7, 150.7, 150.4, 140.1, 138.0, 131.8, 131.5, 130.7, 126.4, 126.1, 124.9, 123.6, 18.2, 17.9 ppm.

MS (ESI, $CHCl_3$/MeCN 1:1, pos): *m/z* calculated for [M^+]: 920.262394, found: 920.83268.

M.p.: 287 °C (decomp.).

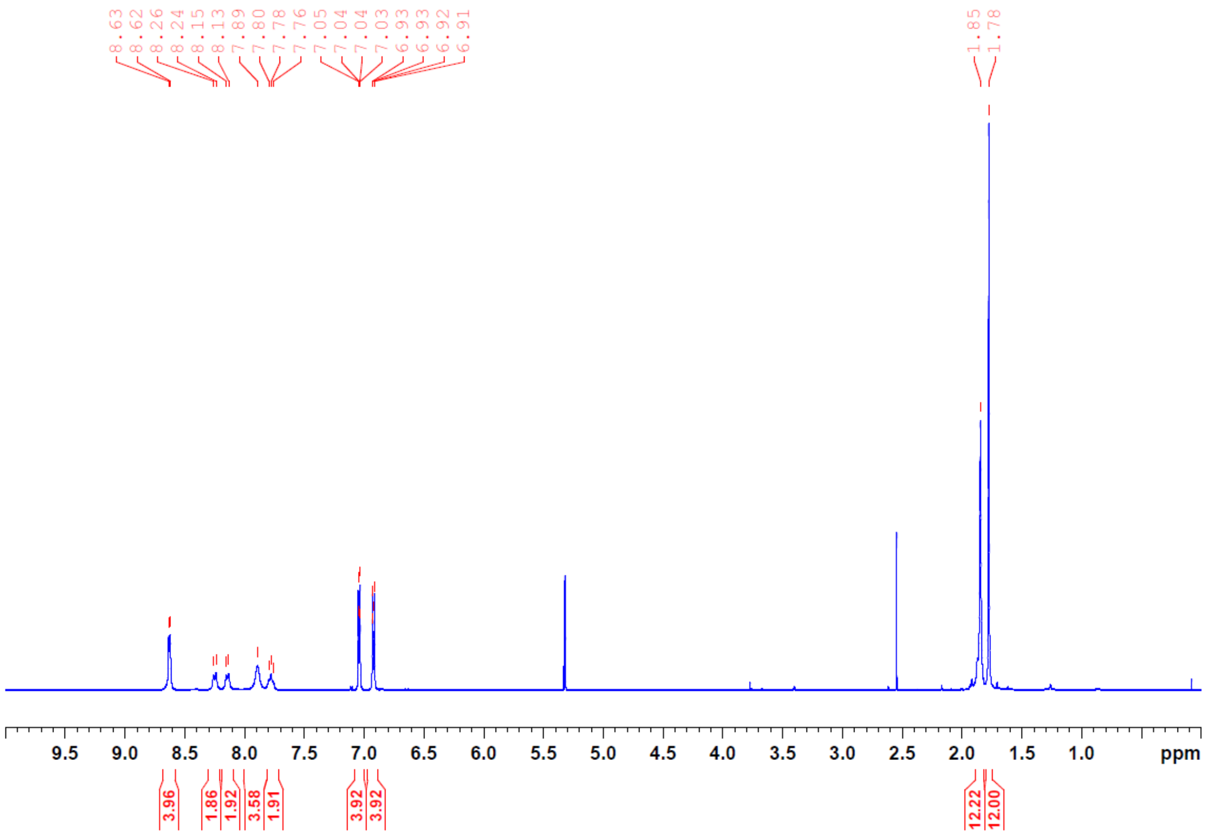

Figure S7. ^{1}H-NMR spectrum (400 MHz, CD_2Cl_2, 298 K) of **1-C^{Me}**.

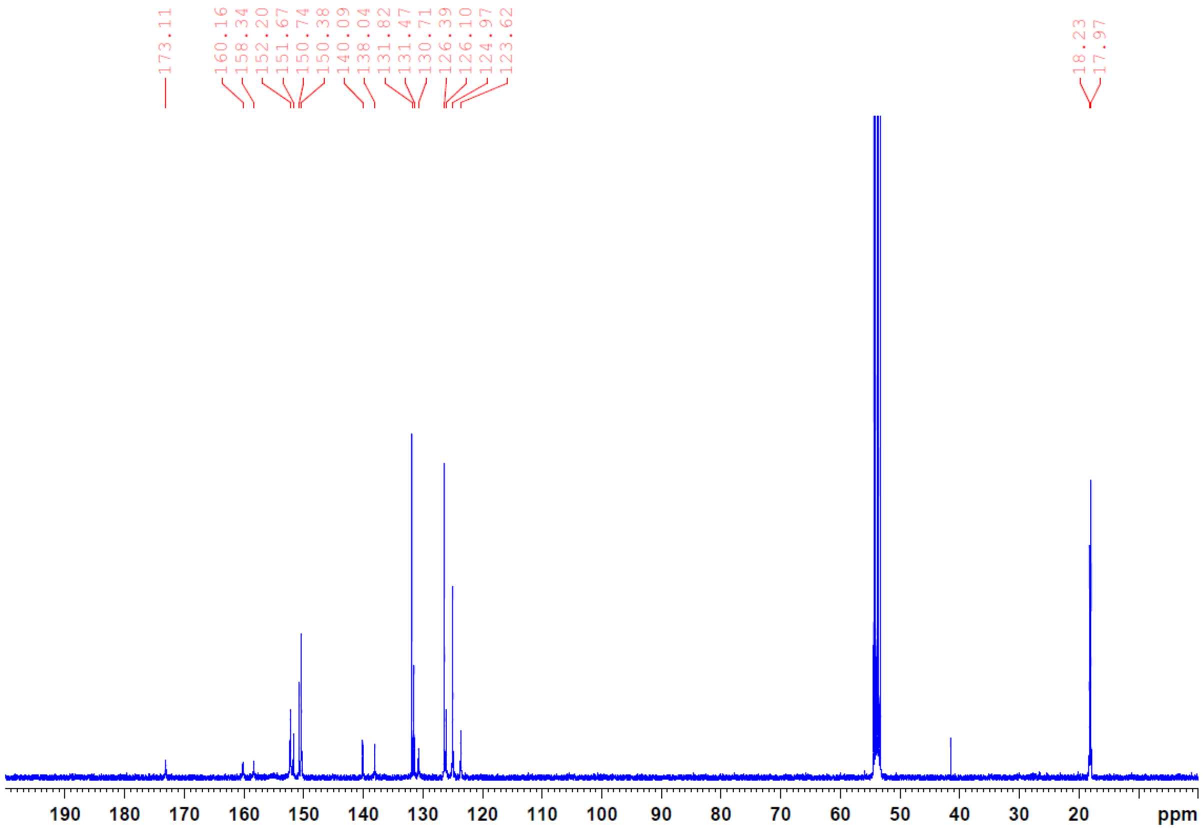

Figure S8. $^{13}C\{^1H\}$-NMR spectrum (101 MHz, CD_2Cl_2, 298 K) of **1-C^Me^**.

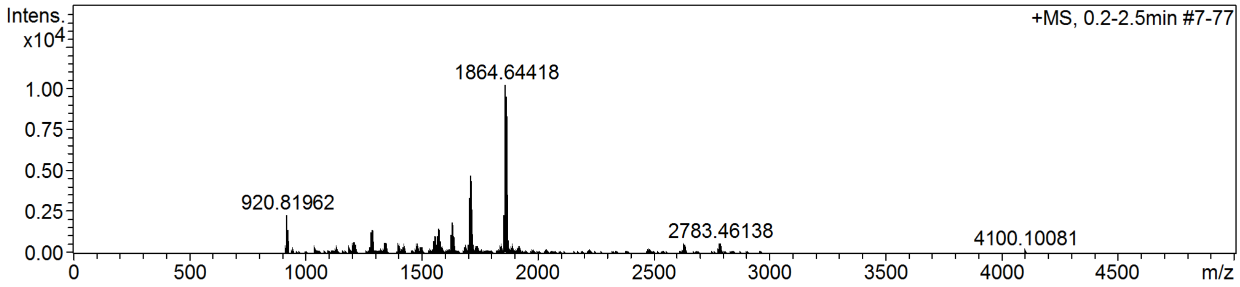

Figure S9. ESI-MS spectrum (chloroform/acetonitrile, positive mode) of **1-C^Me^**.

[Ru(bda)($\mathbf{C}^{\mathbf{OMe}}$)$_2$], C^{OMe}[**Ru**]C^{OMe} (**1-C**$^{\mathbf{OMe}}$)

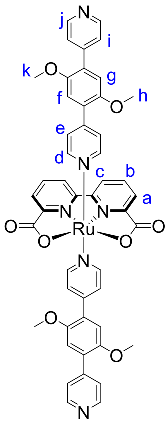

1-C$^{\mathbf{OMe}}$

Chemical Formula: $C_{48}H_{38}N_6O_8Ru$

Molecular Weight: 927.94

[Ru(bda)(dmso)$_2$] (**1**-*dmso*) (150 mg, 300 µmol, 1.0 eq.) and $\mathbf{C}^{\mathbf{OMe}}$ (1.75 g, 6.00 mmol, 20 eq.) were dispersed in degassed MeOH (50 mL) and heated to reflux for 1 hour. After cooling to room temperature, the solvent was removed under reduced pressure and the residue was purified twice by column chromatography (Al_2O_3, 15% w/w H_2O, gradient DCM to DCM/ 10% MeOH).

Yield: 86.3 mg (93.0 µmol, 31%) of a black solid.

^{1}H-NMR (400 MHz, DCM-*d*$_2$): δ = 8.60 (d, 4H, H_j), 8.23 (d, 2H, H_c), 8.09 (s, 2H, H_a), 7.83 (d, 4H, H_d), 7.76 (t, br, 2H, H_b), 7.46 (dd, 4H, H_i), 7.31 (s, 4 H, H_e), 6.94 (s, 2H, H_g), 6.87 (s, 2H, H_f), 3.75 (s, 6H, H_k), 3.74 (s, 6H, H_h) ppm.

^{13}C-NMR (101 MHz, DCM-*d*$_2$): δ = 151.9 (br), 151.2, 150.0, 146.3, 145.9, 145.7, 129.8, 127.1, 125.4 (br), 124.5, 124.4, 114.5, 114.4, 114.1, 56.6, 56.5 ppm.

MS (ESI, $CHCl_3$/MeCN 1:1, pos): *m/z* calculated for [M^+]: 928.179454, found: 928.32744.

M.p.: 320 °C (decomp.).

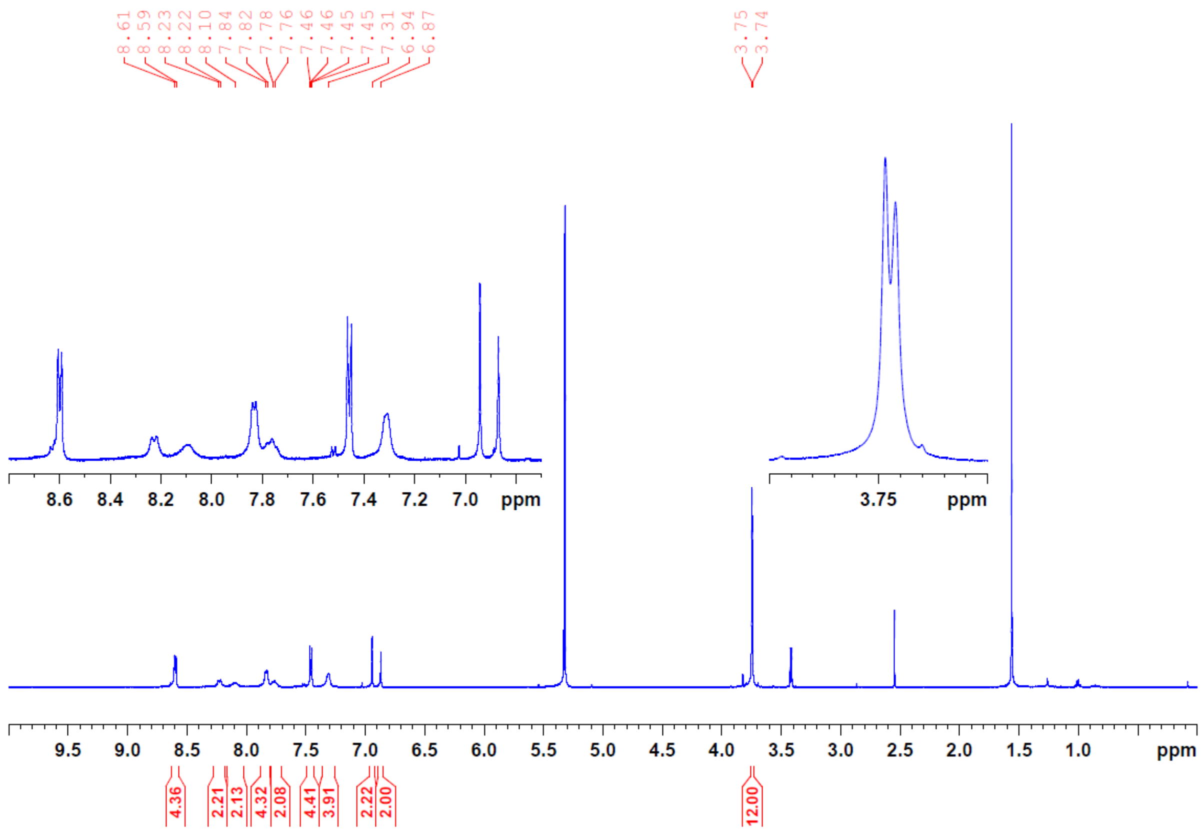

Figure S10. ^{1}H-NMR spectrum (400 MHz, CD_2Cl_2, 298 K) of **1-C^{OMe}**.

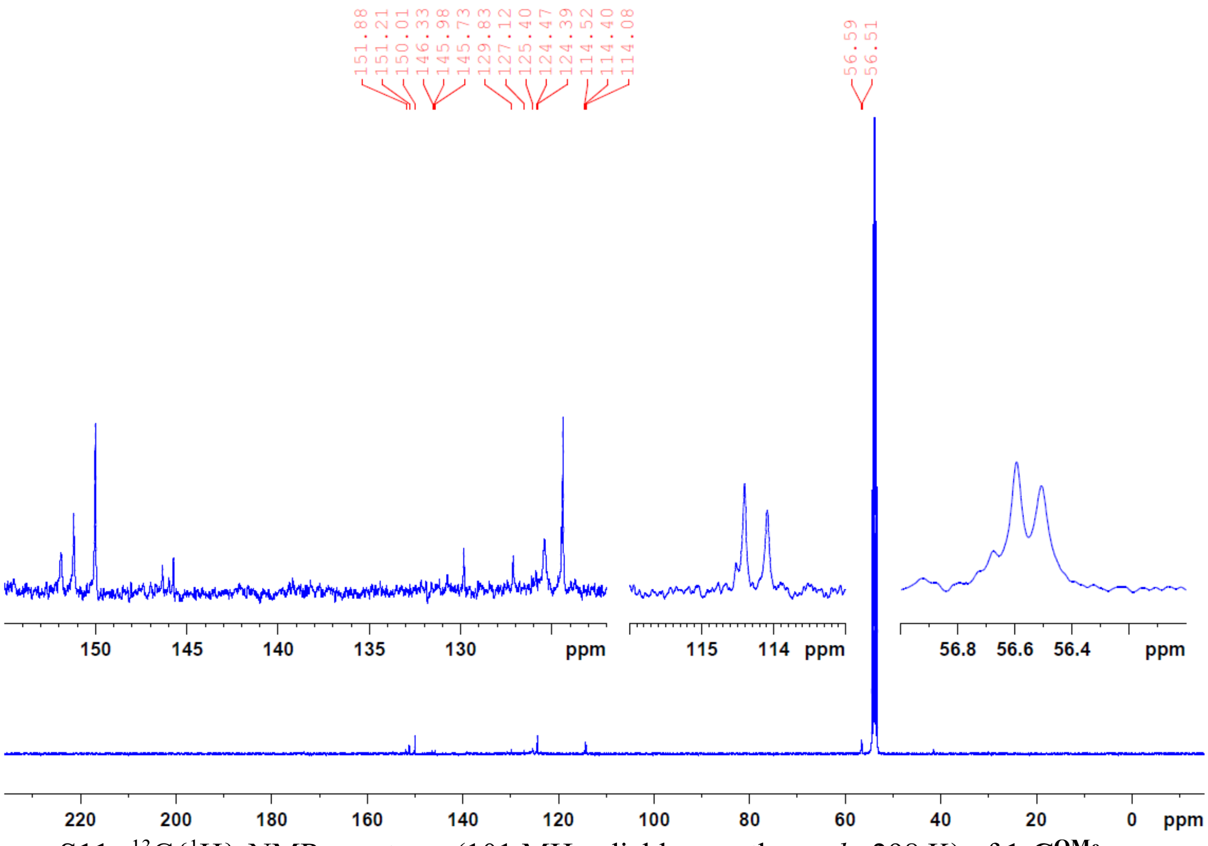

Figure S11. $^{13}C\{^{1}H\}$-NMR spectrum (101 MHz, dichloromethane-d_2, 298 K) of **1-C^{OMe}**.

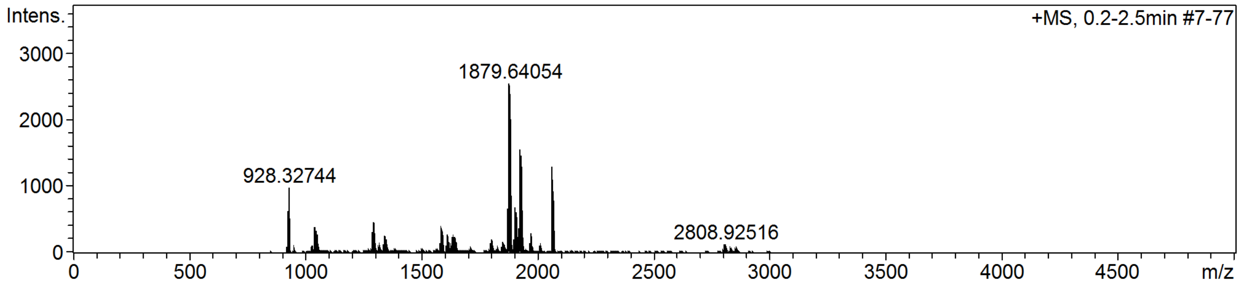

Figure S12. ESI-MS spectrum (chloroform/acetonitrile, positive mode) of **1-C^{OMe}**.

[Ru(bda)(pyz)$_2$], *pyz*[**Ru**]*pyz* (**1-A**)

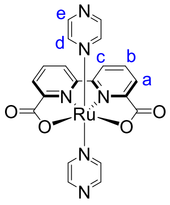

1-A
Chemical Formula: $C_{20}H_{14}N_6O_4Ru$
Molecular Weight: 503.44

[Ru(bda)(dmso)$_2$] (**1**-*dmso*) (200 mg, 1.0 eq.) and pyrazine (640 mg, 20 eq.) were dispersed in degassed MeOH (50 mL). The reaction mixture heated to reflux for 1 hour. Afterwards, the reaction mixture was filtered, and the solvent was removed under reduced pressure at 30 °C bath temperature. The residue was dispersed in ether and filtered again to remove excess pyrazine. The remaining solid was dissolved in MeOH and precipitated by addition of Et_2O. Analytical data was in good accordance with literature values. [S12]

Yield: 75.8 mg (151 µmol, 38%) of a brown solid.

^{1}H-NMR (400 MHz, DMSO-*d*$_6$): δ = 8.75 (d, 2H, H$_c$), 8.42 (d, 4H, H$_e$), 8.03-7.92 (m, 4H, H$_{a,b}$), 7.92 (s (br), 4H, H$_d$).

MS (MALDI-TOF, DCTB, DCM/MeOH, pos): *m/z* calculated for [M$^+$]: 504.011994, found: 504.01901.

[Ru(bda)(pic)(pyz)], *pic*[**Ru**]*pyz* (**S1**)

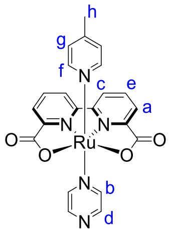

S1

Chemical Formula: $C_{22}H_{17}N_5O_4Ru$

Molecular Weight: 516.48

2-*pic* (50.0 mg, 97.2 µmol, 1.0 eq.) and pyrazine (77.8 mg, 971 µmol, 10 eq.) were dissolved in degassed TFE (17 mL). The mixture was heated to 80 °C for 1 hour. After cooling to room temperature, the solvent was removed under reduced pressure. The residue was purified by column chromatography (Al_2O_3, 15% w/w H_2O, gradient DCM to DCM/MeOH 95:5).

Yield: 15.4 mg (29.8 µmol, 14%) of a brown solid.

^{1}H-NMR (400 MHz, MeOD-*d*$_4$): δ = 8.64 (d, 2H, H_a), 8.33 (d, 2H, H_b), 8.06 (t, 2H, H_c), 8.04 (s, 2H, H_d), 7.97 (t, 2H, H_e), 7.69 (d, 2H, H_f), 7.09 (d, 2H, H_g), 2.28 (s, 3H, H_h).

^{13}C-NMR (151 MHz, MeOD-*d*$_4$): δ = 179.8, 166.0, 162.1, 157.2, 157.1, 156.2, 154.3, 151.4, 139.0, 132.1, 131.5, 25.5 ppm.

MS (MALDI-TOF, DCTB, DCM/MeOH, positive mode): *m/z* calculated for [M$^+$]: 517.032395, found: 517.02228.

M.p.: 395 °C (decomp.).

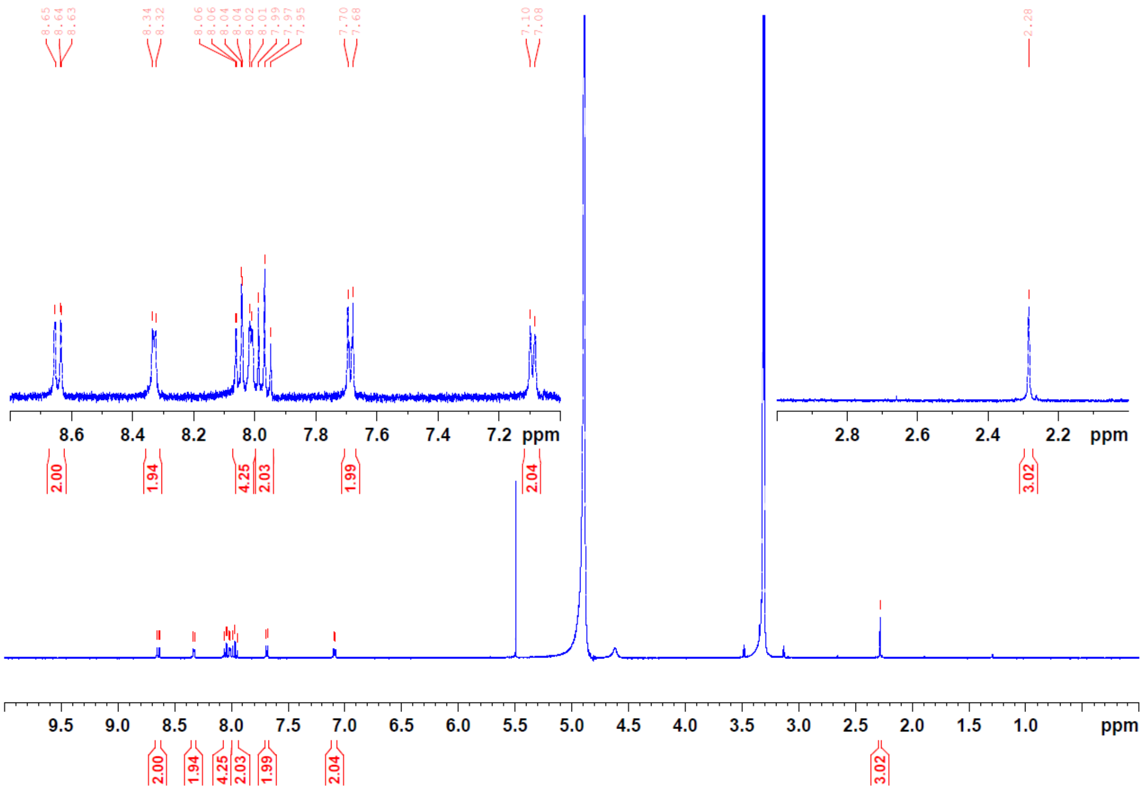

Figure S13. ^{1}H-NMR spectrum (400 MHz, methanol-d_4, 298 K) of complex **S1**.

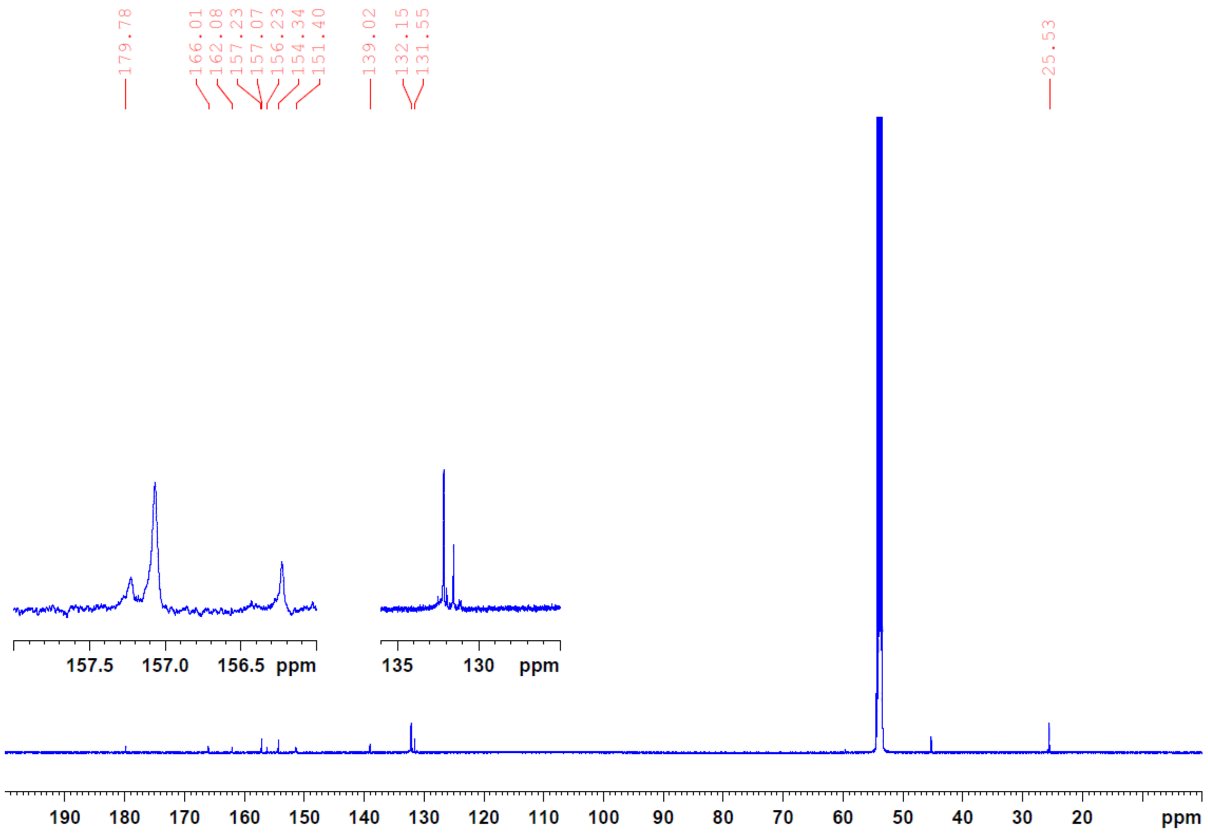

Figure S14. ^{13}C{^{1}H}-NMR spectrum (151 MHz, methanol-d_4, 298 K) of complex **S1**.

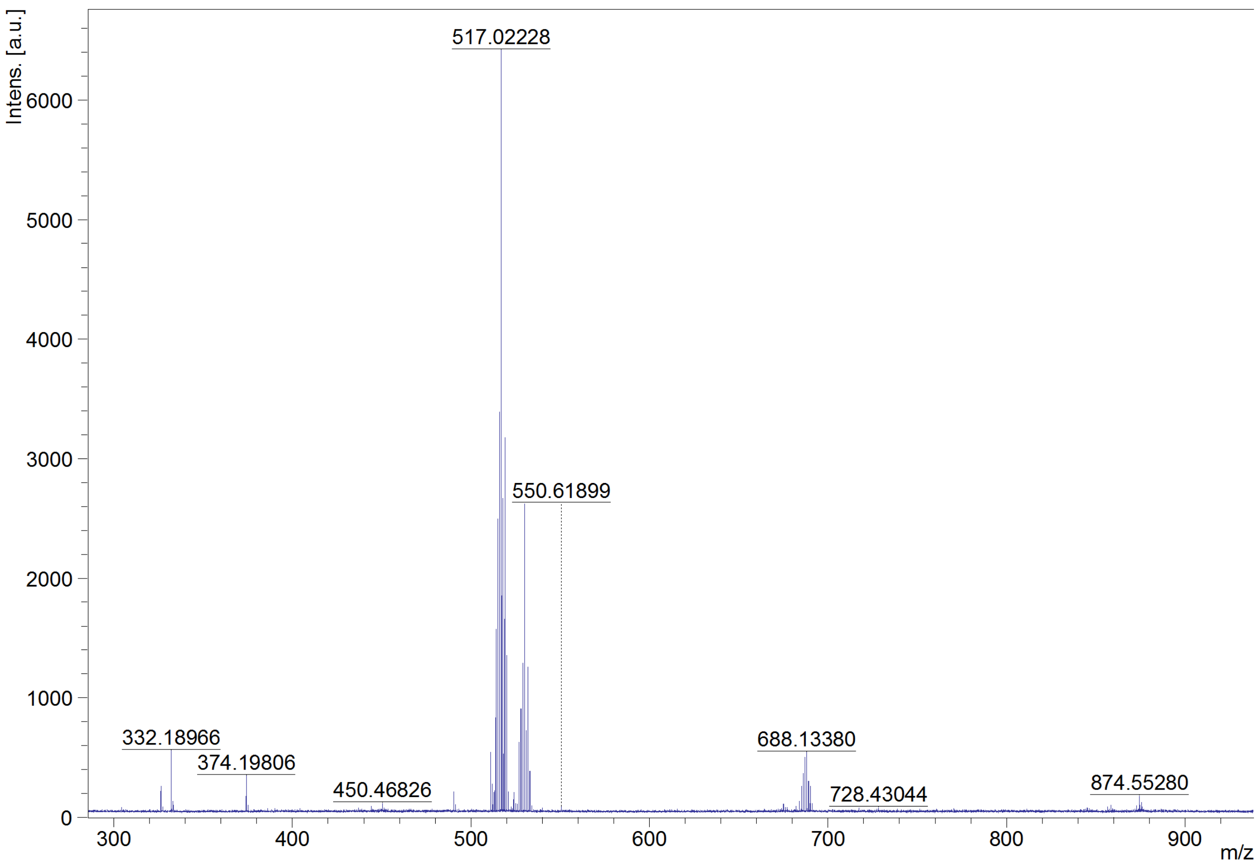

Figure S15. MALDI-TOF spectrum (DCTB, MeOH/$CHCl_3$, positive mode) of complex **S1**.

General procedure for the synthesis of Ru(bda) oligomers

End-cap **2**-*pic* or **2**-*dmap* (2.1 eq.) and bipyridine complex **1**-X (X = **A**, **B**, **C**, $\mathbf{C^{Me}}$, $\mathbf{C^{OMe}}$, 1.0 eq.) were dissolved in degassed TFE and heated for 19 hours (unless stated otherwise: to reflux in regular glassware with oil bath; alternatively at 100 °C in a microwave reactor). After cooling down to room temperature, the solvent was removed at reduced pressure and the residue was washed with copious amounts of MeOH, CH_2Cl_2, Et_2O and *n*-pentane in the given order. Afterwards, the remaining solid was collected. For quantification of by-product **1**-*pic*, the filtrate was evaporated and purified by column chromatography (Al_2O_3, 15% w/w H_2O, gradient DCM to DCM/MeOH 95:5).

pic[**Ru₄*B*₃**]*pic*

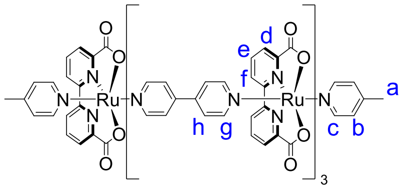

pic[**Ru_4*B_3***]*pic*
Chemical Formula: $C_{90}H_{62}N_{16}O_{16}Ru_4$
Molecular Weight: 2027.86

According to the general procedure, reaction of **2**-*pic* (33.2 mg, 64.5 µmol, 2.1 eq.) and **1-B** (20.0 mg, 30.5 µmol, 1.0 eq.) in degassed TFE (4 mL) yielded *pic*[**Ru_4*B_3***]*pic*.

Yield: 38.8 mg (19.1 µmol, 94%) of a dark red solid.

^{1}H-NMR (400 MHz, DCM-d_2/TFE 9:1, ascorbic acid): δ = 8.33-8.28 (m, 8H, H_f), 8.03-7.99 (m, 8H, H_d), 7.85-7.80 (m, 8H, H_e), 7.78-7.74 (m, 12H, H_g), 7.49 (d, ^3J = 6.2 Hz, 4H, H_c), 7.17-7.14 (m, 12H, H_h), 6.92 (d, ^3J = 6.2 Hz, 4H, H_b), 2.23 (s, 6H, H_a) ppm.

IR (ATR): ν = 668, 766, 1074, 1363, 1577, 2359, 2980 cm^{-1}.

M.p.: > 400 °C.

Anal. calc. for $C_{90}H_{62}N_{16}O_{16}Ru_4 \cdot (H_2O)_2$ (2527.31): C 52.38, H 3.22, N 10.86; found: C 52.17, H 2.97, N 11.04.

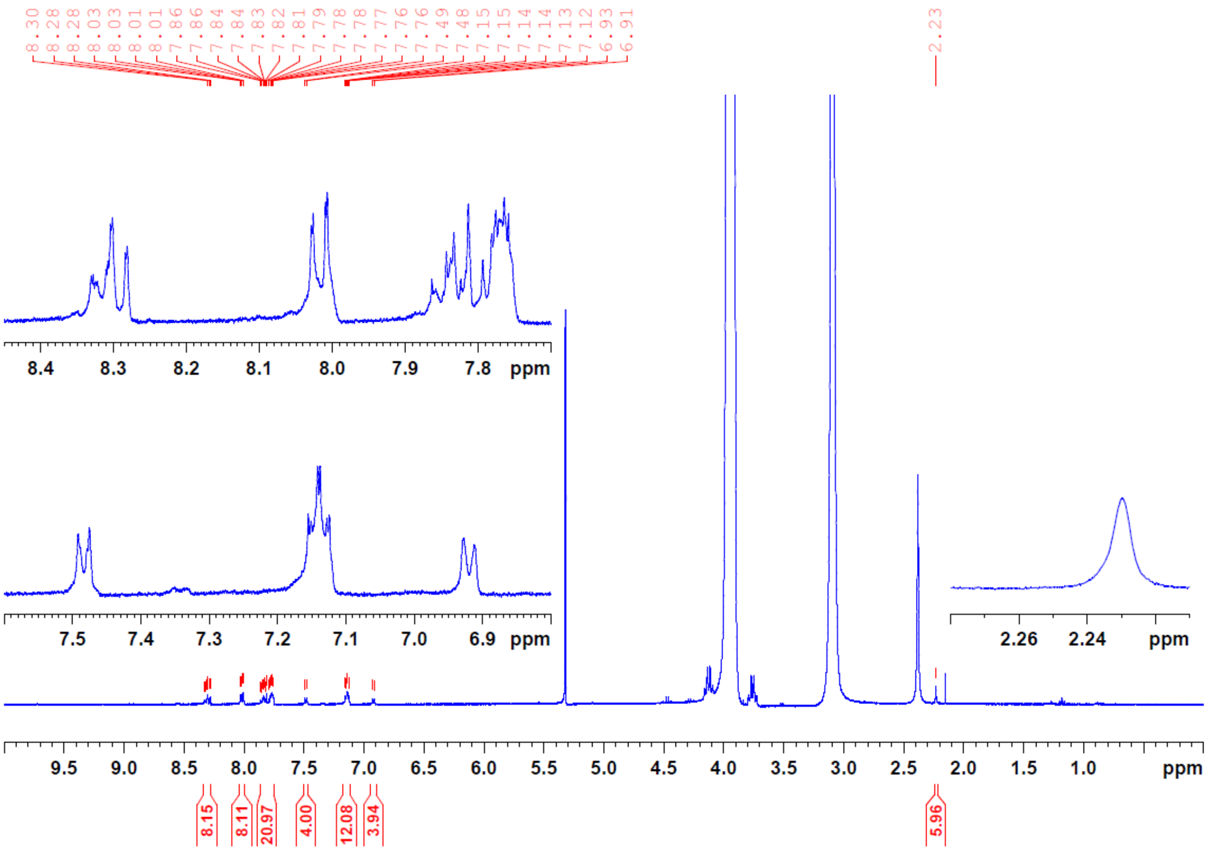

Figure S16. ^{1}H-NMR spectrum (400 MHz, DCM-d_2/TFE-d_3 9:1, ascorbic acid) of *pic*[**Ru_4*B_3***]*pic*.

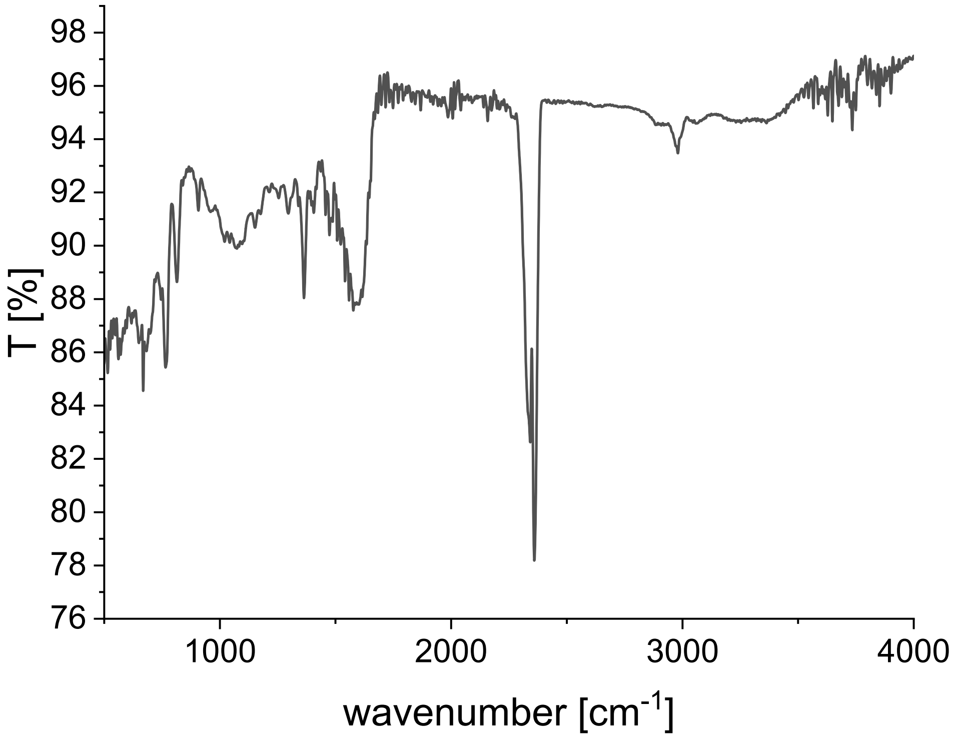

Figure S17. IR spectrum (ATR) of *pic*[**Ru$_4$*B*$_3$**]*pic*.

pic[**Ru$_5$*B*$_4$**]*pic*

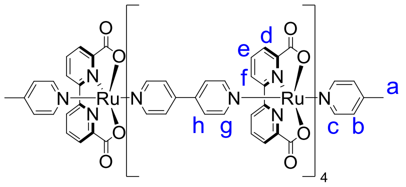

pic[**Ru$_5$*B*$_4$**]*pic*
Chemical Formula: $C_{112}H_{76}N_{20}O_{20}Ru_5$
Molecular Weight: 2527.31

According to the general procedure, the reaction of **1-B** (20.0 mg, 30.5 µmol, 1.0 eq.) with **2**-*pic* (33.2 mg, 64.5 µmol, 2.1 eq.) in degassed TFE (8 mL) yielded *pic*[**Ru$_5$*B*$_4$**]*pic*.

Yield: 37.1 mg (14.7 µmol, 96%) of a dark red solid.

^{1}H-NMR (400 MHz, DCM-d_2/TFE 9:1, ascorbic acid): δ = 8.33-8.28 (m, 10H, H_f), 8.03-7.99 (m, 10H, H_d), 7.85-7.80 (m, 10H, H_e), 7.78-7.74 (m, 16H, H_g), 7.49 (d, ^3J = 5.7 Hz, 4H, H_c), 7.17-7.14 (m, 16H, H_h), 6.92 (d, ^3J = 5.7 Hz, 4H, H_b), 2.23 (s, 6H, H_a) ppm.

IR (ATR): ν = 562, 668, 1507, 2358 cm^{-1}.

M.p.: > 400 °C.

Anal. calc. for $C_{112}H_{76}N_{20}O_{20}Ru_5{\cdot}(H_2O)_5{\cdot}(HOCH_2CF_3)_3$ (2917.51): C 48.58, H 3.28, N 9.60; found: C 48.26, H 3.63, N 9.84.

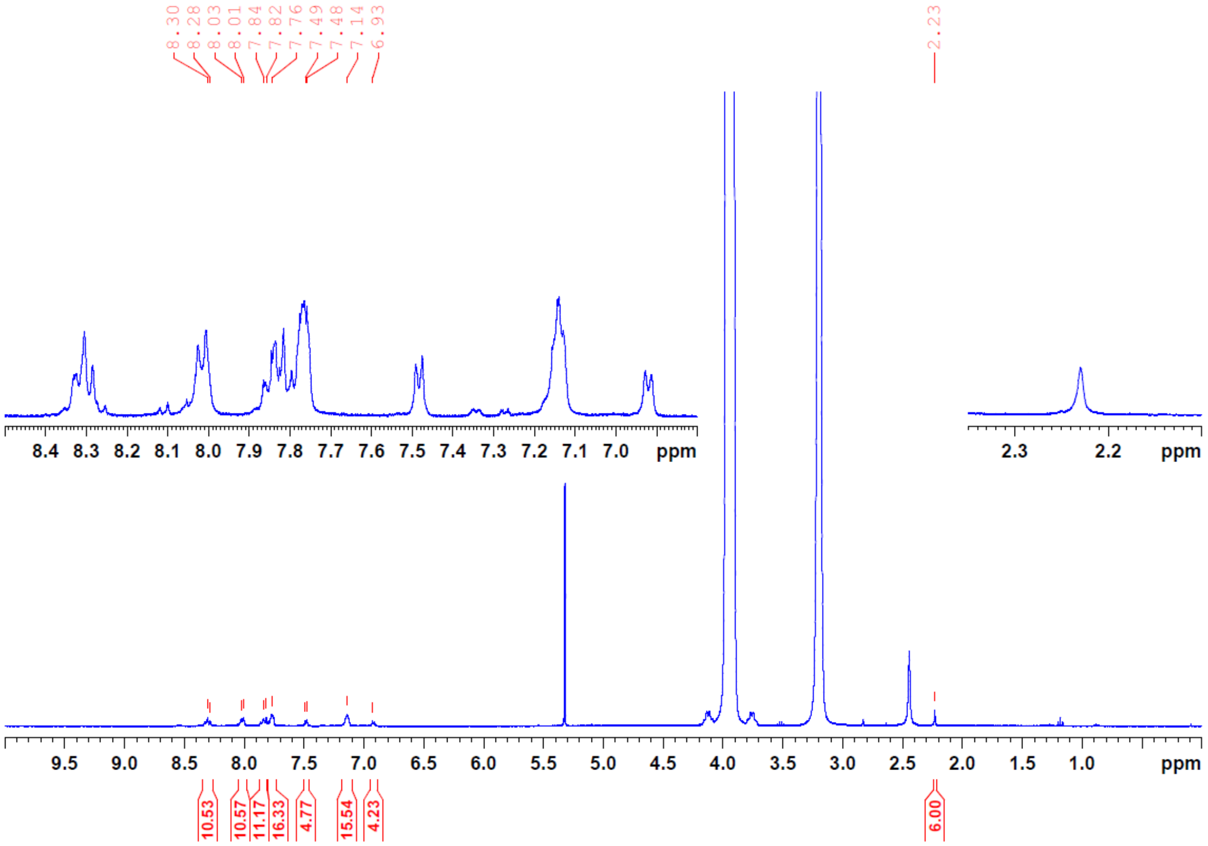

Figure S18. ^{1}H NMR spectrum (400 MHz, DCM-d_2/TFE-d_3 9:1, rt, ascorbic acid) of *pic*[**Ru$_5$*B*$_4$**]*pic*.

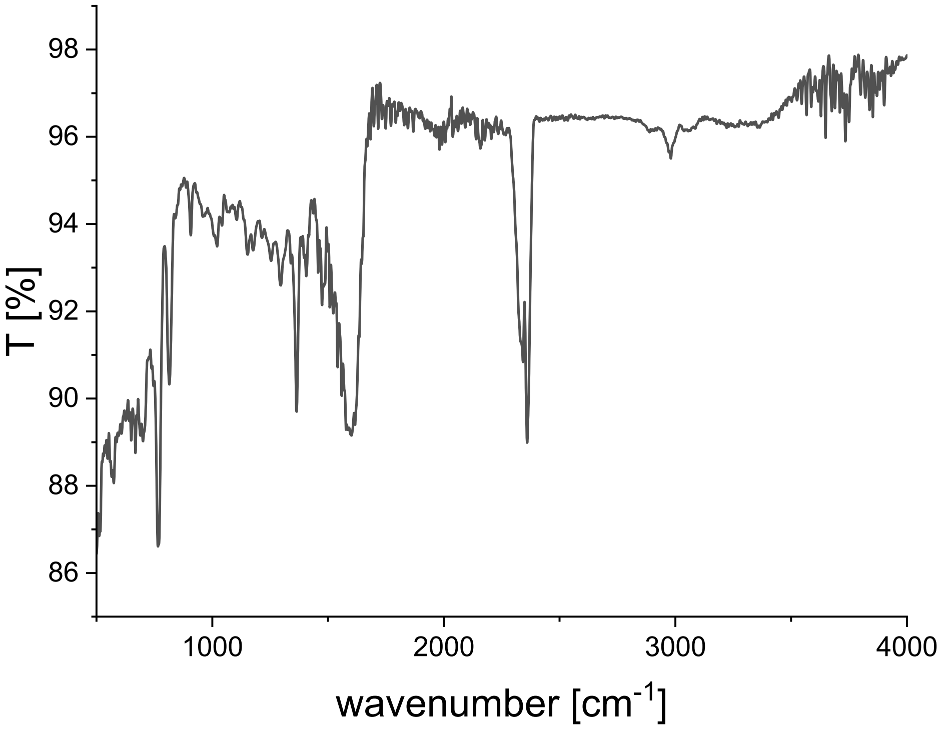

Figure S19. IR spectrum (ATR) of *pic*[**Ru$_5$*B*$_4$**]*pic*.

pic[**Ru₄*C*₃**]*pic*

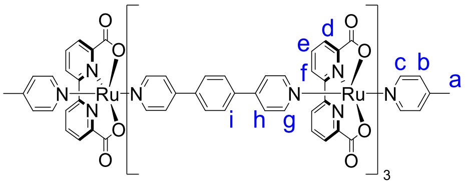

pic[**Ru_4C_3**]*pic*
Chemical Formula: $C_{108}H_{74}N_{16}O_{16}Ru_4$
Molecular Weight: 2256.16

According to the general procedure, the reaction of **1-C** (23.2 mg, 30.0 µmol, 1.0 eq.) with **2**-*pic* (33.2 mg, 64.5 µmol, 2.1 eq.) in degassed TFE (12 mL) yielded *pic*[**Ru_4*C*$_3$**]*pic*.

Yield: 35.9 mg (15.9 µmol, 74%) of a dark red solid.

¹H-NMR (400 MHz, DCM-d_2/TFE 9:1, ascorbic acid): δ = 8.34-8.28 (m, 8H, H_f), 8.08-8.03 (m, 8H, H_d), 7.86-7.80 (m, 8H, H_e), 7.76-7.71 (m, 12H, H_g), 7.57 (s, 12H, H_i), 7.52 (d, ^3J = 6.6 Hz, 4H, H_c), 7.33-7.29 (m, 12H, H_h), 6.93 (d, ^3J = 6.6 Hz, 4H, H_b), 2.24 (s, 6H, H_a) ppm.

IR (ATR): ν = 770, 1363, 1601, 2359, 3734 cm^{-1}.

M.p.: > 400 °C.

Anal. calc. for $C_{108}H_{74}N_{16}O_{16}Ru_4\cdot(H_2O)_4\cdot(HOCH_2CF_3)_4$ (2728.38): C 51.07, H 3.47, N 8.21; found: C 51.22, H 3.92, N 8.35.

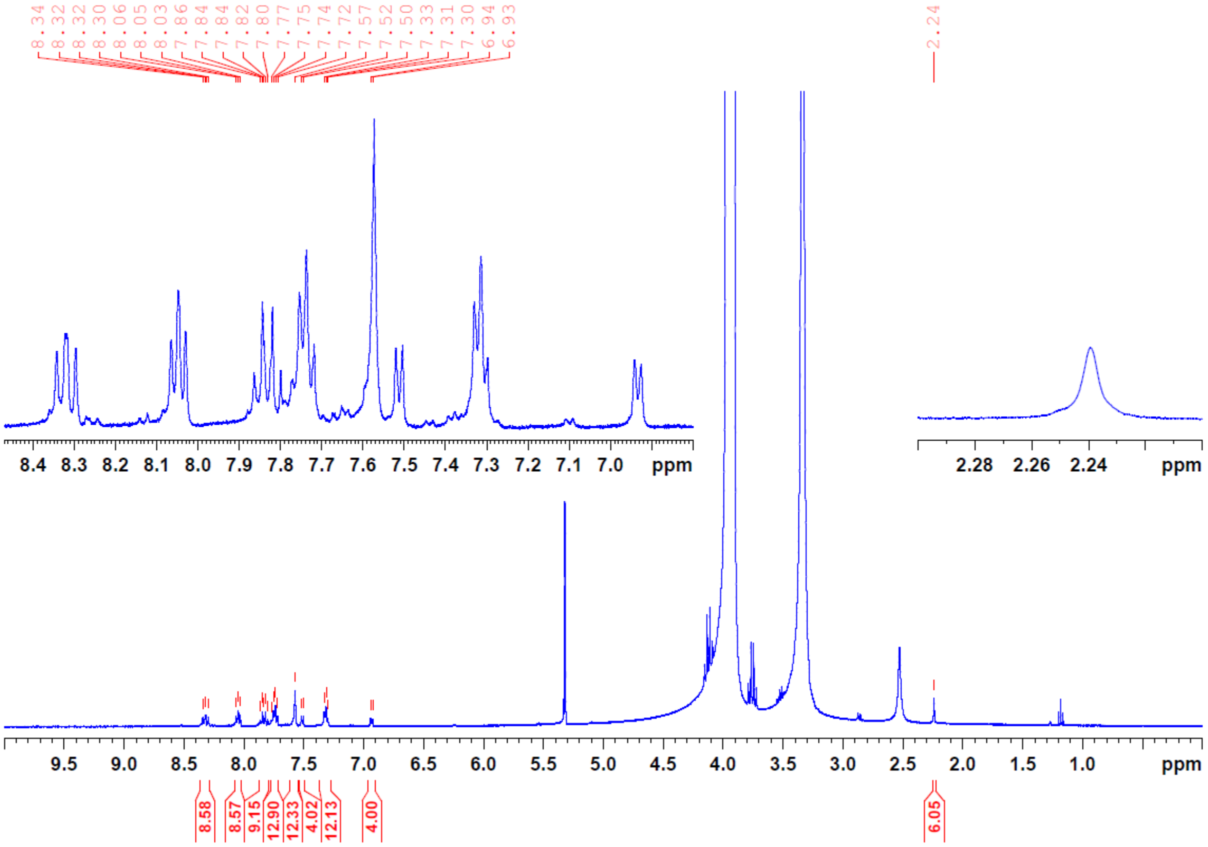

Figure S20. ¹H-NMR spectrum (400 MHz, DCM-d_2/TFE-d_3 9:1, rt, ascorbic acid) of *pic*[**Ru_4*C*$_3$**]*pic*.

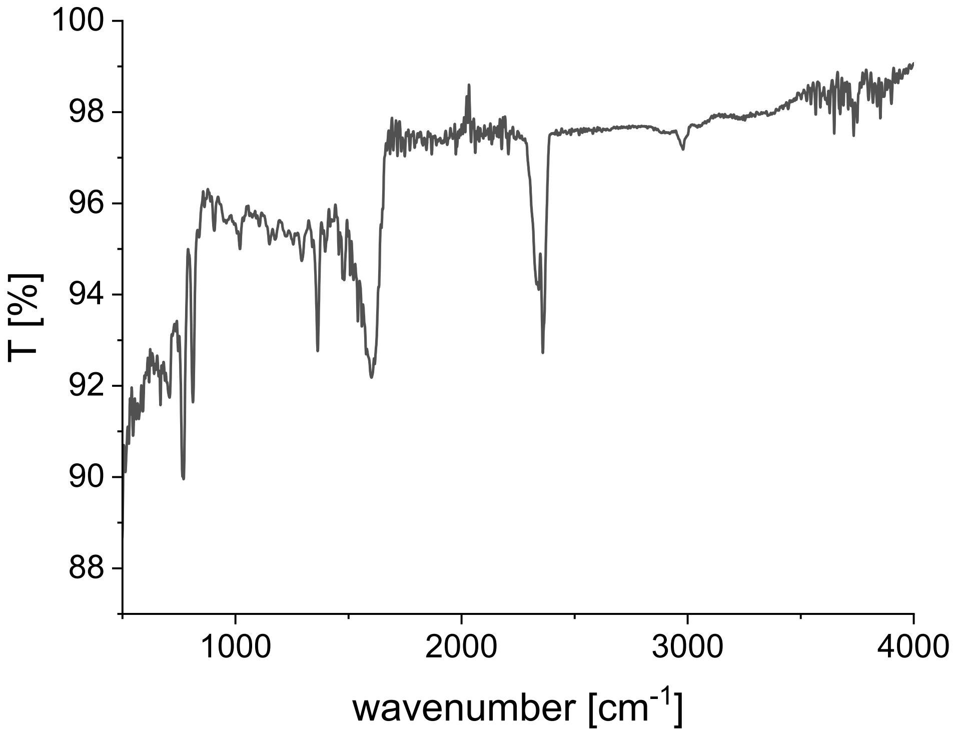

Figure S21. IR spectrum (ATR) of *pic*[**Ru₄*C*₃**]*pic*.

pic[$\mathbf{Ru_5}\boldsymbol{C_4}$]*pic*

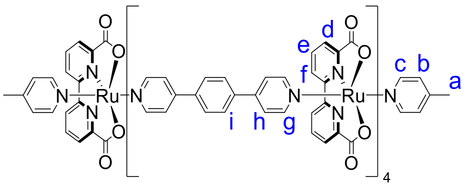

pic[$\mathbf{Ru_5}\boldsymbol{C_4}$]*pic*
Chemical Formula: $C_{136}H_{92}N_{20}O_{20}Ru_5$
Molecular Weight: 2831.70

According to the general procedure, the reaction of **1-C** (23.2 mg, 30.0 µmol, 1.0 eq.) with **2**-*pic* (33.2 mg, 64.5 µmol, 2.1 eq.) in degassed TFE (24 mL) yielded *pic*[$\mathbf{Ru_5}\boldsymbol{C_4}$]*pic*.

Yield: 41.5 mg (14.7 µmol, 93%) of a dark red solid.

^{1}H-NMR (400 MHz, DCM-d_2/TFE 9:1, ascorbic acid): δ = 8.36-8.28 (m, 10H, H_f), 8.08-8.02 (m, 10H, H_d), 7.88-7.81 (m, 10H, H_e), 7.76-7.71 (m, 16H, H_g), 7.57 (s, 16H, H_i), 7.51 (d, ^3J = 6.2 Hz, 4H, H_c), 7.35-7.28 (m, 16H, H_h), 6.93 (d, ^3J = 6.2 Hz, 4H, H_b), 2.24 (s, 6H, H_a) ppm.

IR (ATR): ν = 765, 1363, 1596, 2359, 2980, 3364 cm^{-1}.

M.p.: > 400 °C.

Anal. calc. for $C_{136}H_{92}N_{20}O_{20}Ru_5 \cdot (H_2O)_5 \cdot (HOCH_2CF_3)$ (3021.82): C 54.85, H 3.50, N 9.27; found: C 54.65, H 3.34, N 9.31.

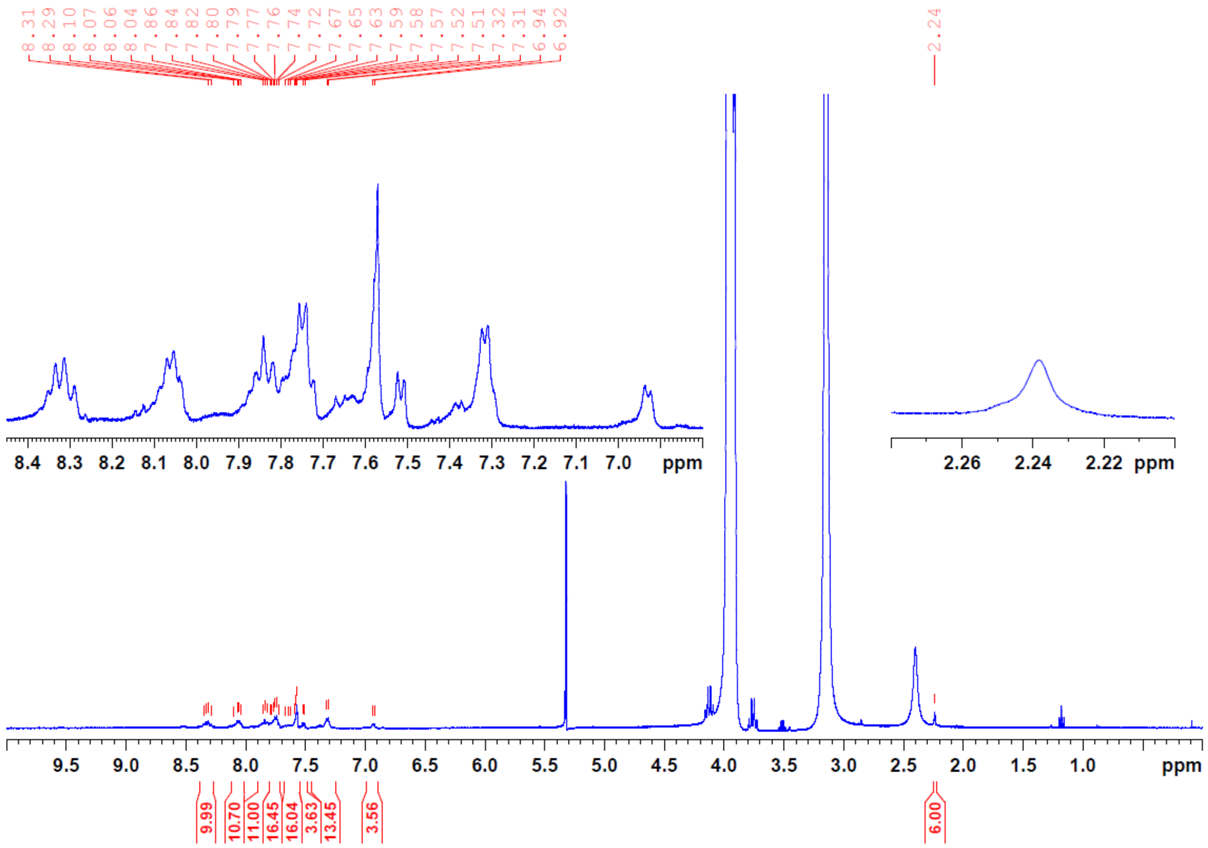

Figure S22. ^{1}H-NMR spectrum (400 MHz, DCM-d_2/TFE-d_3 9:1, rt, ascorbic acid) of *pic*[**Ru$_5$*C*$_4$**]*pic*.

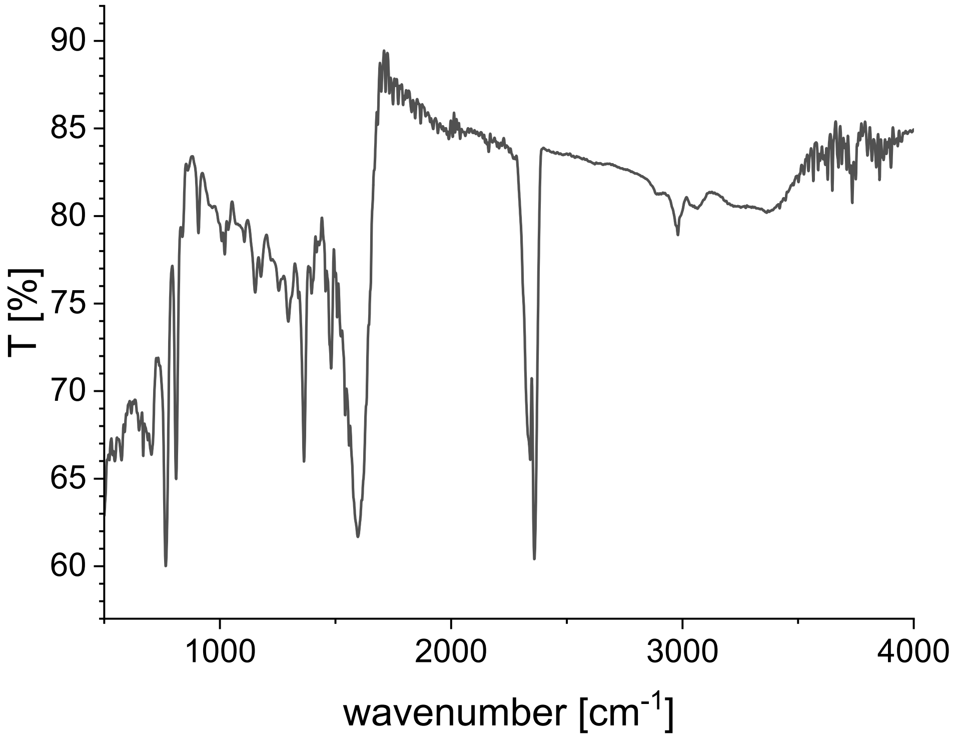

Figure S23. IR spectrum (ATR) of *pic*[**Ru$_5$*C*$_4$**]*pic*.

dmap[**Ru**$_4$***B***$_3$]*dmap*

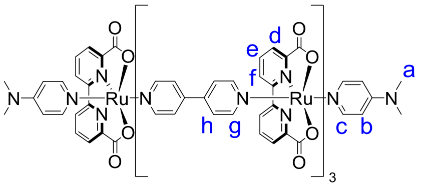

dmap[**Ru**$_4$***B***$_3$]*dmap*
Chemical Formula: $C_{92}H_{68}N_{18}O_{16}Ru_4$
Molecular Weight: 2085.95

According to the general procedure, reaction of **1-B** (7.90 mg, 12.0 µmol, 1.0 eq.) and **2**-*dmap* (13.7 mg, 25.2 µmol, 2.1 eq.) in TFE (6 mL) in a microwave reactor at 100 °C yielded *dmap*[**Ru**$_4$***B***$_3$]*dmap*.

Yield: 13.5 mg (6.50 µmol, 81%) of a dark red solid.

^{1}H-NMR (400 MHz, DCM-d_2/TFE 9:1, ascorbic acid): δ = 8.34-8.21 (m, 8H, H_f), 8.01 (d, 8H, H_d), 7.84 (t, 8H, H_e), 7.79-7.72 (m, 12H, H_g), 7.19-7.09 (m, 12H, H_h), 7.07 (d, 2H, H_c), 6.23 (d, 4H, H_b), 2.86 (s, 12H, H_a) ppm.

M.p.: 306 °C (decomp.).

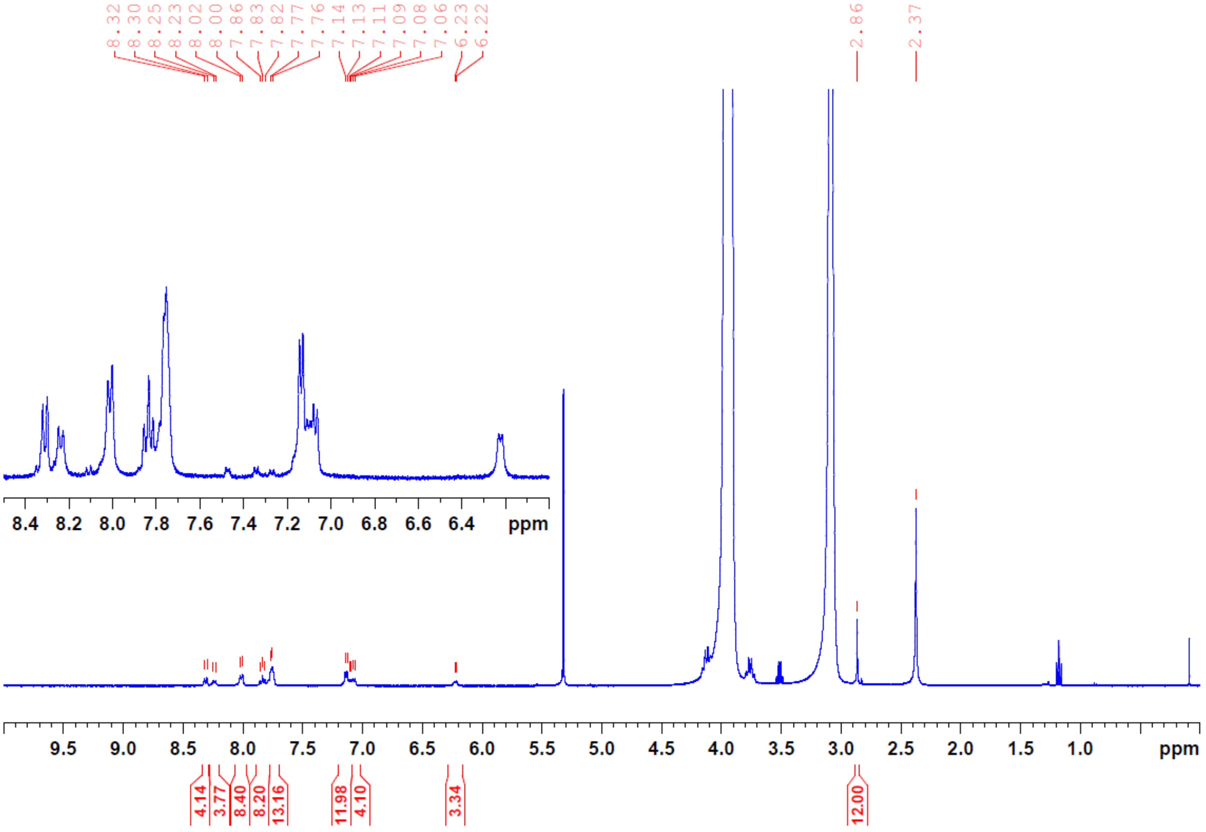

Figure S24. ^{1}H-NMR spectrum (400 MHz, CD_2Cl_2/TFE 9:1, rt, ascorbic acid) of *dmap*[**Ru**$_4$***B***$_3$]*dmap*.

pic[**Ru$_4$*C*^{Me}$_3$**]*pic*

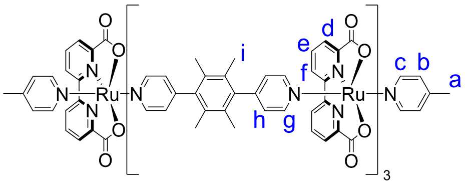

pic[**Ru$_4$C^{Me}$_3$**]*pic*
Chemical Formula: $C_{120}H_{98}N_{16}O_{16}Ru_4$
Molecular Weight: 2424.48

According to the general procedure, reaction of **1-C^{Me}** (6.90 mg, 7.50 µmol, 1.0 eq.) and **2**-*pic* (8.10 mg, 15.8 µmol, 2.1 eq.) in TFE (3 mL) in a microwave reactor at 100 °C yielded *pic*[**Ru$_4$*C*^{Me}$_3$**]*pic*.

Yield: 11.5 mg, 4.80 µmol, 96% of a dark red solid.

^{1}H-NMR (400 MHz, DCM-*d*$_2$/TFE 9:1, ascorbic acid): δ = 8.34-8.25 (m, 8H, H$_f$), 8.15-8.03 (m, 8H, H$_d$), 7.88-7.79 (m, 8H, H$_e$), 7.78-7.72 (m, 12H, H$_g$), 7.49 (d, 4H, H$_c$), 6.95-6.86 (m, 16H, H$_{b,h}$), 2.23 (s, 6H, H$_a$), 1.68 (s, 36H, H$_i$) ppm.

IR (ATR): v = 766, 1363, 1602, 2359, 2980, 3364 cm^{-1}.

M.p.: 317 °C (decomp.).

Anal. calc. for $C_{120}H_{98}N_{16}O_{16}Ru_4\cdot(H_2O)_4\cdot(HOCH_2CF_3)_3$ (2796.66): C 54.11, H 4.14, N 8.01; found: C 53.94, H 4.27, N 8.29.

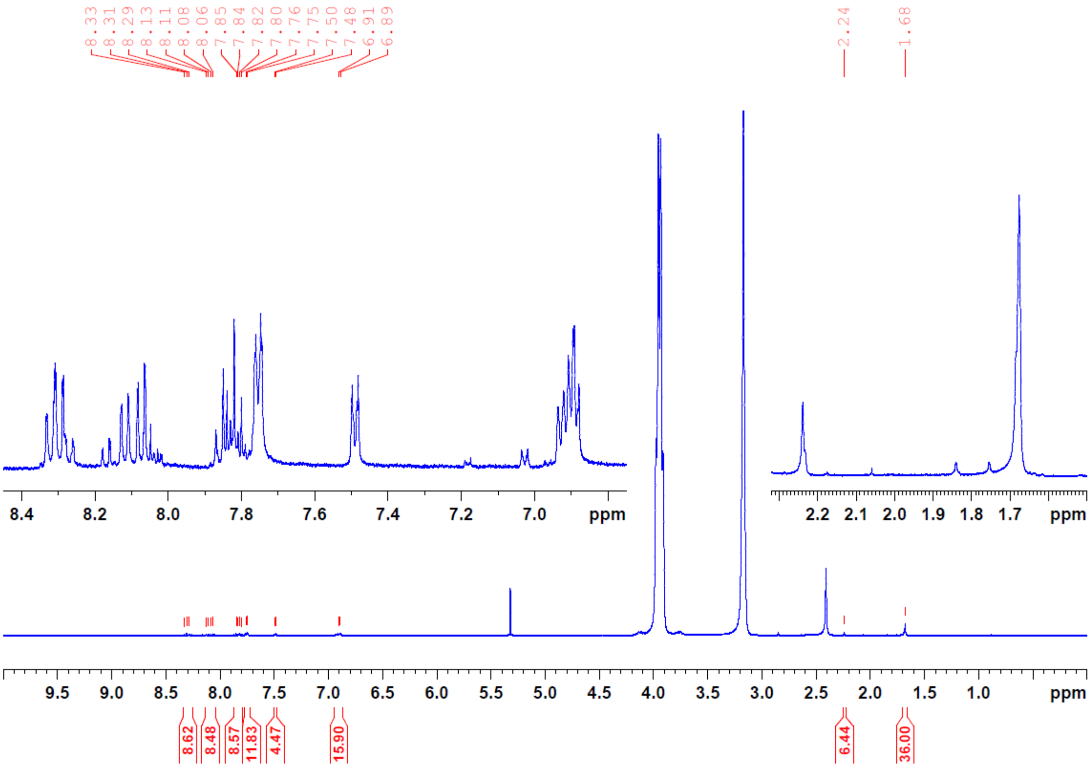

Figure S25. ^{1}H-NMR-spectrum (400 MHz, DCM-d$_2$/TFE-d$_3$ 9:1, rt, ascorbic acid) of *pic*[**Ru$_4$*C*^{Me}$_3$**]*pic*.

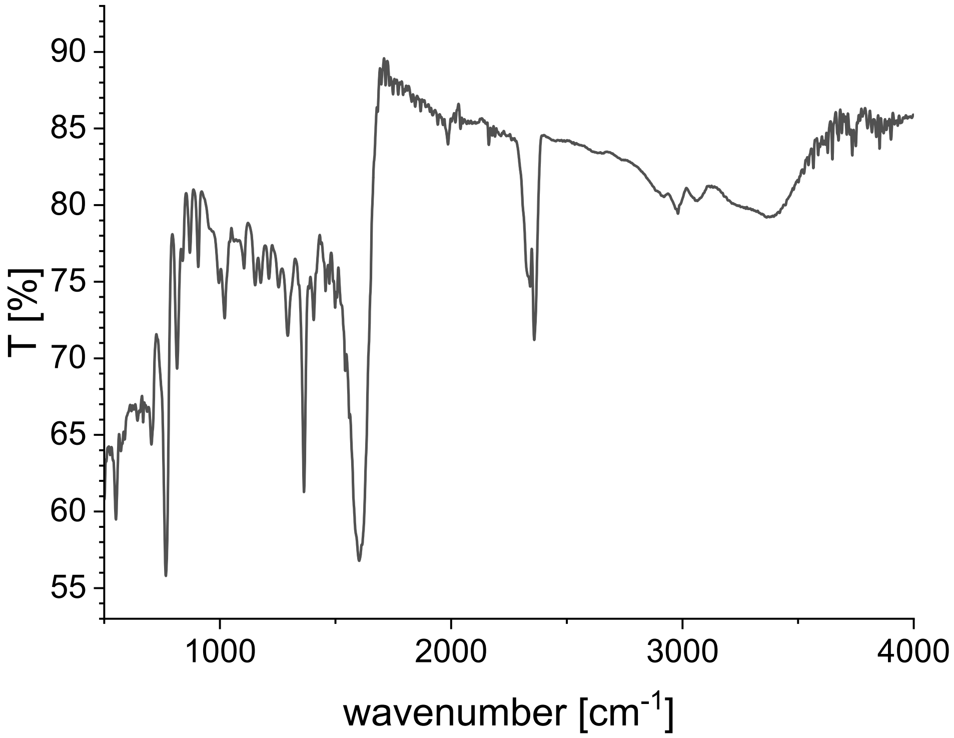

Figure S26. IR spectrum (ATR) of *pic*[**Ru$_4$*C^{Me}*$_3$**]*pic*.

pic[**Ru$_5$*C^{Me}*$_4$**]*pic*

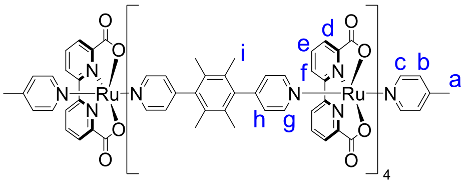

pic[**Ru$_5$*C^{Me}*$_4$**]*pic*
Chemical Formula: $C_{152}H_{124}N_{20}O_{20}Ru_5$
Molecular Weight: 3056.13

According to the general procedure, reaction of **1-C^{Me}** (13.8 mg, 15.0 µmol, 1.0 eq.) and **2**-*pic* (16.2 mg, 31.5 µmol, 2.1 eq.) in TFE (12 mL) yielded *pic*[**Ru$_5$*C^{Me}*$_4$**]*pic*.

Yield: 17.8 mg, 5.80 µmol, 74% of a dark red solid.

^{1}H-NMR (400 MHz, DCM-*d*$_2$/TFE 9:1, ascorbic acid): δ = 8.35-8.26 (m, 10H, H$_f$), 8.15-8.05 (m, 10H, H$_d$), 7.88-7.81 (m, 10H, H$_e$), 7.80-7.73 (m, 16H, H$_g$), 7.50 (d, 4H, H$_c$), 6.97-6.86 (m, 20H, H$_{b,h}$), 2.23 (s, 6H, H$_a$), 1.68 (s, 48H, H$_i$) ppm.

M.p.: 331 °C (decomp.).

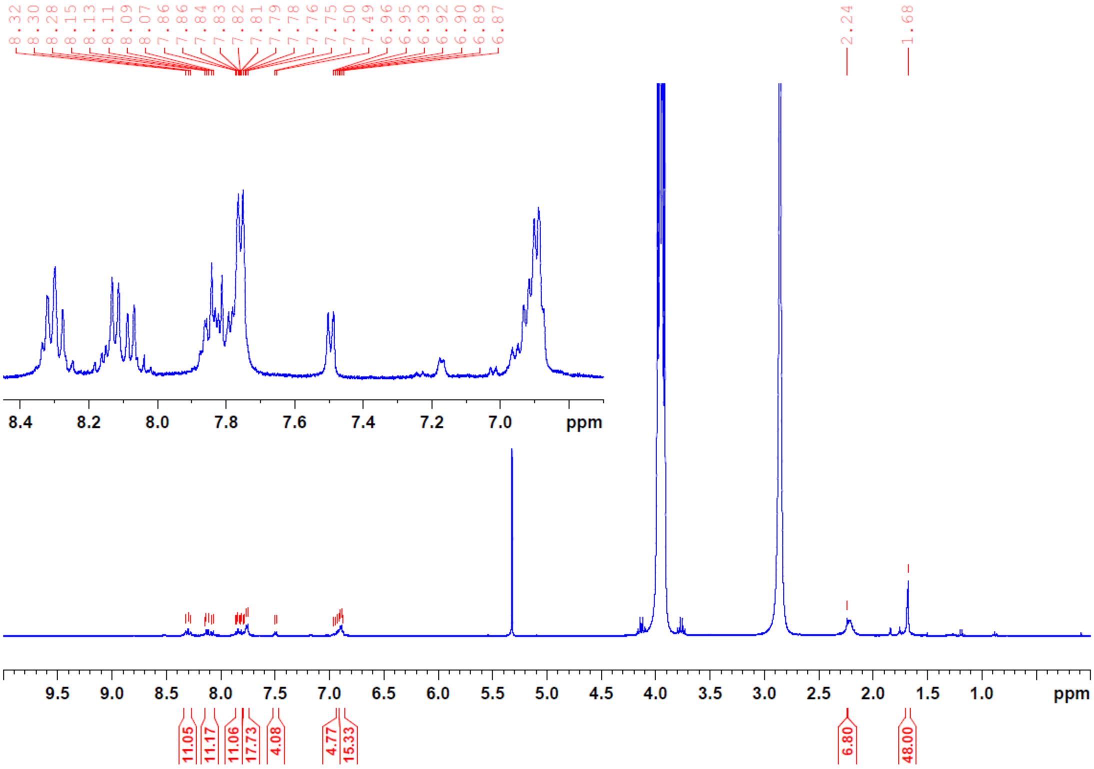

Figure S27. ^{1}H-NMR spectrum (400 MHz, DCM-d_2/TFE-d_3 9:1, rt ascorbic acid) of *pic*[**Ru$_5$*C*^{Me}$_4$**]*pic*.

pic[**Ru$_4$*C*^{OMe}$_3$**]*pic*

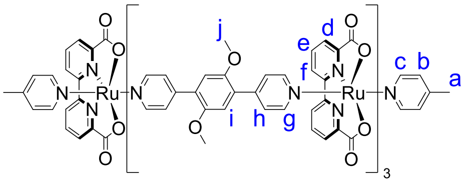

pic[**Ru$_4$*C*^{OMe}$_3$**]*pic*
Chemical Formula: $C_{114}H_{86}N_{16}O_{22}Ru_4$
Molecular Weight: 2436.31

According to the general procedure, reaction of **1-C^{OMe}** (13.9 mg, 15.0 µmol, 1.0 eq.) and **2**-*pic* (16.2 mg, 31.5 µmol, 2.1 eq.) in TFE (6 mL) yielded *pic*[**Ru$_4$*C*^{OMe}$_3$**]*pic*.

Yield: 23.3 mg, 9.60 µmol, 91% of a dark red solid.

^{1}H-NMR (400 MHz, DCM-*d*$_2$/TFE 9:1, ascorbic acid): δ = 8.31 (m, 8H, H$_f$), 8.06 (t, 8H, H$_d$), 7.83 (m, 8H, H$_e$), 7.76 (m, 12H, H$_g$), 7.51 (d, 4H, H$_c$), 7.31 (m, 12H, H$_h$), 6.93 (d, 4H, H$_b$), 6.82-6.77 (m, 6H, H$_i$), 3.65-3.63 (m, 18H, H$_j$), 2.23 (s, 6H, H$_a$) ppm.

IR (ATR): v = 538, 695, 832, 1209, 1482, 1598, 2359, 2980 cm^{-1}.

M.p.: 333 °C (decomp.).

Anal. calc. for $C_{114}H_{86}N_{16}O_{22}Ru_4\cdot(H_2O)_4\cdot(HOCH_2CF_3)_3$ (2808.49): C 51.32, H 3.70, N 7.98; found: C 51.30, H 3.77, N 8.54.

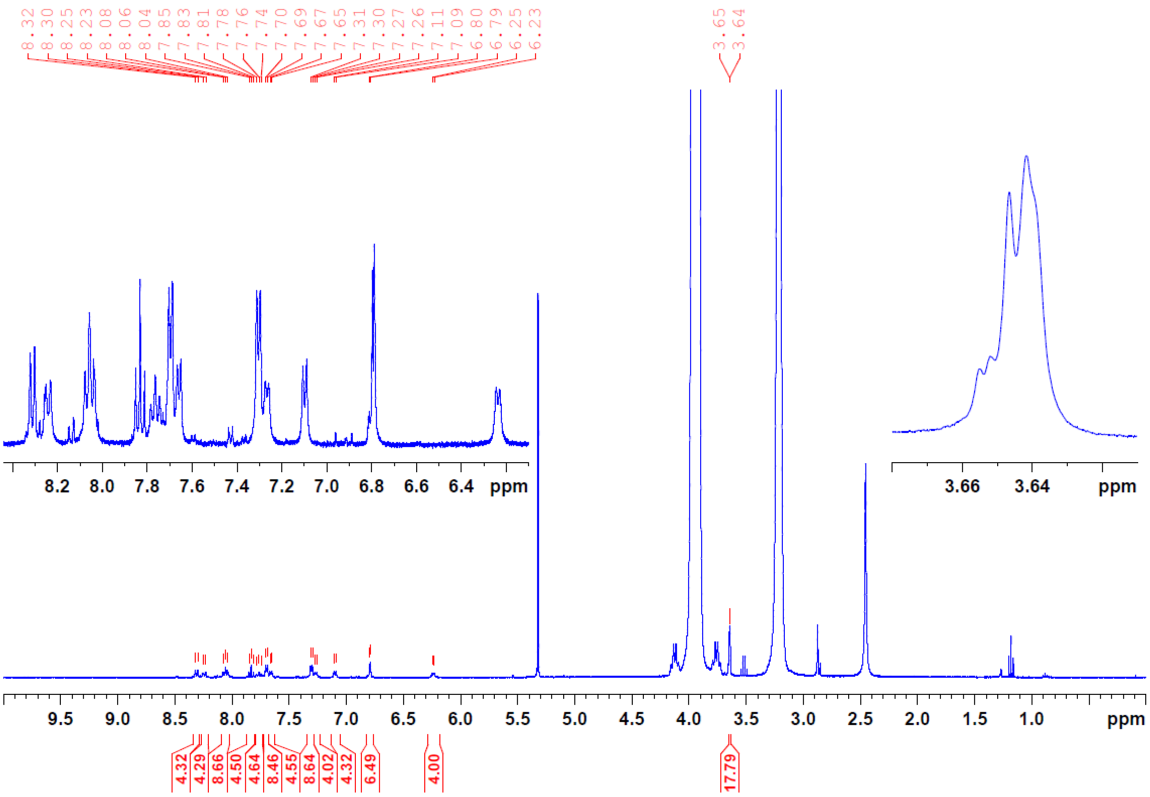

Figure S28. ^{1}H-NMR spectrum (400 MHz, DCM-d$_2$/trifluoroethanol 9:1, rt, ascorbic acid) of *pic*[**Ru$_5$*C*^{Me}$_4$**]*pic*.

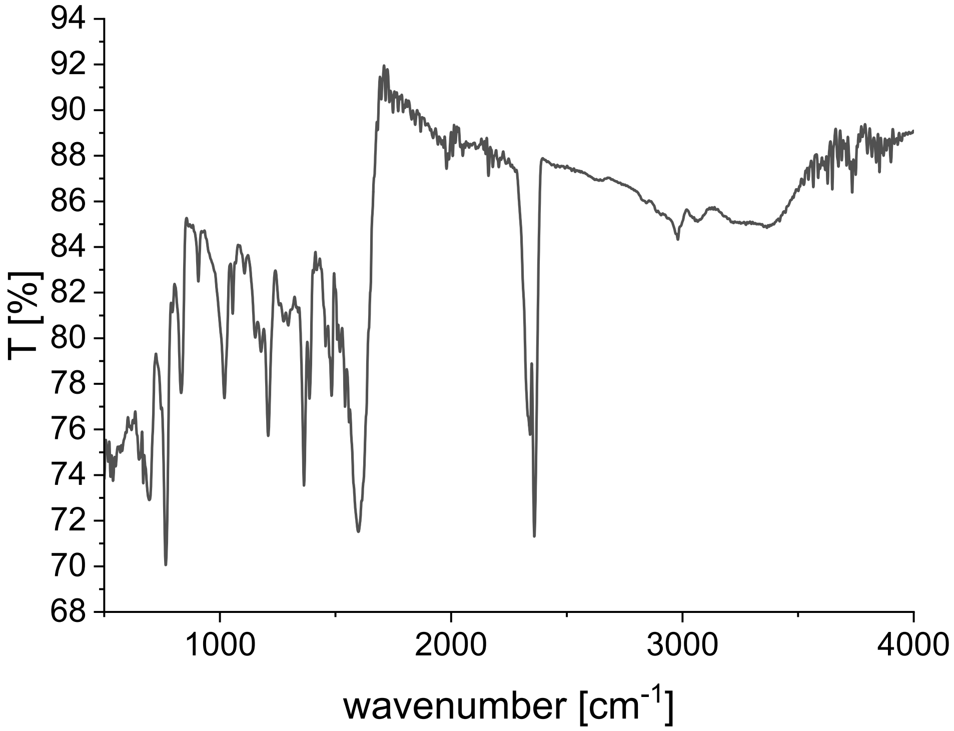

Figure S29. IR spectrum (ATR) of *pic*[**Ru$_4$*C*^{OMe}$_3$**]*pic*.

mixture of Ru(bda)-pyrazine oligomers

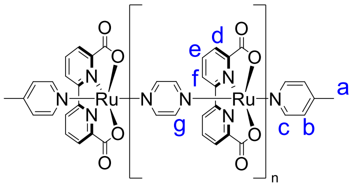

pic[**Ru$_n$*A*$_{n-1}$**]*pic*

According to the general procedure, **1-A** (20.0 mg, 39.7 µmol, 1.0 eq.) and **2**-*pic* (42.9 mg, 83.4 µmol, 2.1 eq.) were reacted in TFE (6 mL) in a microwave reactor at 100 °C. ^{1}H NMR analysis indicated a complex mixture of oligomers rather than one pure compound.

Yield: 29.0 mg of a dark red solid.

^{1}H-NMR (400 MHz, DCM-*d*$_2$/TFE 9:1, ascorbic acid): δ = 8.35-8.26 (m, 6H), 8.03-7.83 (m, 12H), 7.53-7.50 (m, 2H), 7.48 (s, 2H), 7.43-7.31 (m, 8H), 6.91-6.89 (m, 4H), 2.23-2.19 (m, 6H) ppm.

IR (ATR): ν = 766, 815, 1364, 1617, 2359, 2980, 3364 cm^{-1}.

M.p.: > 400 °C.

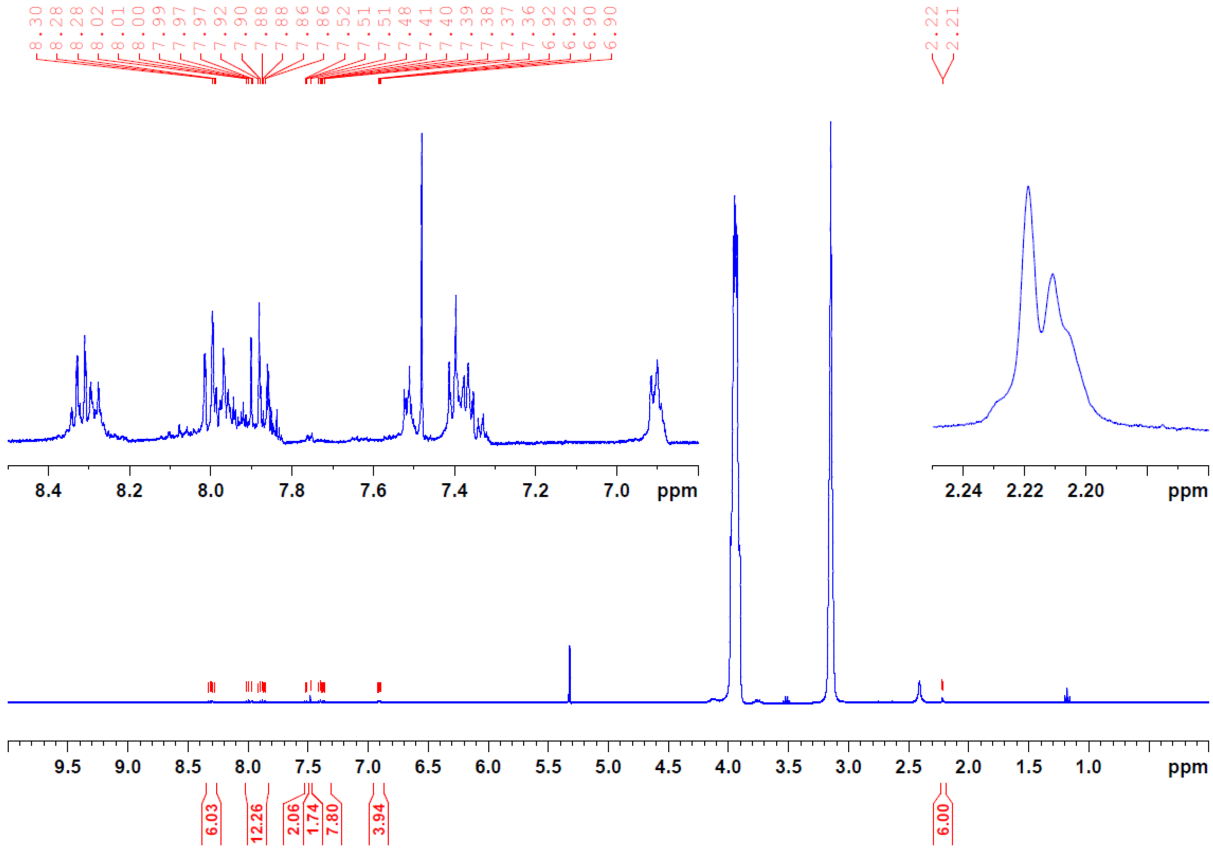

Figure S30. ^{1}H-NMR spectrum (400 MHz, DCM-d$_2$/TFE-d$_3$ 9:1, rt, ascorbic acid) of complex mixture of Ru(bda)-pyrazine oligomers.

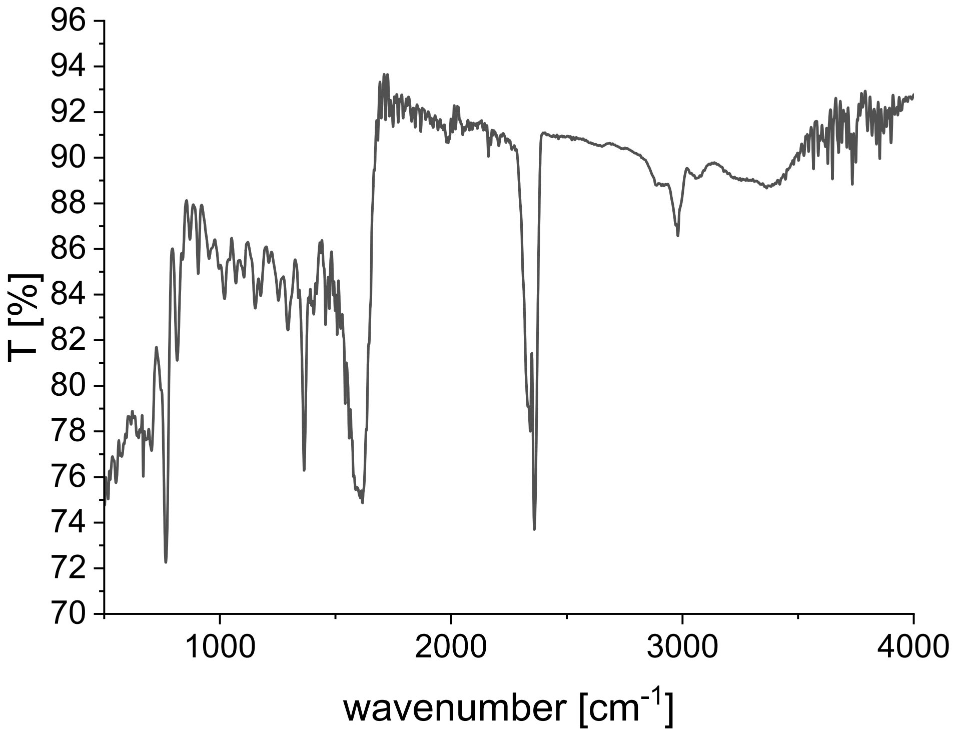

Figure S31. IR spectrum (ATR) of *pic*[$\mathbf{Ru_n}\boldsymbol{A}_{\mathbf{n-1}}$]*pic*.

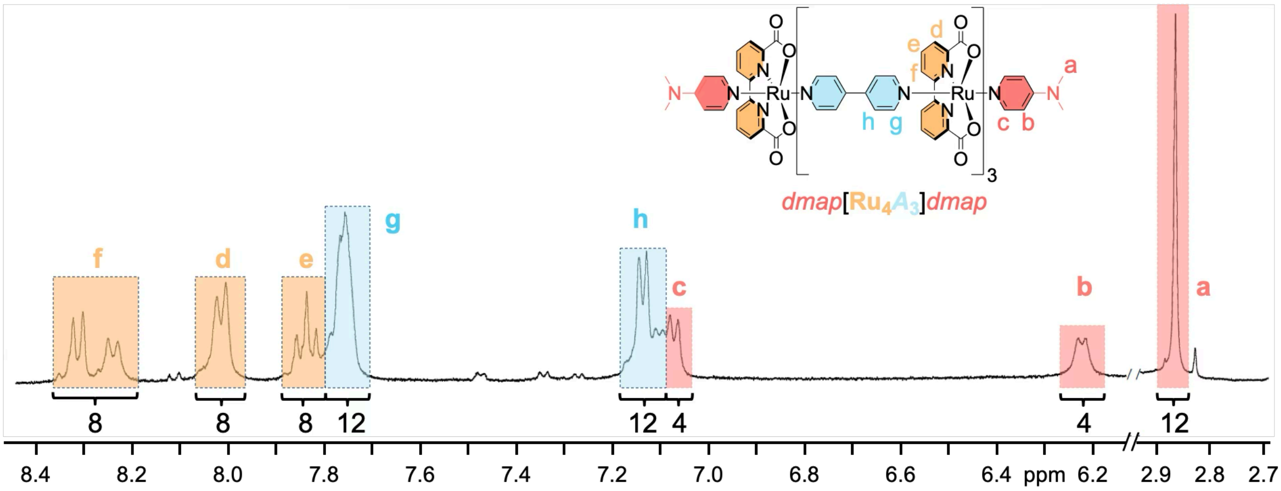

Figure S32. Size determination of Ru(bda) oligomers based on ligand **B** and end-cap *dmap* via end group analysis using ^{1}H-NMR spectroscopy (400 MHz, CD_2Cl_2/TFE-d_3 9:1, rt, ascorbic acid). NMR signals for the different components *dmap* (light red), bda (yellow), **B** (light blue) are highlighted in matching colors.

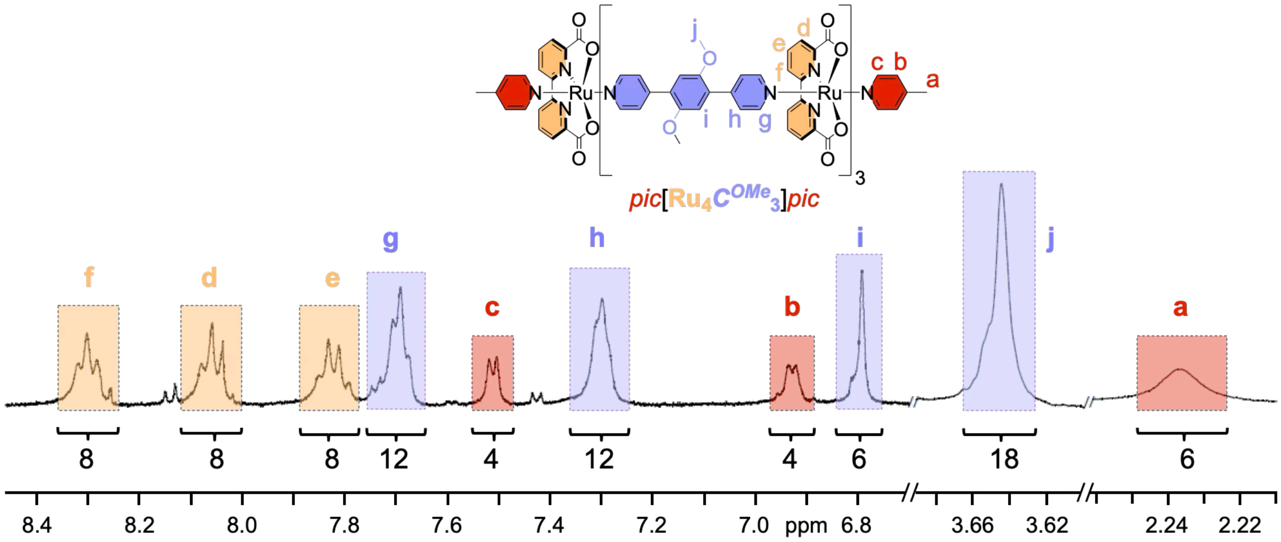

Figure S33. Size determination of Ru(bda) oligomers based on ligand $\mathbf{C^{OMe}}$ and end-cap *pic* via end group analysis using ^{1}H-NMR spectroscopy (400 MHz, CD_2Cl_2/TFE-d_3 9:1, rt, ascorbic acid). NMR signals for the different components *pic* (red), bda (yellow), and $\mathbf{C^{OMe}}$ (violet) are highlighted in matching colors.

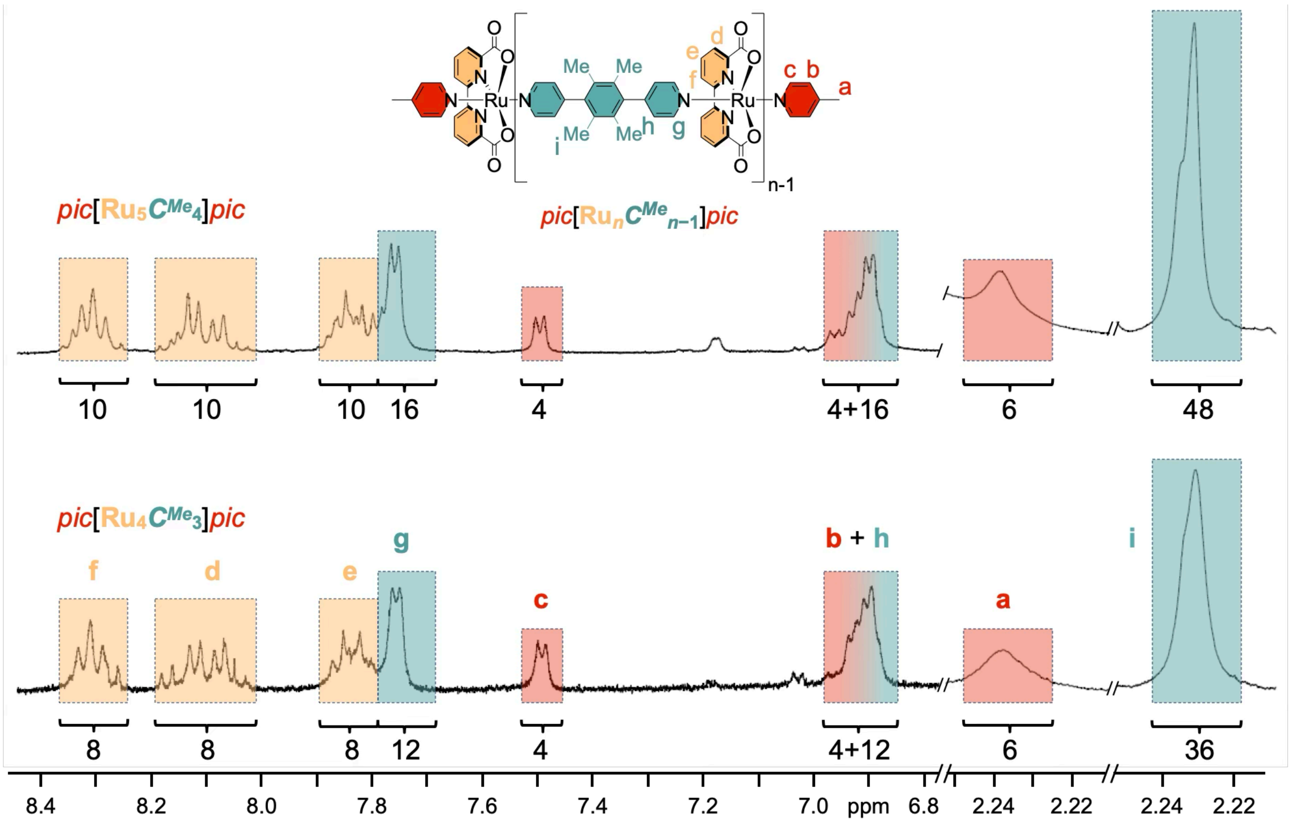

Figure S34. Size determination of Ru(bda) oligomers based on ligand $\mathbf{C^{Me}}$ and end-cap *pic* via end group analysis using ^{1}H-NMR spectroscopy (400 MHz, CD_2Cl_2/TFE-d_3 9:1, rt, ascorbic acid). NMR signals for the different components *pic* (red), bda (yellow), and $\mathbf{C^{Me}}$ (turquoise) are highlighted in matching colors.

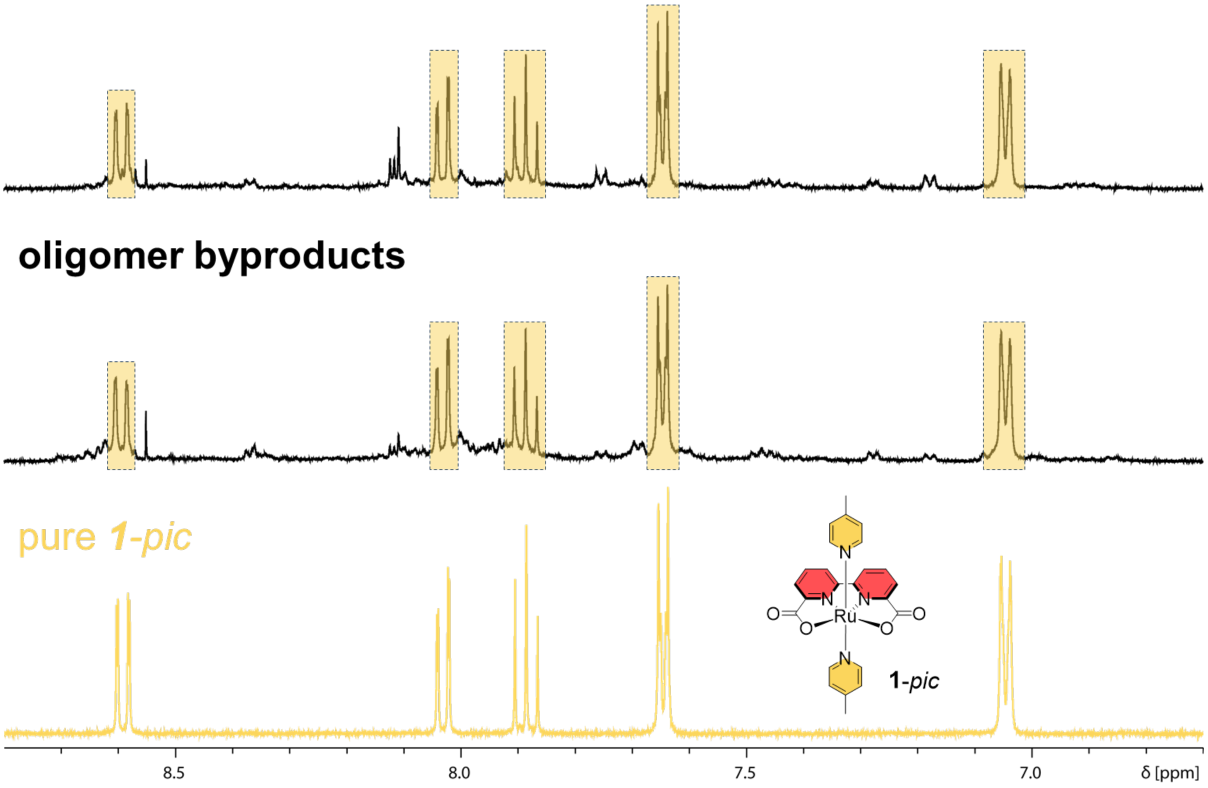

Figure S35. ^{1}H-NMR spectra (CD_3OD, 400 MHz, rt) of pure **1**-*pic* (bottom) and the soluble fractions obtained during workup for two different batches of *pic*[**Ru$_4$*B*$_3$**]*pic* (middle and top) wherein signals originating from **1**-*pic* are highlighted in yellow; for clarity, only the aromatic region of the spectra is shown.

Table S1. Exemplary calculation of the conversion of all precursors into *pic*[**Ru$_5$*B*$_4$**]*pic* and **1**-*pic*.

**component**	**starting material**	*pic*[**Ru$_5$*B*$_4$**]*pic* 14.7 µmol[b]	**1**-*pic* 14.5 µmol[b]	**loss**[d]
**Ru(bda)**	95.0 µmol[a]	73.5/79.2 µmol, 93%[c]	14.5/15.8 mmol, 92%[c]	7.4 µmol, 7%
**B**	61.0 µmol[a]	58.8/61.0 µmol, **96%**[c]	-	3.4 µmol, 4%
**pic**	64.5 µmol[a]	29.4/32.3 µmol, 91%[c]	29.0/32.3 µmol, 90%[c]	9.4 µmol, 9%

[a] used amount; [b] isolated amount; [c] before slash: isolated in form of the respective compound; after slash: theoretical maximum. [d]: amount that was not found in either isolated compound; percentage calculated based on total amount of starting material.

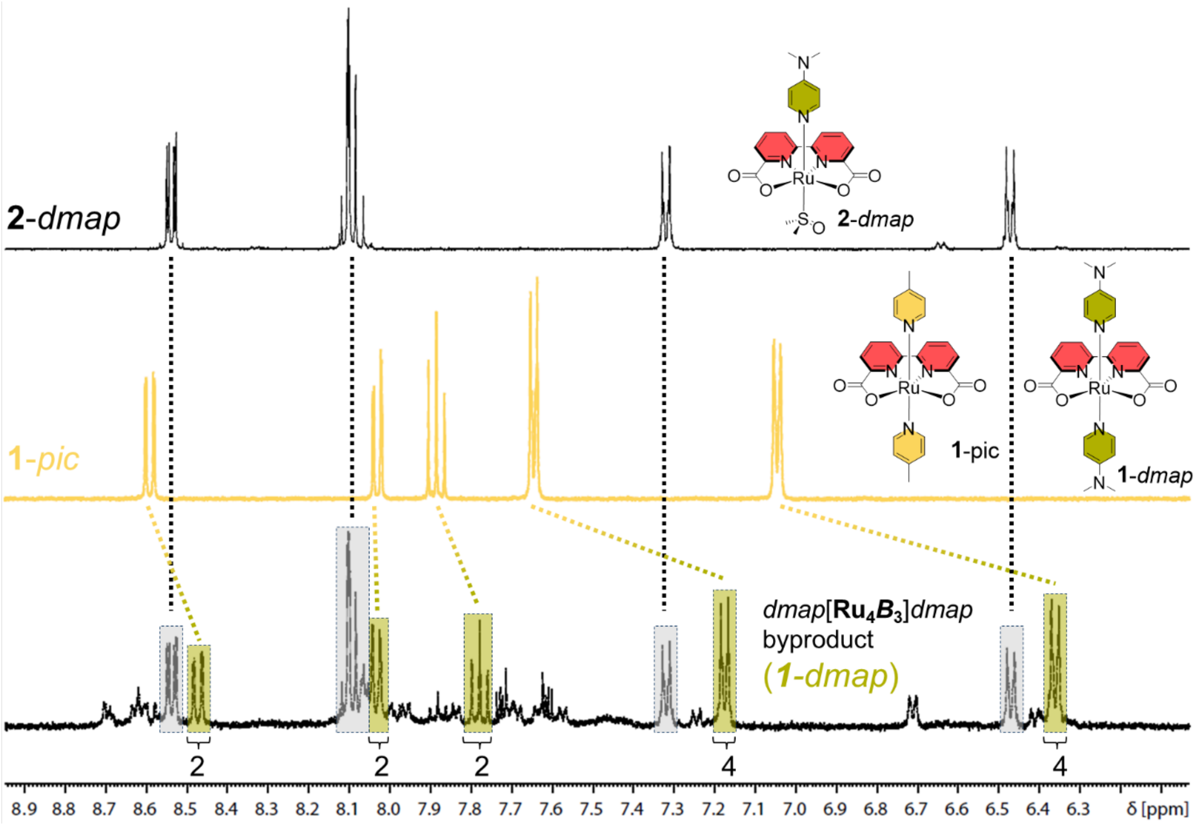

Figure S36. ^{1}H-NMR spectra (conditions: 400 MHz, methanol-d4) of **2**-*dmap* (top, black), **1**-*pic* (middle, gold; only for comparison) and the soluble byproduct mixture obtained during the synthesis of *dmap*[$\mathbf{Ru_4}\boldsymbol{B_3}$]*dmap* (bottom, black); signals originating from residual **2**-*dmap* are marked in grey, while signals of **1**-*dmap* are integrated and highlighted brown; for clarity, only the aromatic region of all spectra is shown.

4 Screening of Reaction Conditions

Table S2. Experimental observations for various axial ligand exchange experiments. The starting materials were dissolved in the respective solvent and heated to reflux overnight.

entry	reactants	solvent	
		MeOH	TFE
1	**1-B** + **C** (200 eq.)	**no conversion**	**no conversion**
2	**1-C** + **B** (200 eq.)	**no conversion**	**no conversion**
3	*pic*[**Ru$_4$*C*$_3$**]*pic* + **B** (200 eq.)	–[a]	*dissolution of oligomer*
4	*pic*[**Ru$_5$*C*$_4$**]*pic* + **1-B** (20 eq.)	–[a]	*mostly uncapped oligomer with both linkers included*
5	*pic*[**Ru$_5$*B*$_4$**]*pic* (control)	–[a]	**oligomer stable**

[a] reaction has not been carried out due to insufficient solubility of the starting materials in MeOH

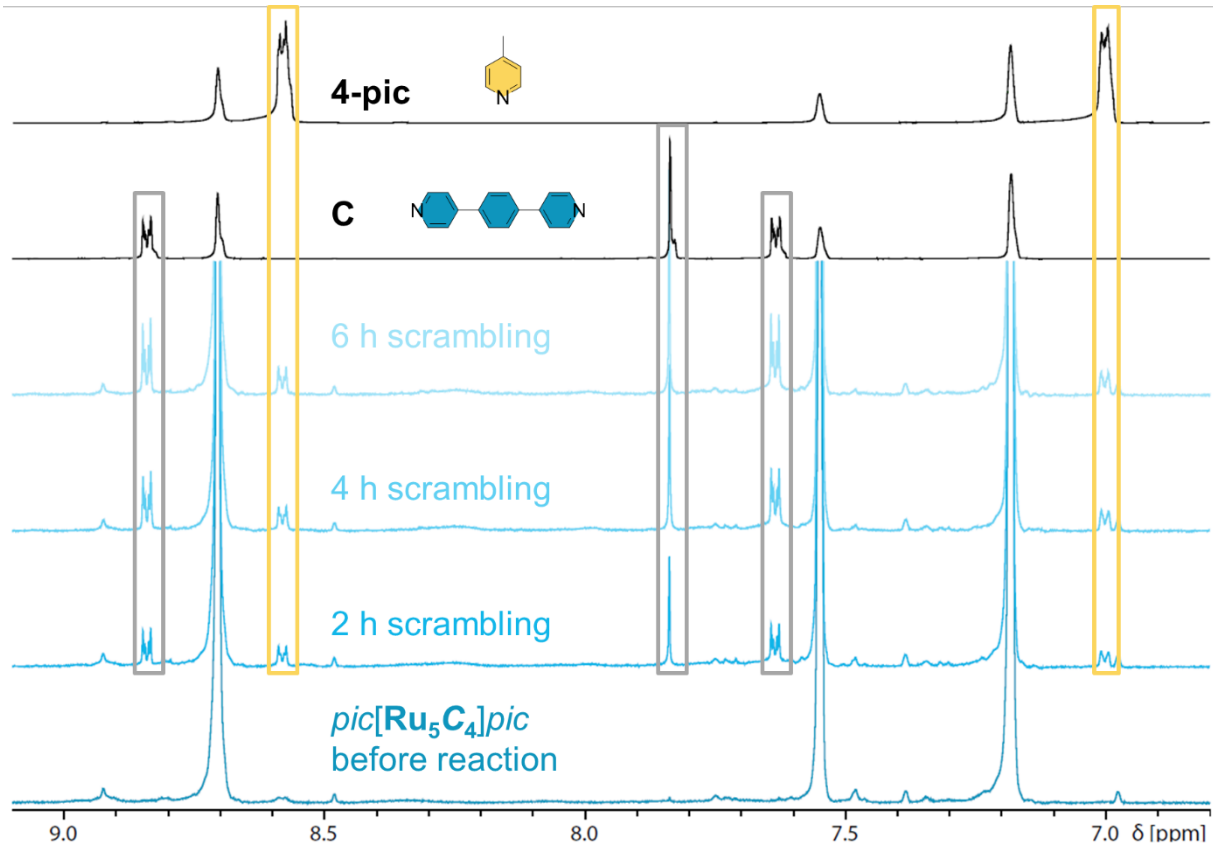

Figure S37. ^{1}H NMR monitoring (400 MHz, pyridine-d_5) of the aromatic region for scrambling experiment of *pic*[**Ru$_5$*C*$_4$**]*pic* in coordinating solvent pyridine: *pic*[**Ru$_5$*C*$_4$**]*pic* (bottom); heating to reflux for 2–6 h (blue hues) and free building blocks **C** and *pic* for comparison (black); arising signals for free **C** and *pic* are highlighted in grey and yellow, respectively; due to poor solubility of the oligomer in pyridine-d_5, signals of the intact oligomer were not observed during this experiment.

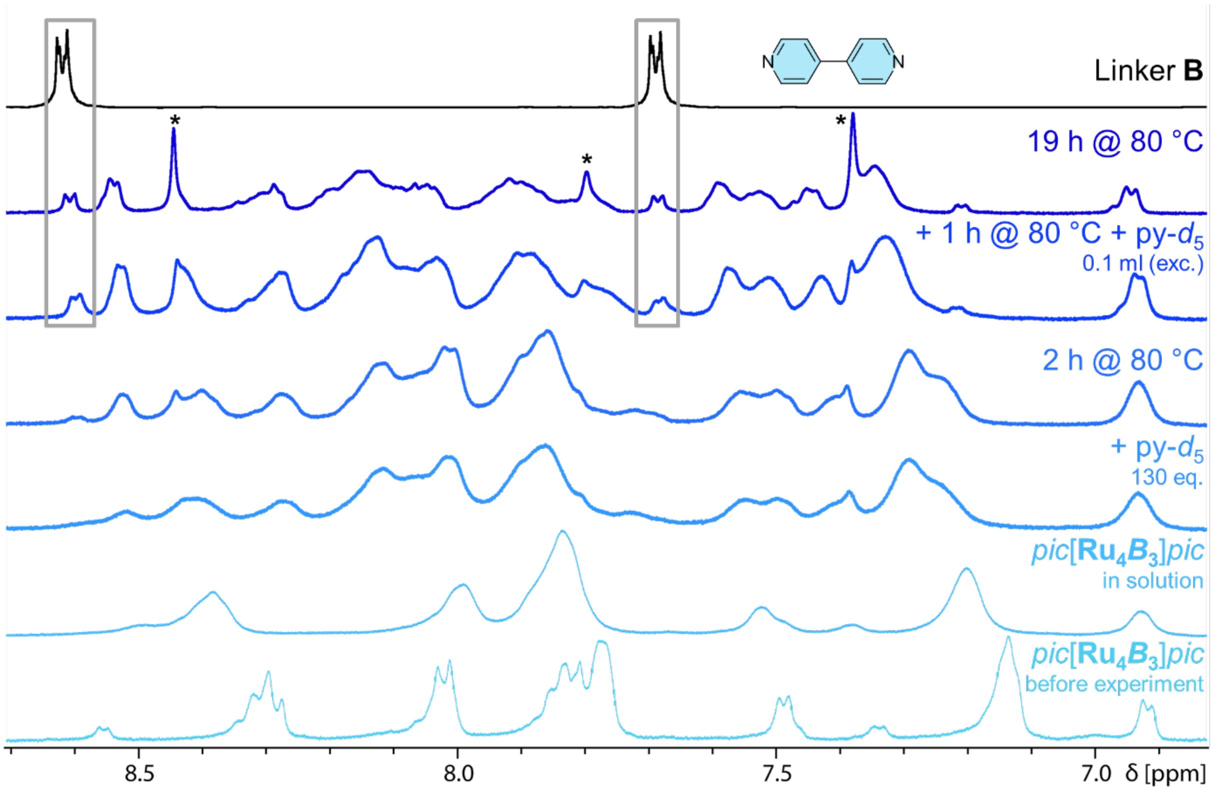

Figure S38. ^{1}H NMR monitoring (400 MHz, TFE-d_3; starting material: CD_2Cl_2/TFE-d$_3$ 9:1, ascorbic acid) of the aromatic region for scrambling experiment of *pic*[**Ru$_4$*B*$_3$**]*pic* with 130 eq. of pyridine-d_5: blue hues from bottom to top: pure *pic*[**Ru$_4$*B*$_3$**]*pic*; dissolved *pic*[**Ru$_4$*B*$_3$**]*pic* at start of experiment (signal broadening is due to the absence of ascorbic acid); directly after addition of pyridine-d_5 (130 eq.); after heating to reflux for 2 h with pyridine-d_5 (130 eq.); heating to reflux for one additional after addition of a high excess (0.1 ml) of pyridine-d_5; after heating to reflux for 19 h with pyridine-d_5 (asterisks mark signals corresponding to residual pyridine signals); black: free linker **B** for comparison. Arising signals that can be attributed to free linkers are highlighted in blue. Due to the changes in the chemical shift caused by the variation of the solvent mixture, the x axis of the spectra was adjusted to create the overlay spectrum shown here.

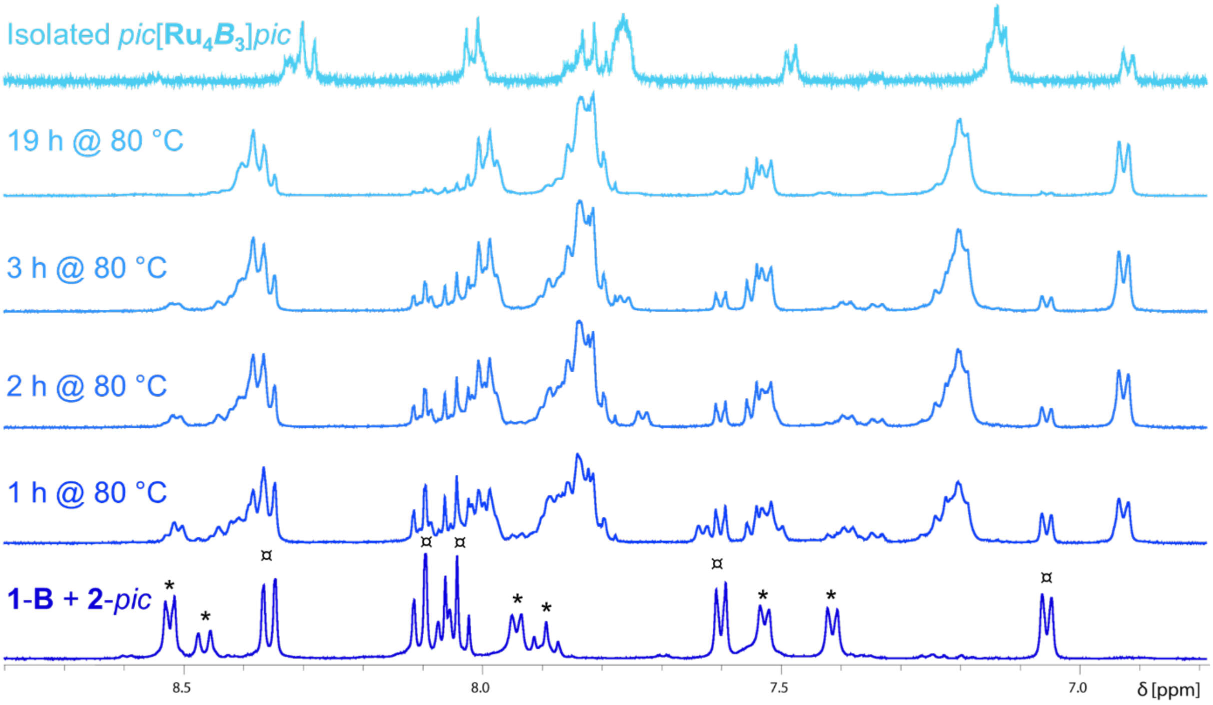

Figure S39. ^{1}H NMR (400 MHz, TFE-d_3, ascorbic acid; isolated product: CD_2Cl_2/TFE-d_3 9:1, ascorbic acid) reaction monitoring of the aromatic region for the reaction of **1-B** and **2**-*pic* at 80 °C towards *pic*[**Ru**$_4$***B***$_3$]*pic*: from bottom to top: mixture of starting materials before reaction (* = **1-B**, ¤ = **2**-*pic*); after 1 h at 80 °C; after 2 h at 80 °C; after 3 h at 80 °C; after 19 h at 80 °C; isolated product. Signal shift from last reaction control to final product is due to NMR solvent change.

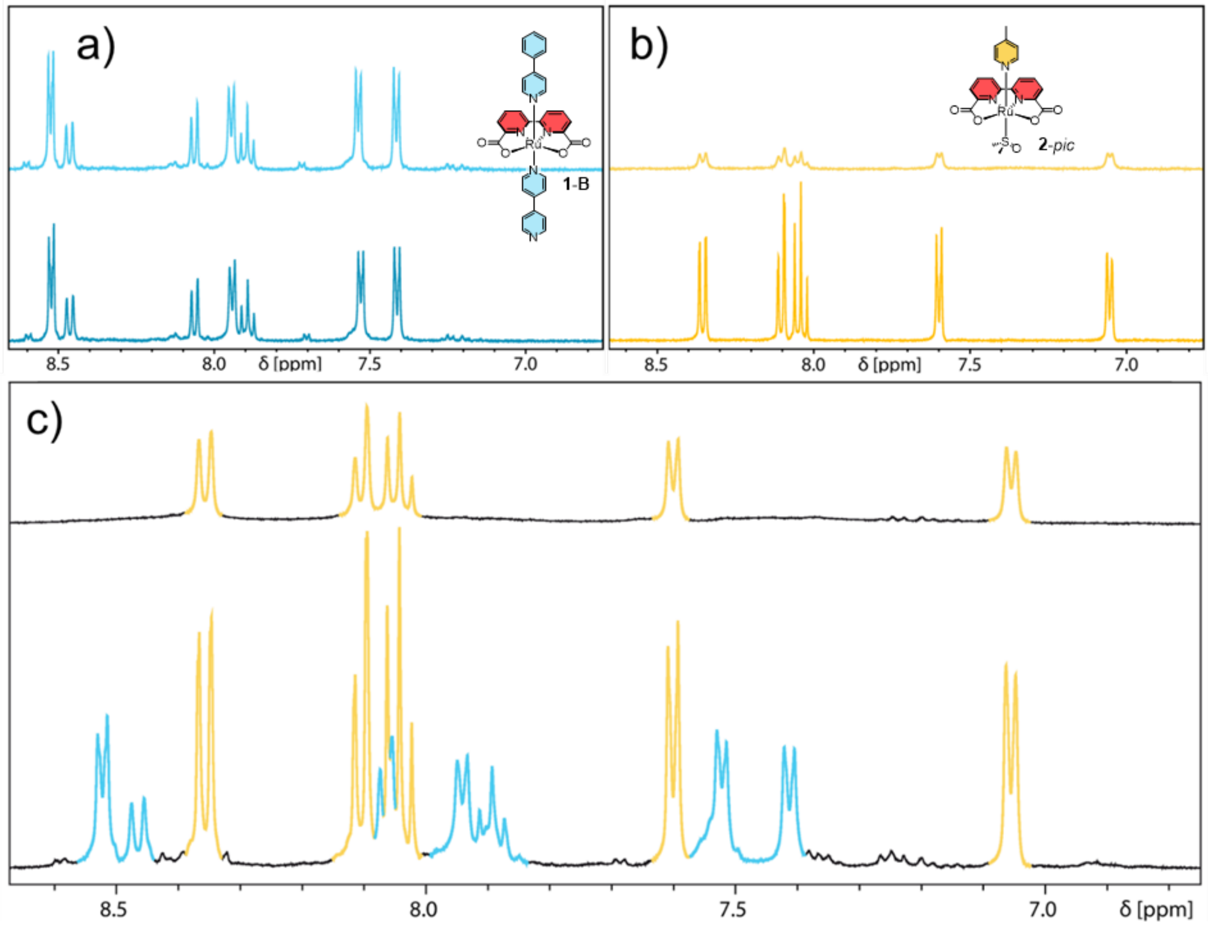

Figure S40. ^{1}H NMR spectra (400 MHz, TFE-d_3, ascorbic acid) of a) **1-B**, b) **2**-*pic*, and c) a mixture of both compounds (2.1 eq. of **2**-*pic*, 1.0 eq. of **1-B**); the bottom spectrum of each box was recorded directly after sample preparation, while the top spectrum was taken after storing the dissolved sample at room temperature for 24 h; spectra of the pure precursors indicate no decomposition, while full conversion of **1-B** and partial conversion of **2**-*pic* was observed in the mixture; for clarity, only the aromatic region of each spectrum is shown.

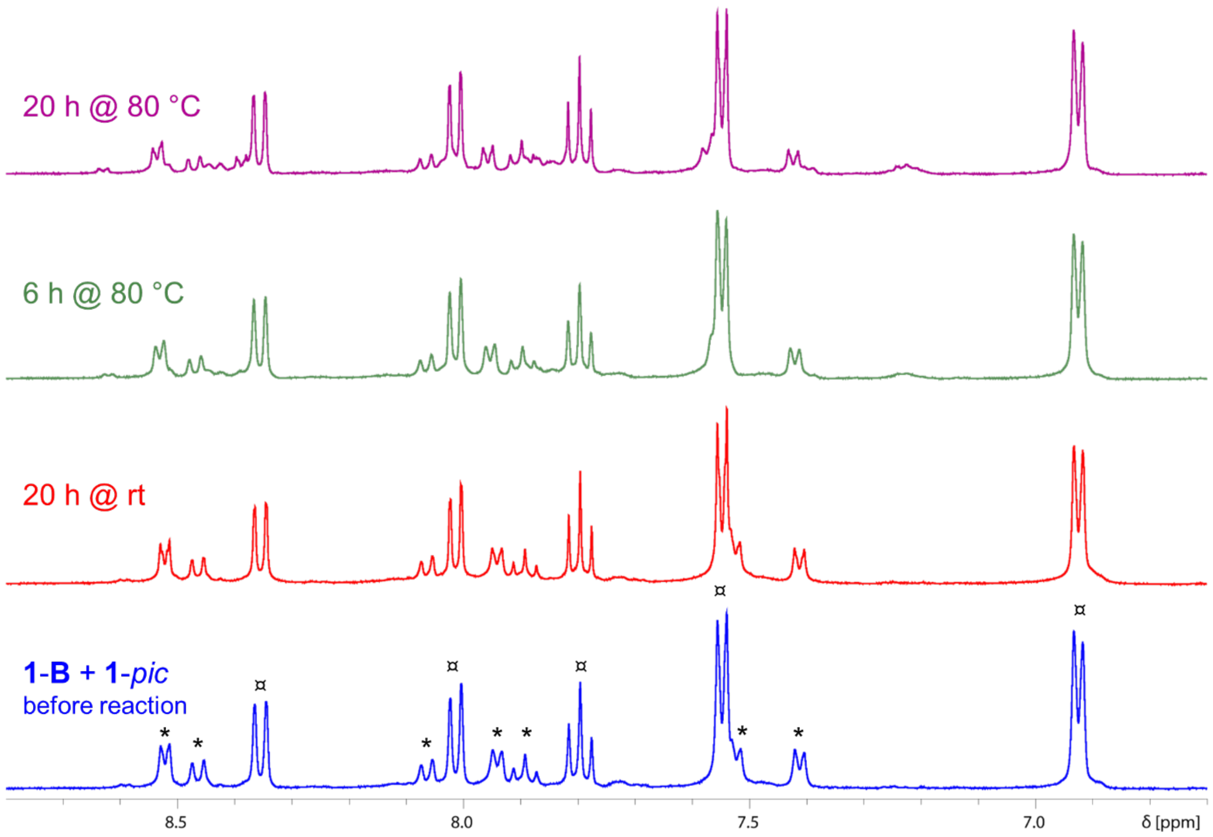

Figure S41. ^{1}H NMR monitoring (400 MHz, TFE-d3, rt, ascorbic acid) for a mixture of **1-B** (*) and **1**-*pic* (¤): spectra were recorded directly after mixing (blue), after 20 h at rt (red), after 6 h at 80 °C (green) and after 20 h at 80 °C (purple); for clarity, only the aromatic region of each spectrum is shown.

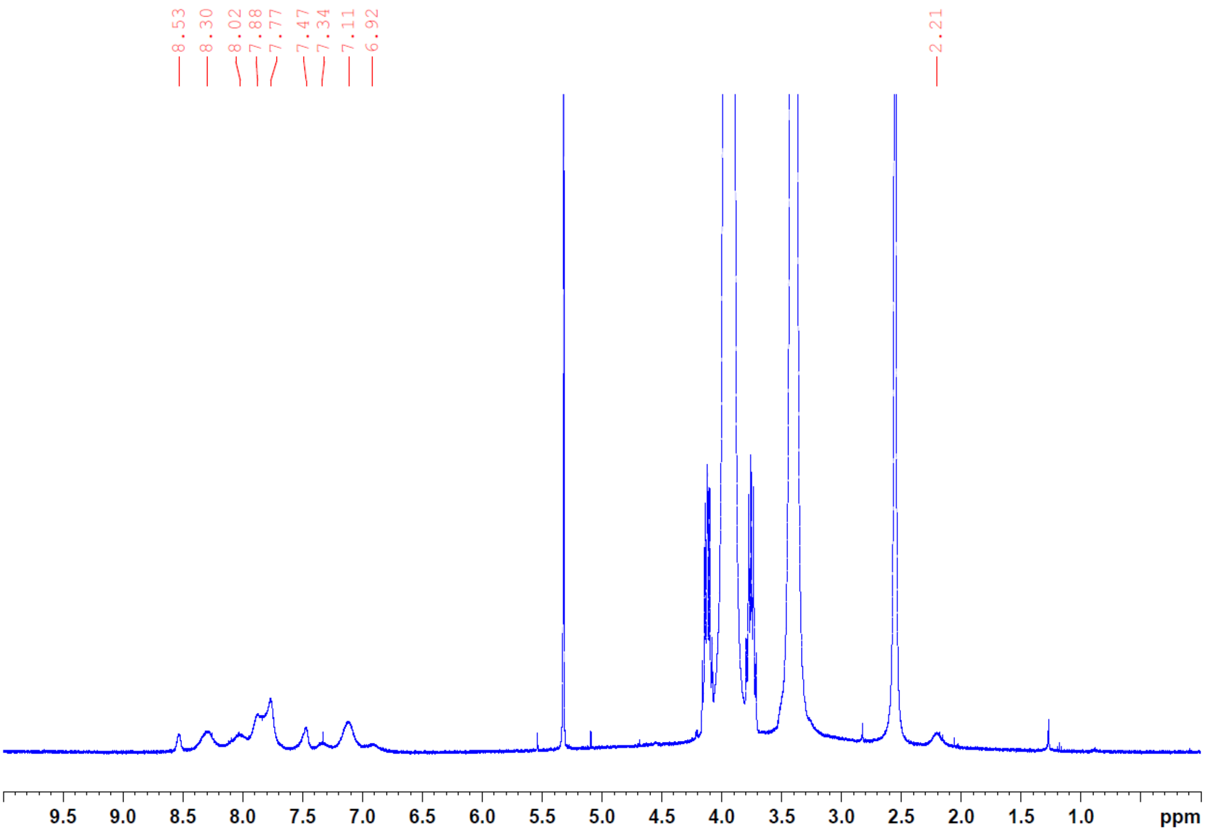

Figure S42. ^{1}H NMR (400 MHz, CD_2Cl_2/TFE-d_3 9:1, ascorbic acid) of an oligomer mixture obtained from the reaction of **1-B** and **2**-*pic* at a total Ru concentration of 5.95 mM.

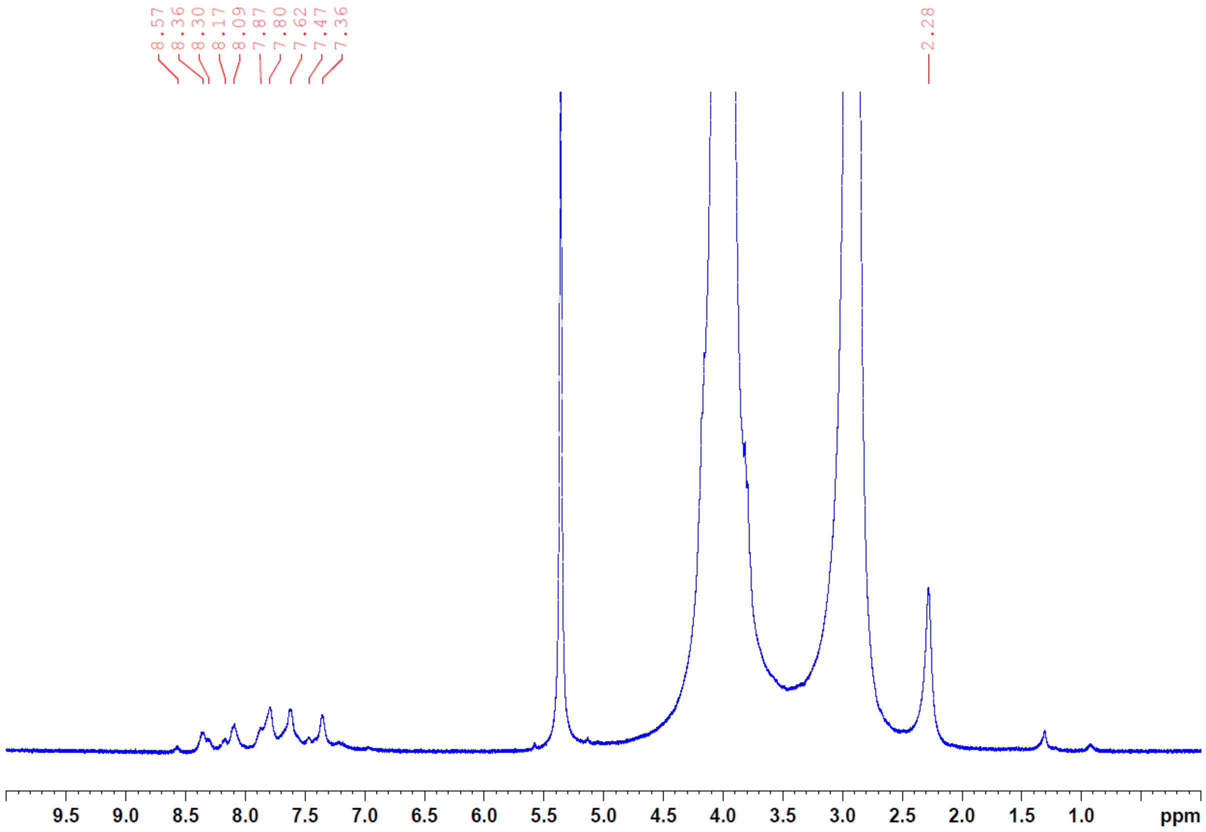

Figure S43. ^{1}H NMR (400 MHz, CD_2Cl_2/TFE-d_3 9:1, ascorbic acid) of an oligomer mixture obtained from the reaction of **1-C** and **2**-*pic* at a total Ru concentration of 7.75 mM.

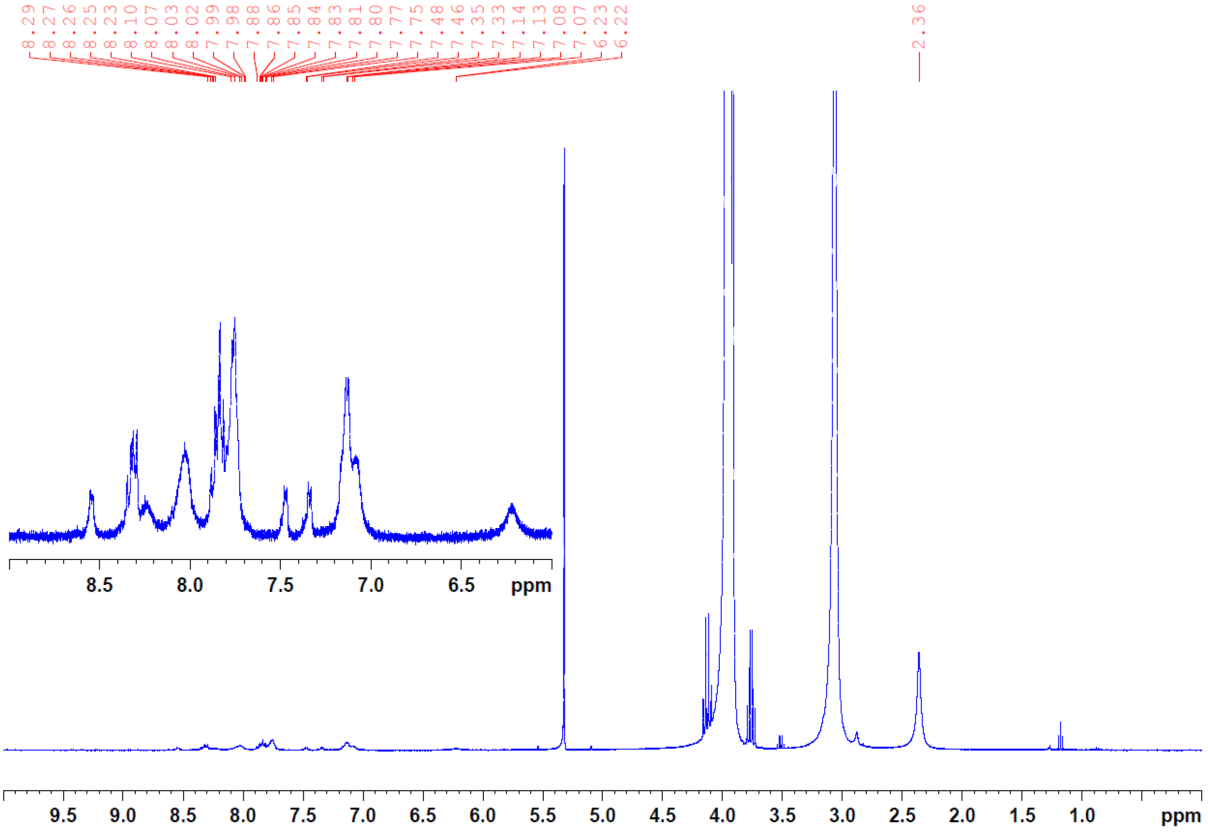

Figure S44. ^{1}H NMR (400 MHz, CD_2Cl_2/TFE-d_3 9:1, ascorbic acid) of an oligomer mixture obtained from the reaction of **1-B** and **2-*dmap*** at a total Ru concentration of 12.4 mM at 78 °C.

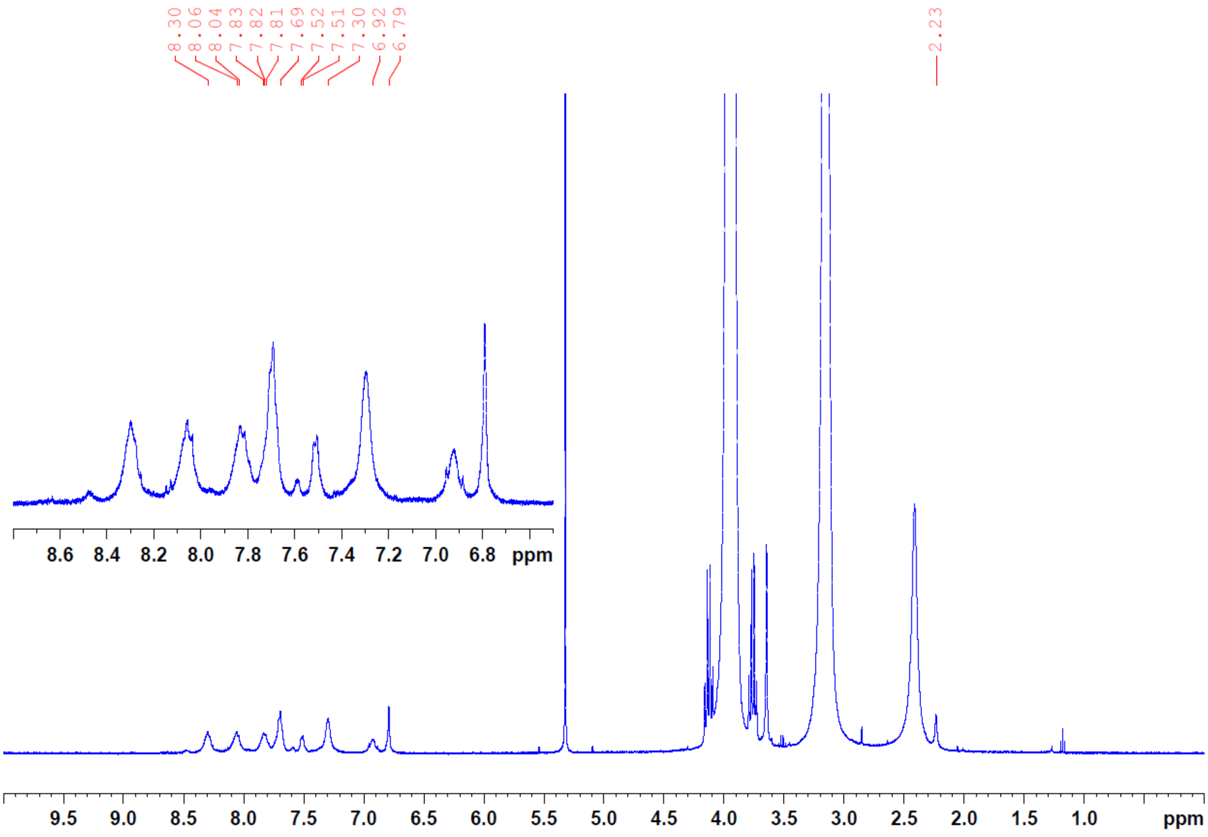

Figure S45. ^{1}H NMR (400 MHz, CD_2Cl_2/TFE-d_3 9:1, ascorbic acid) of an oligomer mixture obtained from the reaction of **1-C^{OMe}** and **2-*pic*** at a total Ru concentration of 7.75 mM.

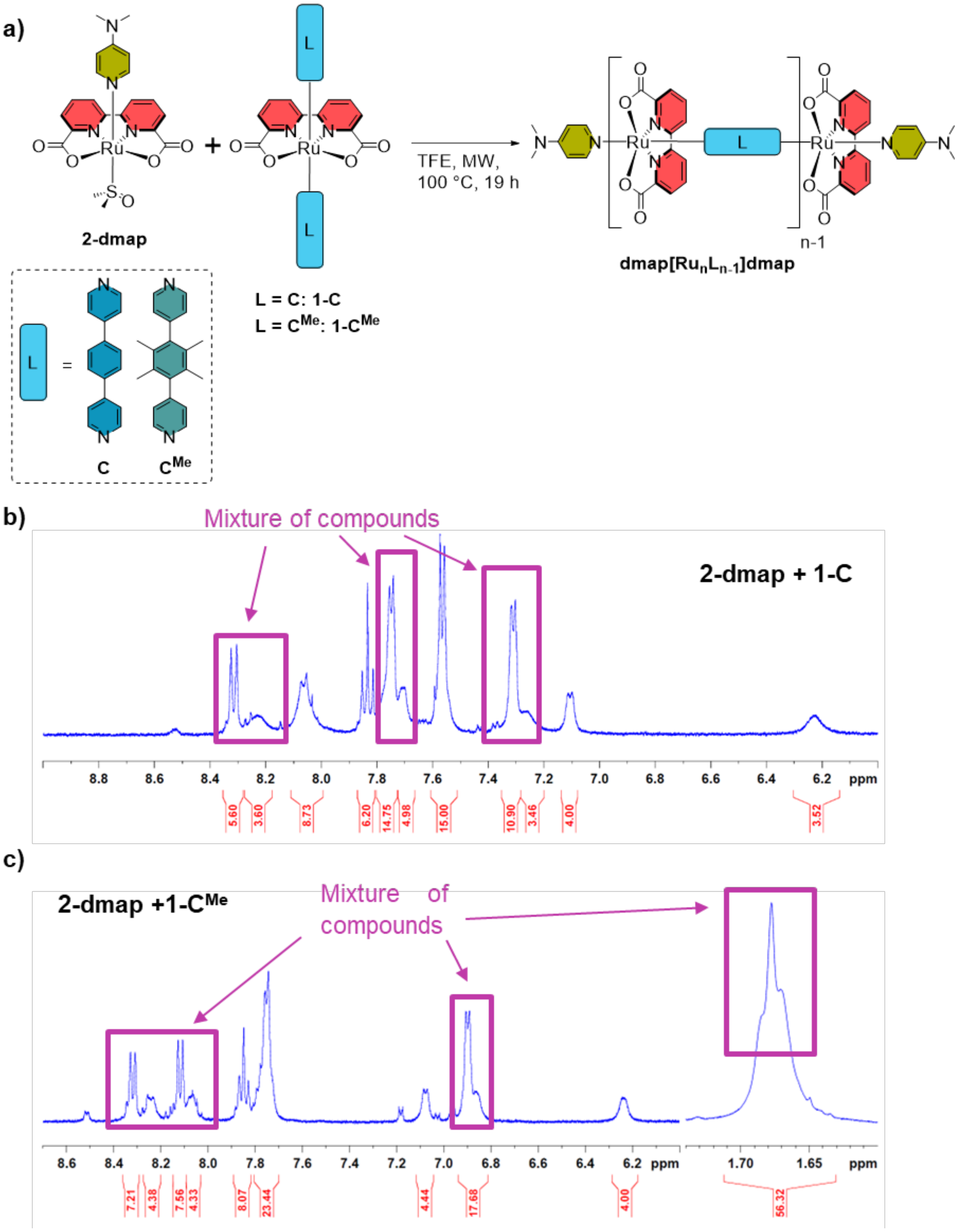

Figure S46. a) Synthesis scheme of the attempts to combine the DMAP end cap with longer linkers. b) ^{1}H NMR spectrum of the experiment with **2-dmap** and **1-C** (conditions: $[Ru_{tot}]$ = 7.75 mM, 19 h, 100 °C, MW). c) ^{1}H NMR spectrum of the experiment with **2-dmap** and **1-C^{Me}** (conditions: $[Ru_{tot}]$ = 3.88 mM, 19 h, 100 °C, MW). In both NMR spectra, unusual signals that indicate a mixture of species are highlighted. Measurement conditions: 400 MHz, dichloromethane-d_2/trifluoroethanol 9:1, ascorbic acid.

Table S3. Screening of reaction conditions for oligomer synthesis with linker **A**.

**entry**	molar ratio **1-A : 2-*pic***	**c(Ru)$_{tot}$** [mM]	**T** [°C]	**duration** [h]	**product**
1	1:2.1	**20.5**	80	19	*undefined oligomer mixture*
2	1:2.1	**11.6**	80	19	*no oligomer, only soluble product*
3	1:2.1	**7.9**	80	19	*oligomer mixture with well-defined signals*
4	1:2.1	20.5	**100**[a]	19	
5	1:2.1	20.5	**100**[b]	19	*oligomer mixture with well-defined signals*
6	1:2.1	20.5	**120**[a]	19	
7	1:2.1	**11.6**	**60**	19	*undefined oligomer mixture*
8	1:2.1	**11.6**	80	**6**	*no oligomer, only soluble product*
9	1:2.1	**41**	**100**[a]	19	
10	1:2.1	**10.3**	**100**[a]	19	*oligomer mixture with well-defined signals*
11	1:2.1	**5.1**	**100**[a]	19	

[a] reaction carried out in microwave reactor; [b] reaction carried out in pressure vessel.

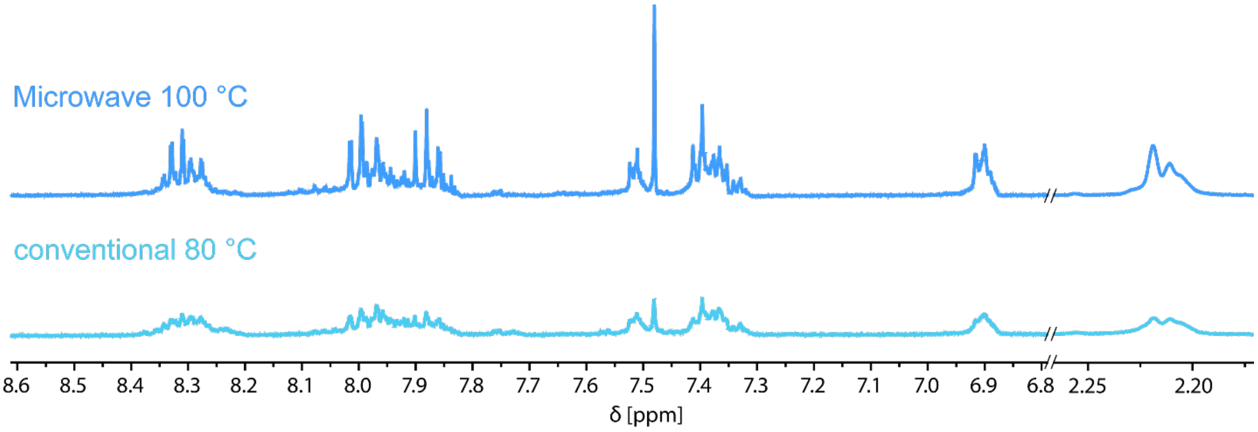

Figure S47. ^{1}H NMR spectra (400 MHz, CH_2Cl_2/TFE-d_3 9:1, ascorbic acid) of reaction mixtures obtained from **1-A** and **2**-*pic* in a microwave reactor at 100 °C (top, entry 4 in Table S3) and in an oil bath at 80 °C (bottom, entry 1 in Table S3).

Table S4. Screening of reaction conditions for oligomer synthesis with linker **B**; deviations from the standard procedure are highlighted in bold.

entry	molar ratio 1-B : 2-*pic*	c(Ru)tot [mM]	T [°C]	duration [h]	product
1	1:2.1	23.8	80	19	*pic*[Ru_4*B*$_3$]*pic*
2	1:2.1	11.9	80	19	*pic*[Ru_5*B*$_4$]*pic*
3	1:2.1	**8.30**	80	19	*oligomer mixture*
4	1:2.1	**5.95**	80	19	
5	1:2.1	23.8	**60**	19	*unsymmetric oligomer fragments*
6	1:2.1	23.8	**100**[a]	19	*pic*[Ru_4*B*$_3$]*pic*
7	1:2.1	11.9	**100**[a]	19	*pic*[Ru_5*B*$_4$]*pic*
8	1:2.1	11.9	80	**6**	*pic*[Ru_4*B*$_3$]*pic*
9	**1:42**	**39.8**	80	19	*no oligomer, only soluble product*
10	**10:1**	**3.37**	80	19	*uncapped oligomer fragments*
11	**1:17**	**27.5**	80	19	*no oligomer, only soluble product*
12	**1:10**	**168**	80	**3**	

[a] reaction was carried out in a microwave reactor.

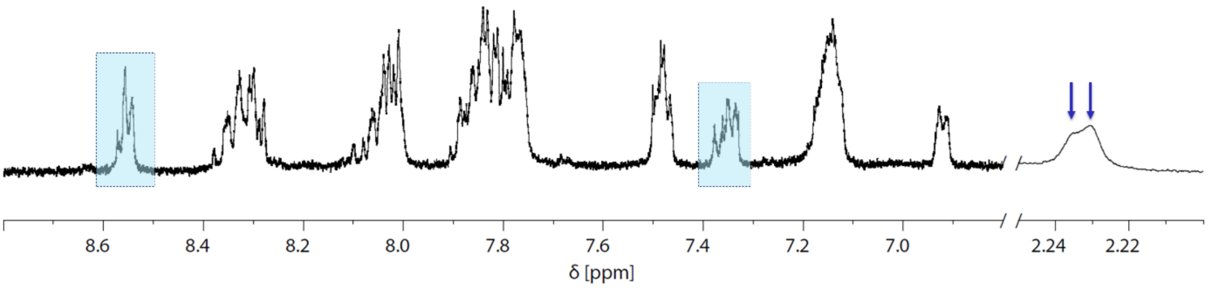

Figure S48. ^{1}H NMR spectrum (400 MHz, CH_2Cl_2/TFE-d_3 9:1, rt, ascorbic acid) of the mixture obtained from the reaction of **1-B** and **2**-*pic* in TFE at 60 °C (entry 5 in Table S4); signals corresponding to terminal linkers **B** are highlighted in light blue, blue arrows indicate the two signals for the *pic* methyl groups, which serve as additional proof for the presence of a mixture of species.

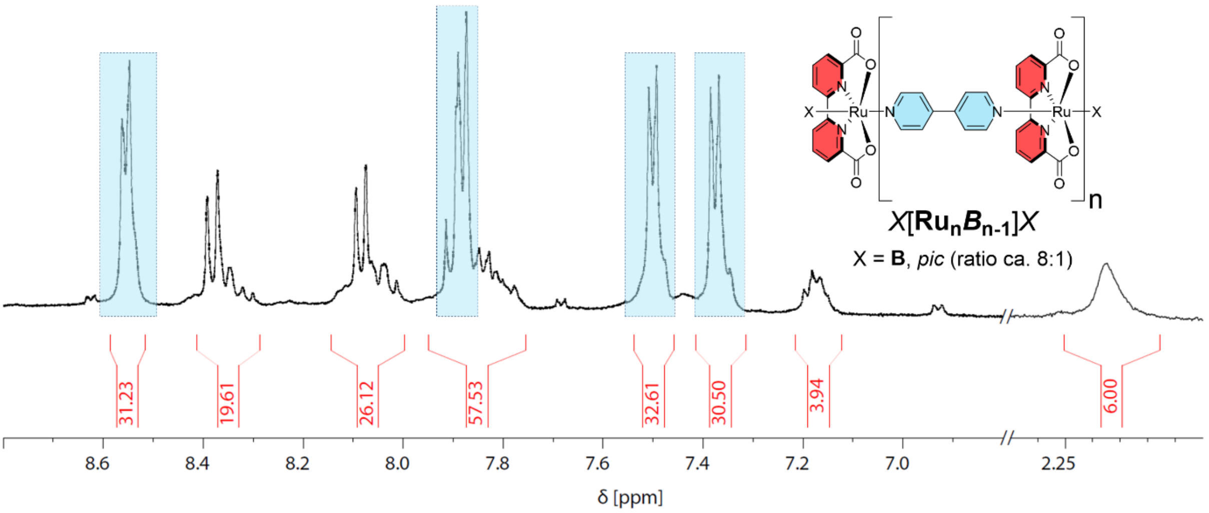

Figure S49. ^{1}H NMR spectrum (400 MHz, CD_2Cl_2/TFE-d_3 9:1, rt, ascorbic acid) of the mixture obtained from the reaction of a 10-fold excess of **1**-**B** and **2**-*pic* in TFE at 80 °C (entry 10 in Table S4); signals corresponding to terminal linkers **B** are highlighted in light blue; integrals were calibrated to the *pic* methyl group at 2.23 ppm and the very high relative integrals of the aromatic signals indicate a minor fraction of *pic* end-caps in the product mixture (end-cap ratio **B**:*pic* ~ 8:1).

Table S5. Screening of reaction conditions for oligomer synthesis with linker **B** and end-cap *dmap*.

**entry**	molar ratio **1-B : 2-***dmap*	**c(Ru)$_{tot}$** [mM]	**T** [°C]	**duration** [h]	**product**
1	1:2.1	12.4	80	19	*incompletely capped oligomer*
2	1:2.1	12.4	80^a	19	
3	1:2.1	12.4	100^a	19	*dmap*[**Ru$_4$*B*$_3$**]*dmap*
4	1:2.1	6.20	100^a	19	*dmap*[**Ru$_4$*B*$_3$**]*dmap*
5	1:2.1	3.88	100^a	19	*dmap*[**Ru$_4$*B*$_3$**]*dmap*

^{a} reaction was carried out in a microwave reactor.

Table S6. Screening of reaction conditions for oligomer synthesis with linker **C**.

**entry**	molar ratio **1**-**C** : **2**-*pic*	**c(Ru)$_{tot}$** [mM]	**T** [°C]	**duration** [h]	**product**
1	1:2.1	7.75	80	19	*pic*[**Ru$_4$*C*$_3$**]*pic*
2	1:2.1	3.88	80	19	*pic*[**Ru$_5$*C*$_4$**]*pic*
3	1:2.1	7.75	**100**[a]	19	*pic*[**Ru$_4$*C*$_3$**]*pic*
4	1:2.1	3.88	**100**[a]	19	*pic*[**Ru$_5$*C*$_4$**]*pic*

[a] reaction was carried out in a microwave reactor.

Table S7. Screening of reaction conditions for oligomer synthesis with linker **C^{OMe}**.

**entry**	molar ratio **1**-**C^{OMe}** : **2**-*pic*	**c(Ru)$_{tot}$** [mM]	**T** [°C]	**duration** [h]	**product**
1	1:2.1	7.75	80	19	*pic*[**Ru$_4$*C*^{OMe}$_3$**]*pic*
2	1:2.1	3.88	80	19	*pic*[**Ru$_4$*C*^{OMe}$_3$**]*pic*
3	1:2.1	3.88	100[a]	19	*pic*[**Ru$_4$*C*^{OMe}$_3$**]*pic*

[a] reaction was carried out in a microwave reactor.

Table S8. Screening of reaction conditions for oligomer synthesis with linker **C^{Me}**.

**entry**	molar ratio **1**-**C^{Me}** : **2**-*pic*	**c(Ru)$_{tot}$** [mM]	**T** [°C]	**duration** [h]	**product**
1	1:2.1	7.75	100[a]	19	*pic*[**Ru$_4$*C*^{Me}$_3$**]*pic*
2	1:2.1	7.75	80	19	*pic*[**Ru$_5$*C*^{Me}$_4$**]*pic*
3	1:2.1	3.88	80	19	*pic*[**Ru$_5$*C*^{Me}$_4$**]*pic*
4	1:2.1	3.88	100[a]	19	*pic*[**Ru$_4$*C*^{Me}$_3$**]*pic*

[a] reaction was carried out in a microwave reactor.

5 Diffusion-Ordered NMR Spectroscopy (DOSY)

Processing of DOSY data: First, the reference frequency of the DOSY spectrum was adjusted to that of the ^{1}H spectrum that was recorded before. After manual phase correction, all relevant signals were integrated, and the resulting decay curves were fitted by intensity to obtain diffusion coefficients for each signal. From this list, data points with an ill fit or clear overlap with other species were removed. A median value was calculated from the remaining entries to obtain the diffusion coefficient before calibration.

Tetrakis(trimethylsilyl)silane (TMSS) was added as a calibration standard. The calibrated diffusion coefficient was calculated using equation S1:

$$D_{cal} = \frac{D_{measured} \cdot D_{stan,lit}}{D_{stan,meas}} \quad \text{(S1)}$$

with $D_{measured}$ being the uncalibrated diffusion coefficient of the sample, $D_{stan,lit}$ a literature-known reference value for the diffusion coefficient of the standard and $D_{stan,meas}$ the diffusion coefficient of the standard observed experimentally.

$D_{stan,meas}$ was calculated similar to $D_{measured}$. $D_{stan,lit}$ was set to 1.42×10^{-9} m^2 s^{-1}. [S13] Due to limited literature data, the reference value used was recorded in pure CD_2Cl_2 at 298.15 K instead of a 9:1 CD_2Cl_2-TFE-*d*$_3$ mixture at 295.6 K.

Calculation of oligomer length: The oligomer length L was calculated from the diffusion coefficient using a formula for cylindrical objects (equation S2): [S14]

$$D_t = \frac{k_B T \cdot [3\, ln(L/w) + 2\gamma_{\parallel} + \gamma_{\perp}]}{8\pi\eta L} \quad \text{(S2)}$$

with D_t being the translational diffusion coefficient of the sample, k_B the Boltzmann constant, T the absolute temperature for the measurement (295.6 K), L and w the oligomer length and width (w and initial values for L were obtained by molecular modelling), η the viscosity of the solvent mixture, and $\gamma_{\parallel}$ and $\gamma_{\perp}$ end-correction coefficients.

The correction coefficients were calculated using the equations given in **Table S9**.

Table S9. Formulae for calculating the correction coefficients and the validity range of the method, with p = L/w. [S15]

$\gamma_{\parallel}$	$\gamma_{\perp}$	**Validity**
$-0.207 + \frac{0.980}{p} - \frac{0.133}{p^2}$	$0.839 + \frac{0.185}{p} + \frac{0.233}{p^2}$	$2 < p < 30$

The viscosity η was calculated using the Arrhenius equation (equation S3) for ideal solvent mixtures: [S16]

$$ln\,\eta = X_{DCM}\,ln\,\eta_{DCM} + X_{TFE}\,ln\,\eta_{TFE} \quad (S3)$$

with X_{DCM} being the mole fraction of dichloromethane-d_2, X_{TFE} the mole fraction of triflouroethanol-d_3, η_{TFE} the viscosity of triflouroethanol-d_3 and η_{DCM} the viscosity of CD_2Cl_2.

Values for the mole fractions were derived from the mixture ratio of the solvents (CD_2Cl_2/TFE 9:1 v/v). η_{TFE} and η_{DCM} were calculated using equation S4: [S17]

$$\frac{\eta_2}{\eta_1} = \sqrt{\frac{M_2 \cdot M_2}{M_2 + M_2} / \frac{M_1 \cdot M_2}{M_1 + M_2}} \qquad (S4)$$

with η_1 being the viscosity of the non-deuterated solvent, η_2 the viscosity of the deuterated solvent, M_1 the molecular mass of the non-deuterated solvent and M_2 the molecular mass of the deuterated solvent.

η_1 at 295.6 °C was extrapolated from literature values for CD_2Cl_2 and TFE. [S18]

Table S10. Comparison of molecular lengths of Ru(bda) coordination oligomers obtained from ^{1}H DOSY NMR (600 MHz, CH_2Cl_2/TFE-d_3 9:1, 295.6 K, ascorbic acid, TMSS) and molecular modelling (UFF force field, *Materials Studio*).

**entry**	**oligomer**	***D*** [10^{-10} m^2 s^{-1}]	**molecular length** [nm]		
			**force field**	**DOSY**	**deviation**
1	*pic*[**Ru$_4$*B*$_3$**]*pic*	3.84	4.75	**5.60**	0.85
2	*pic*[**Ru$_5$*B*$_4$**]*pic*	3.69	5.82	**6.00**	0.18
3	*pic*[**Ru$_4$*C*$_3$**]*pic*	3.66	6.03	**6.08**[a]	0.05
4	*pic*[**Ru$_5$*C*$_4$**]*pic*	3.24	7.55	**7.46**[a]	0.09
5	*dmap*[**Ru$_4$*B*$_3$**]*dmap*	4.08	4.93	**5.03**	0.10
6	*pic*[**Ru$_4$*C*^{Me}$_3$**]*pic*	3.83	6.02	**5.63**	0.39
7	*pic*[**Ru$_5$*C*^{Me}$_4$**]*pic*	3.30	7.58	**7.24**	0.34
8	*pic*[**Ru$_4$*C*^{OMe}$_3$**]*pic*	3.70	6.01	**5.98**	0.03
9	*pic*[**Ru$_n$*A*$_{n-1}$**]*pic*	5.18	2.79-3.47	**3.21**	–

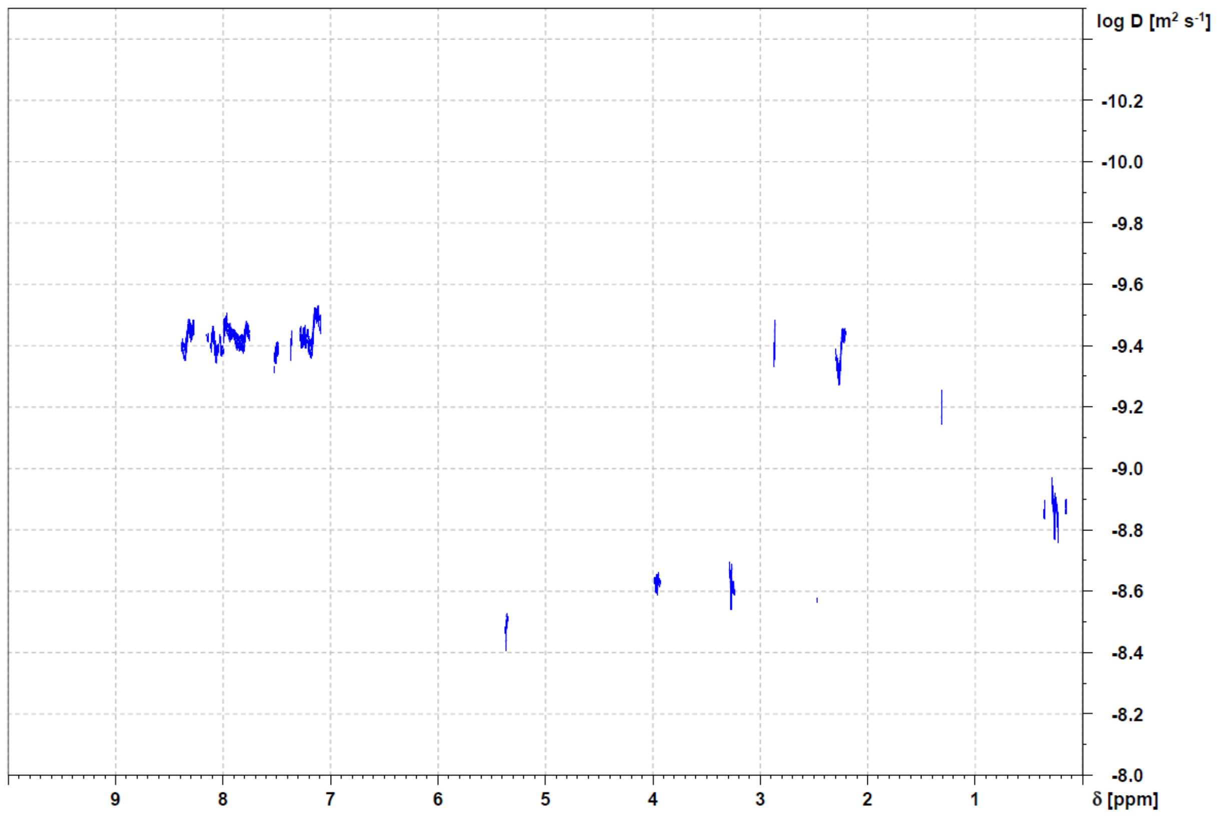

Figure S50. ^{1}H DOSY NMR (led, 600 MHz, CH_2Cl_2/TFE-d_3 9:1, 295.6 K, ascorbic acid, TMSS) of *pic*[**Ru$_4$*B*$_3$**]*pic*.

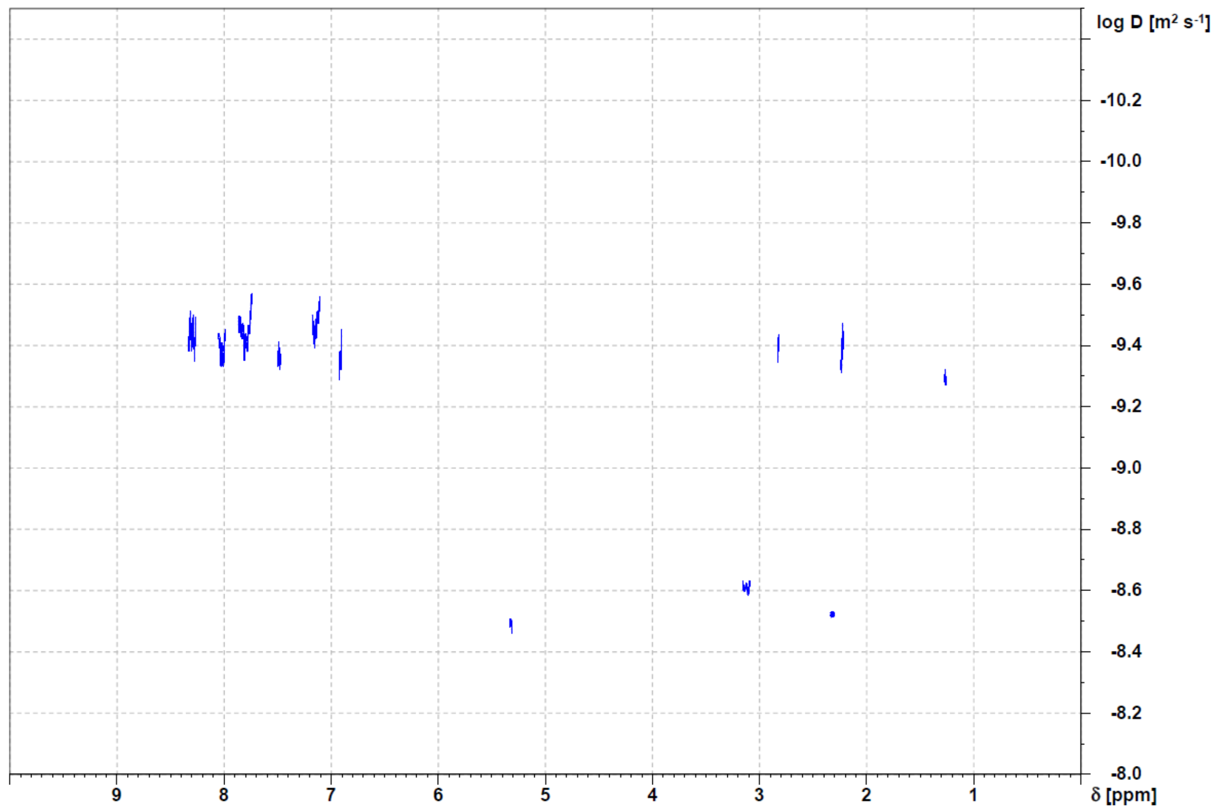

Figure S51. ^{1}H DOSY NMR (dste, 400 MHz, CH_2Cl_2/TFE-d_3 9:1, 295.6 K, ascorbic acid, TMSS) of *pic*[**Ru$_5$*B*$_4$**]*pic*.

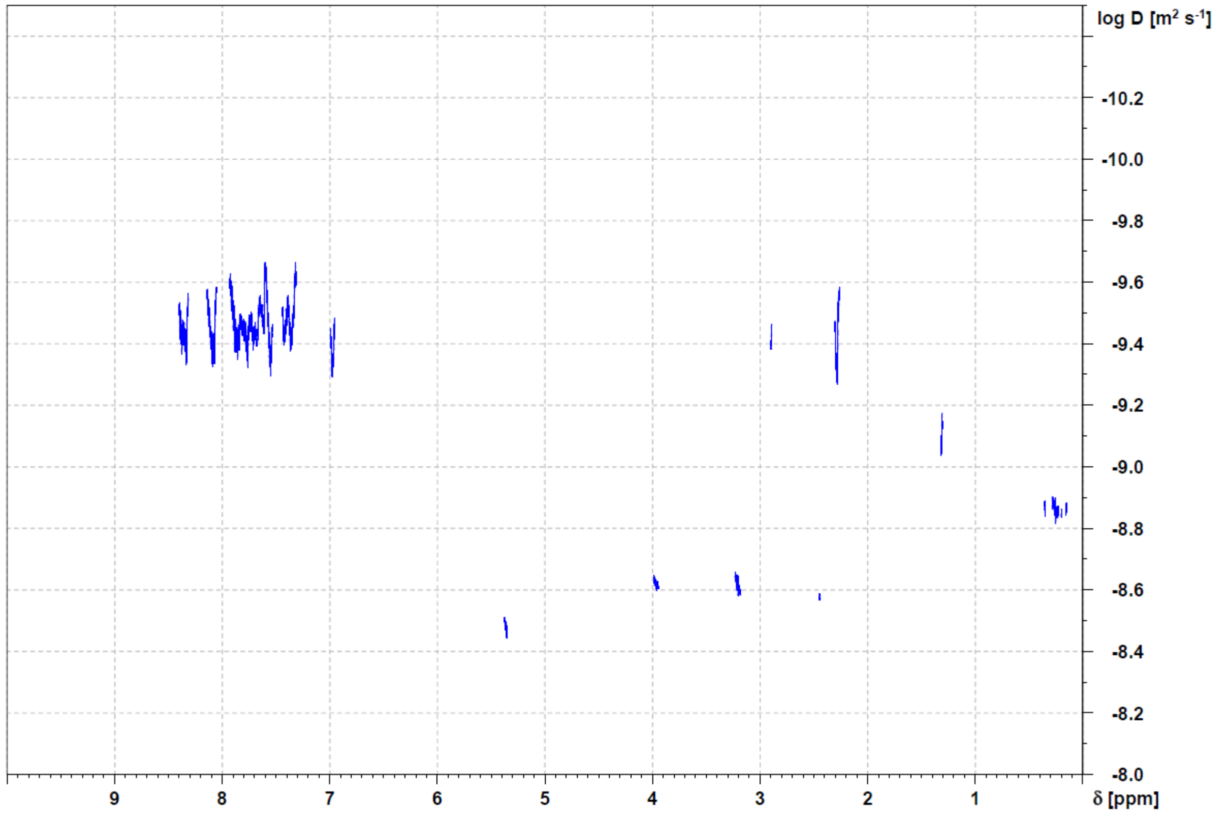

Figure S52. ^{1}H DOSY NMR (led, 600 MHz, CH_2Cl_2/TFE-d_3 9:1, 295.6 K, ascorbic acid, TMSS) of *pic*[**Ru$_4$*C*$_3$**]*pic*.

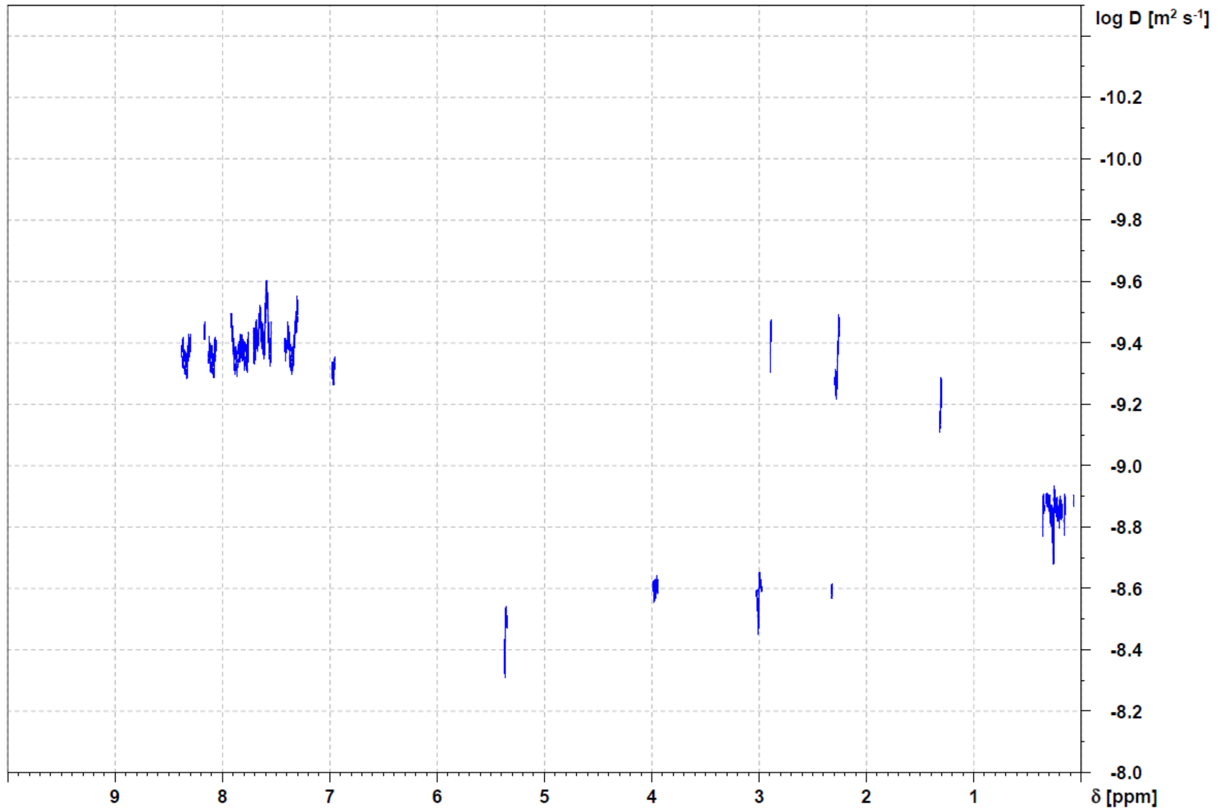

Figure S53. ^{1}H DOSY NMR (led, 600 MHz, CH_2Cl_2/TFE-d_3 9:1, 295.6 K, ascorbic acid, TMSS) of *pic*[**Ru$_5$*C*$_4$**]*pic*.

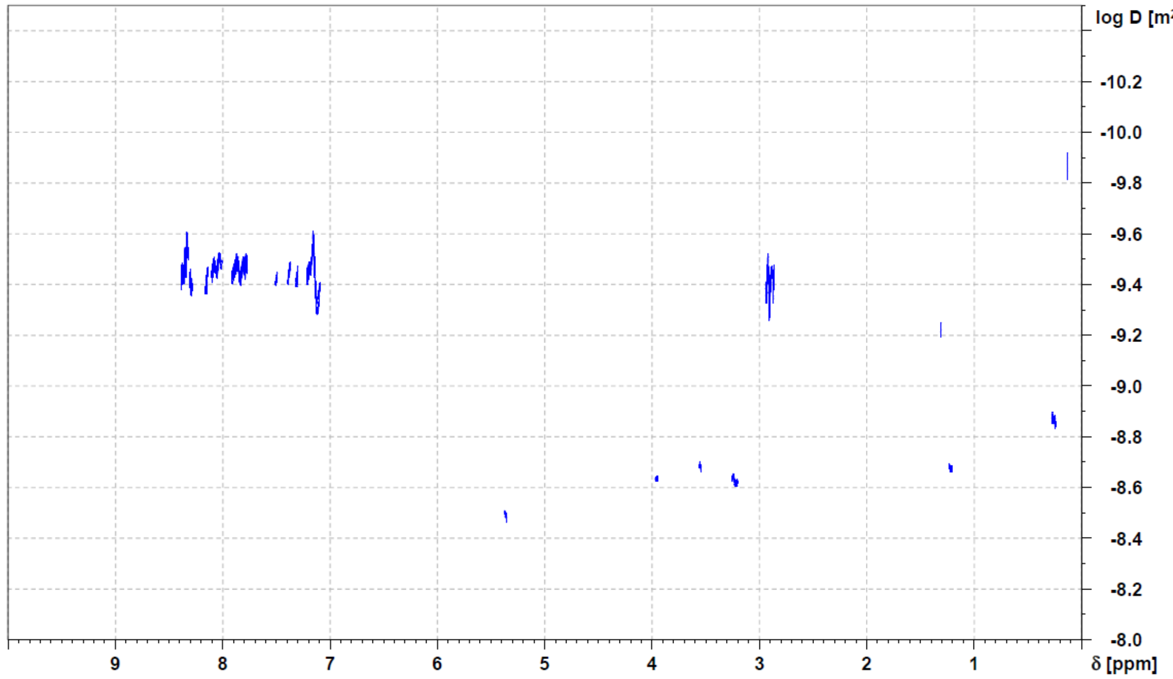

Figure S54. ^{1}H DOSY NMR (led, 600 MHz, CH_2Cl_2/TFE-d_3 9:1, 295.6 K, ascorbic acid, TMSS) of *dmap*[**Ru$_4$*B*$_3$**]*dmap*.

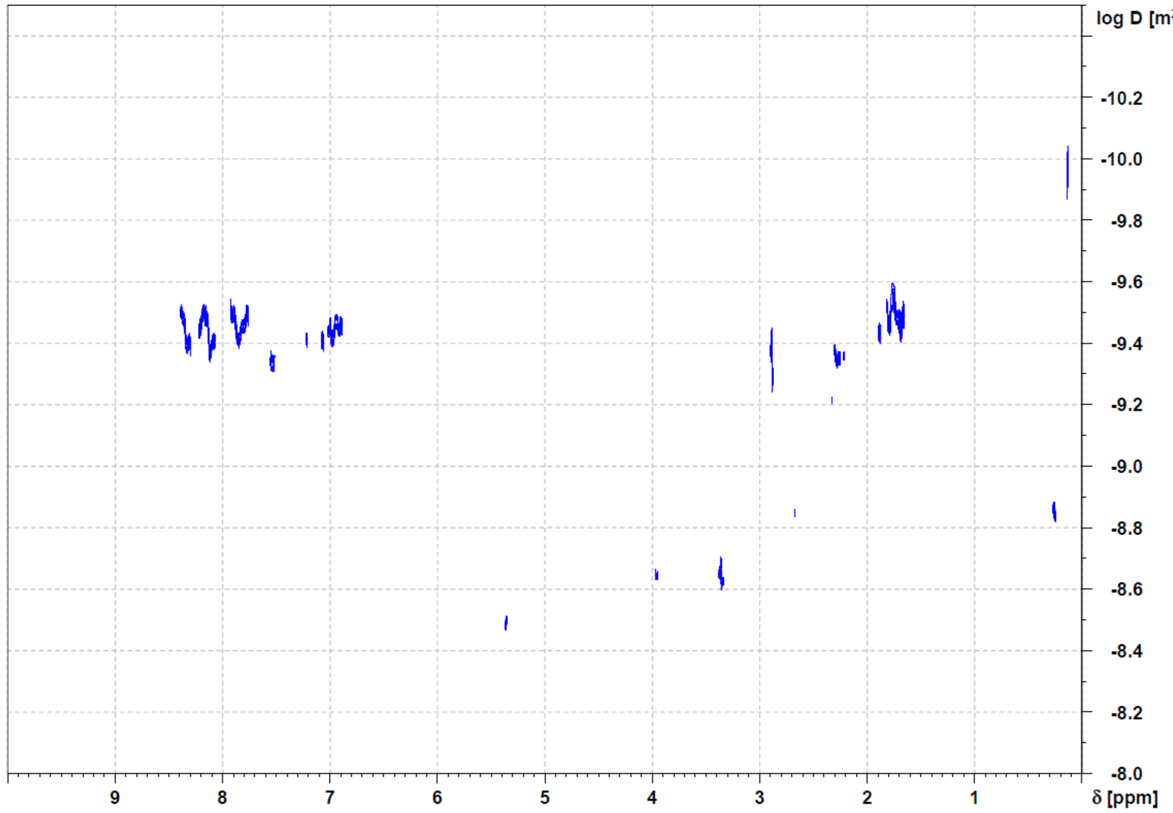

Figure S55. ^{1}H DOSY NMR (dste, 600 MHz, CH_2Cl_2/TFE-d_3 9:1, 295.6 K, ascorbic acid, TMSS) of *pic*[**Ru$_4$*C*^{Me}$_3$**]*pic*.

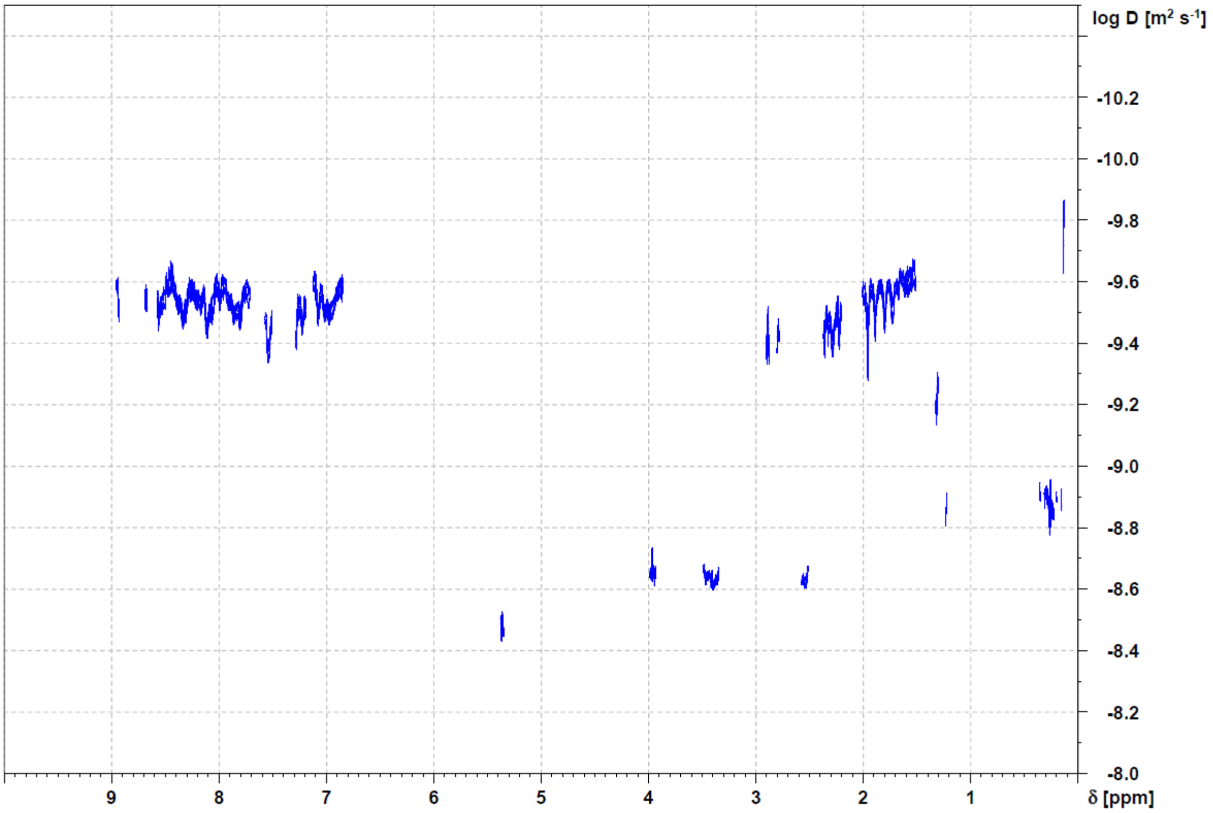

Figure S56. ^{1}H DOSY NMR (led, 600 MHz, CH_2Cl_2/TFE-d_3 9:1, 295.6 K, ascorbic acid, TMSS) of *pic*[$\mathbf{Ru_5}\boldsymbol{C^{Me}}_4$]*pic*.

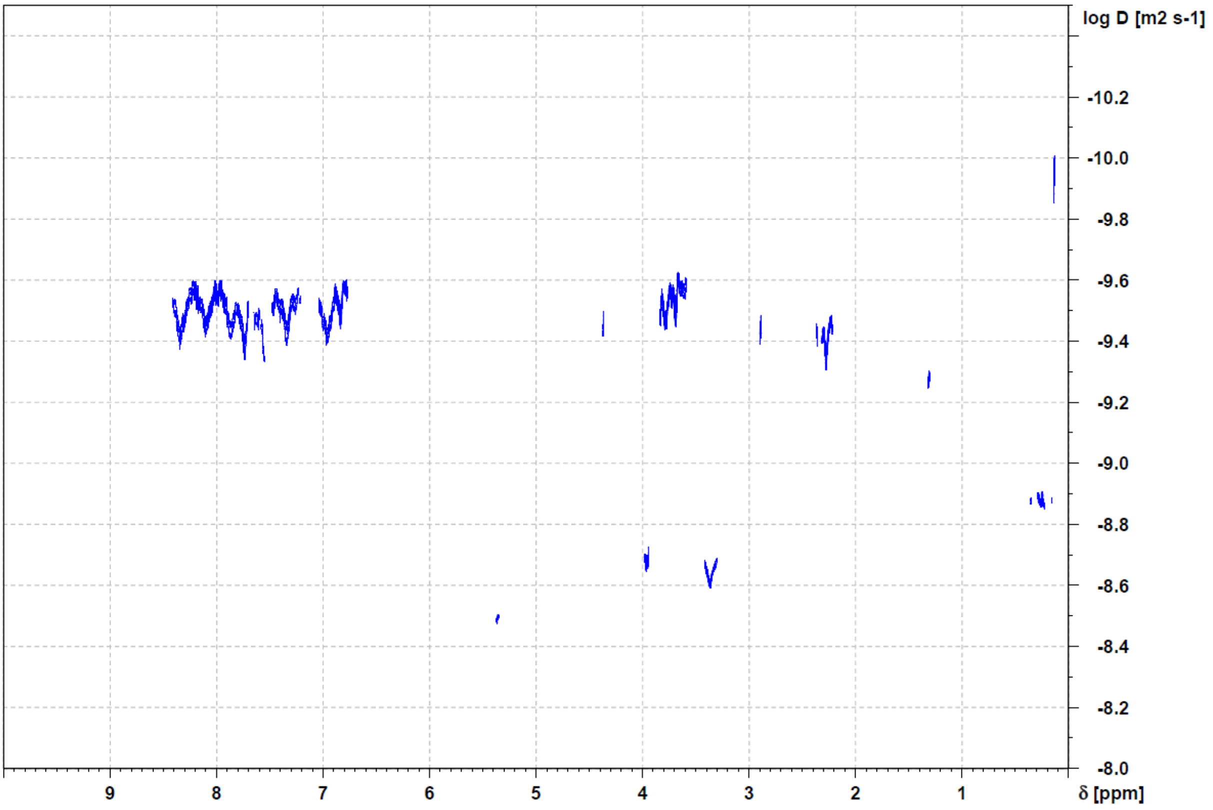

Figure S57. ^{1}H DOSY NMR (led, 600 MHz, CH_2Cl_2/TFE-d_3 9:1, 295.6 K, ascorbic acid, TMSS) of *pic*[$\mathbf{Ru_4}\boldsymbol{C^{OMe}}_3$]*pic*.

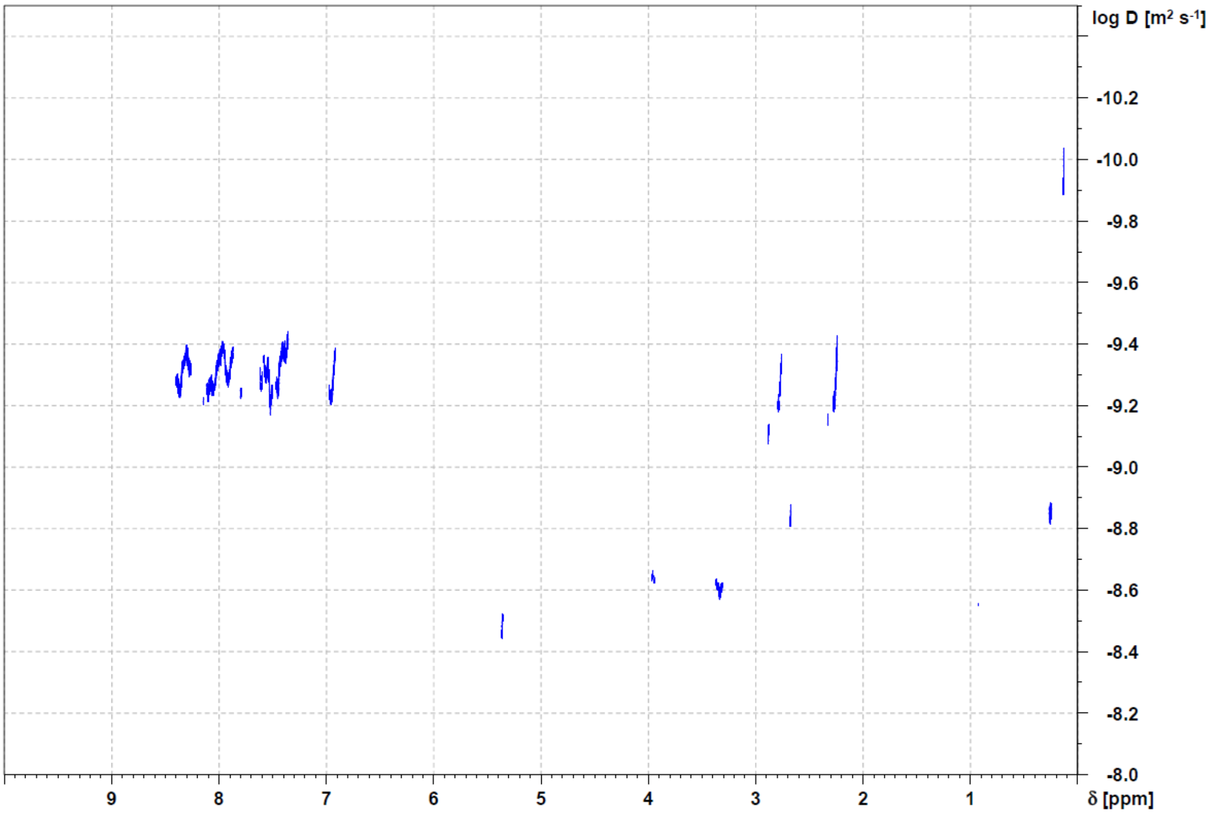

Figure S58. ^{1}H DOSY NMR (dste, 600 MHz, CH_2Cl_2/TFE-d_3 9:1, 295.6 K, ascorbic acid, TMSS) of *pic*[$\mathbf{Ru_n}\boldsymbol{A}_{\mathbf{n-1}}$]*pic*.

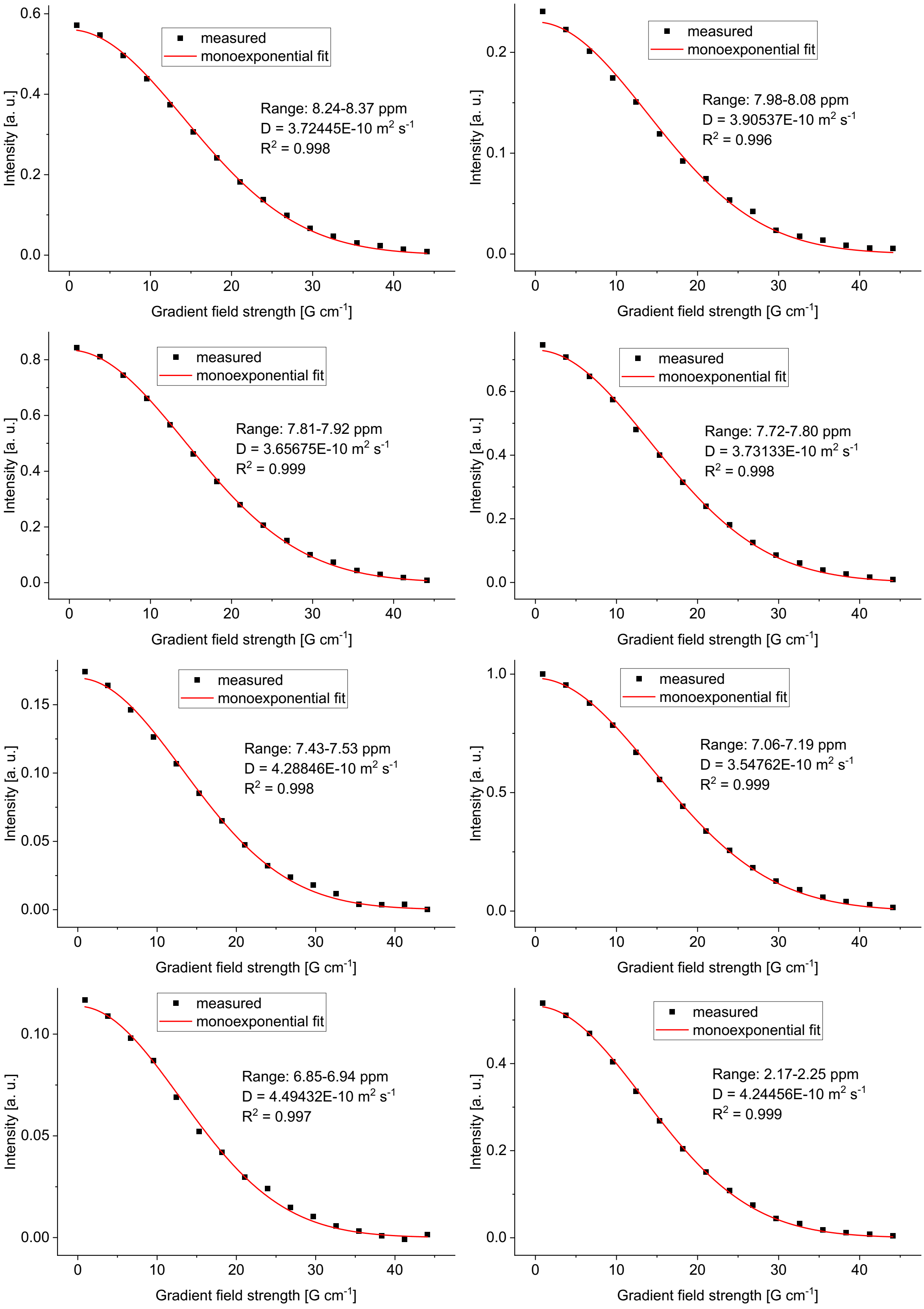

Figure S59. Monoexponential fit curves for the amplitude decay of the proton signals obtained during ^{1}H DOSY NMR measurements of *pic*[**Ru$_4$*B*$_3$**]*pic* (led, 600 MHz, CD_2Cl_2/TFE-*d*$_3$ 9:1, 295.6 K, ascorbic acid, TMSS).

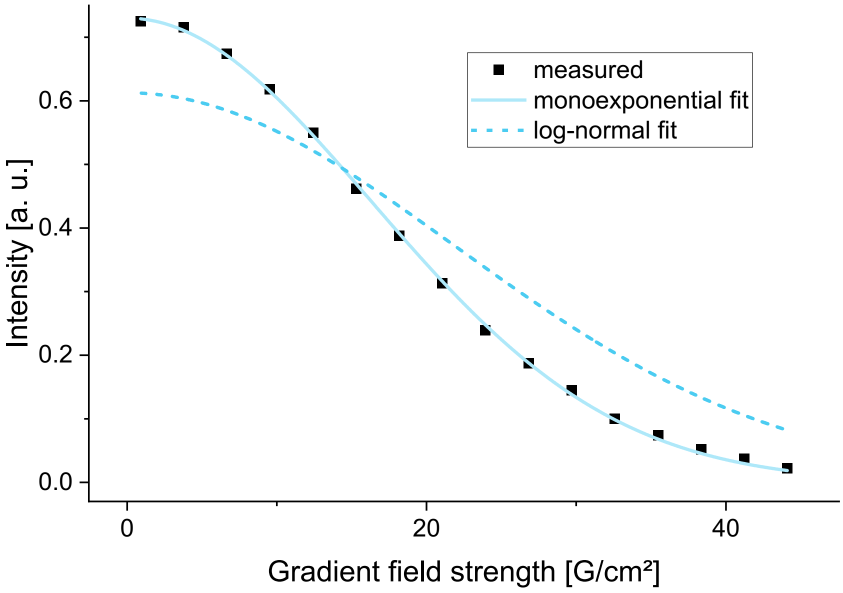

Figure S60. Exemplary amplitude decay curve obtained during ^{1}H DOSY NMR measurements (led, 600 MHz, CD_2Cl_2/TFE-d_3 9:1, 295.6 K, ascorbic acid, TMSS) of *pic*[**Ru$_4$*B*$_3$**]*pic* (measured data points in black, monoexponential fit in solid blue and log-normal fit in dashed blue).

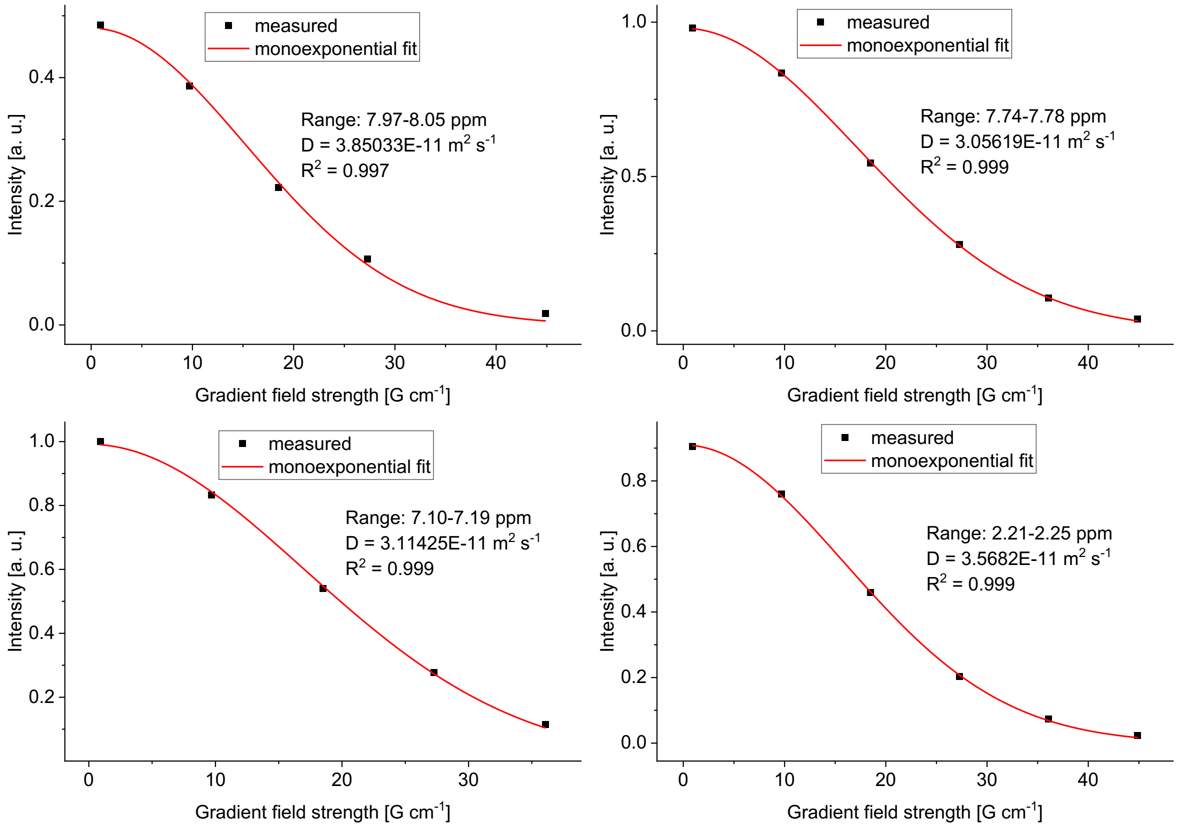

Figure S61. Monoexponential fit curves for the amplitude decay for the proton signals obtained during ^{1}H DOSY NMR measurements of *pic*[**Ru$_5$*B*$_4$**]*pic* (dste, 600 MHz, CD_2Cl_2/TFE-d_3 9:1, 295.6 K, ascorbic acid, TMSS).

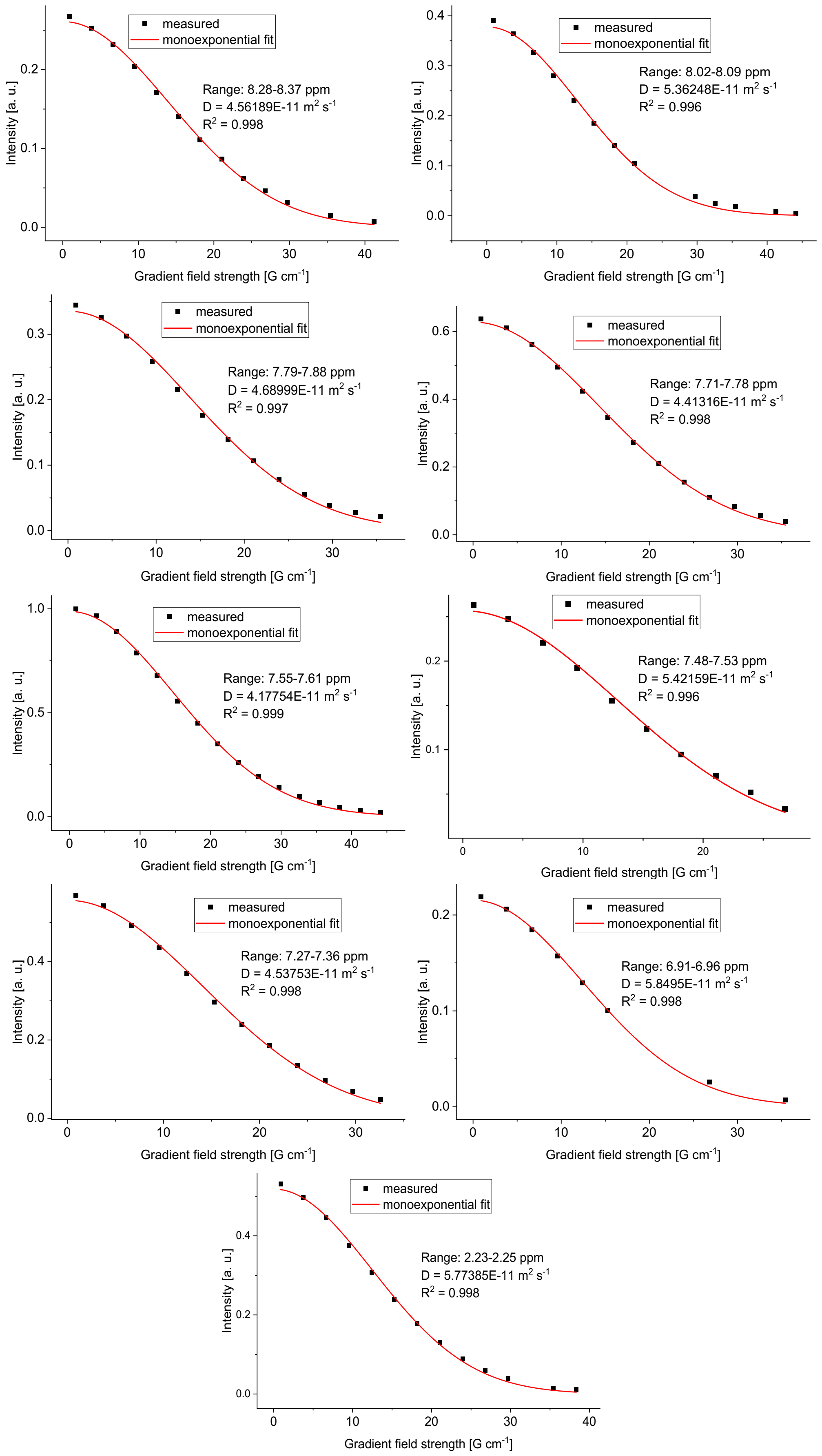

Figure S62. Monoexponential fit curves for the amplitude decay for the proton signals obtained during ^{1}H DOSY NMR measurements of *pic*[**Ru$_4$*C*$_3$**]*pic* (led, 600 MHz, CD_2Cl_2/TFE-d_3 9:1, 295.6 K, ascorbic acid, TMSS).

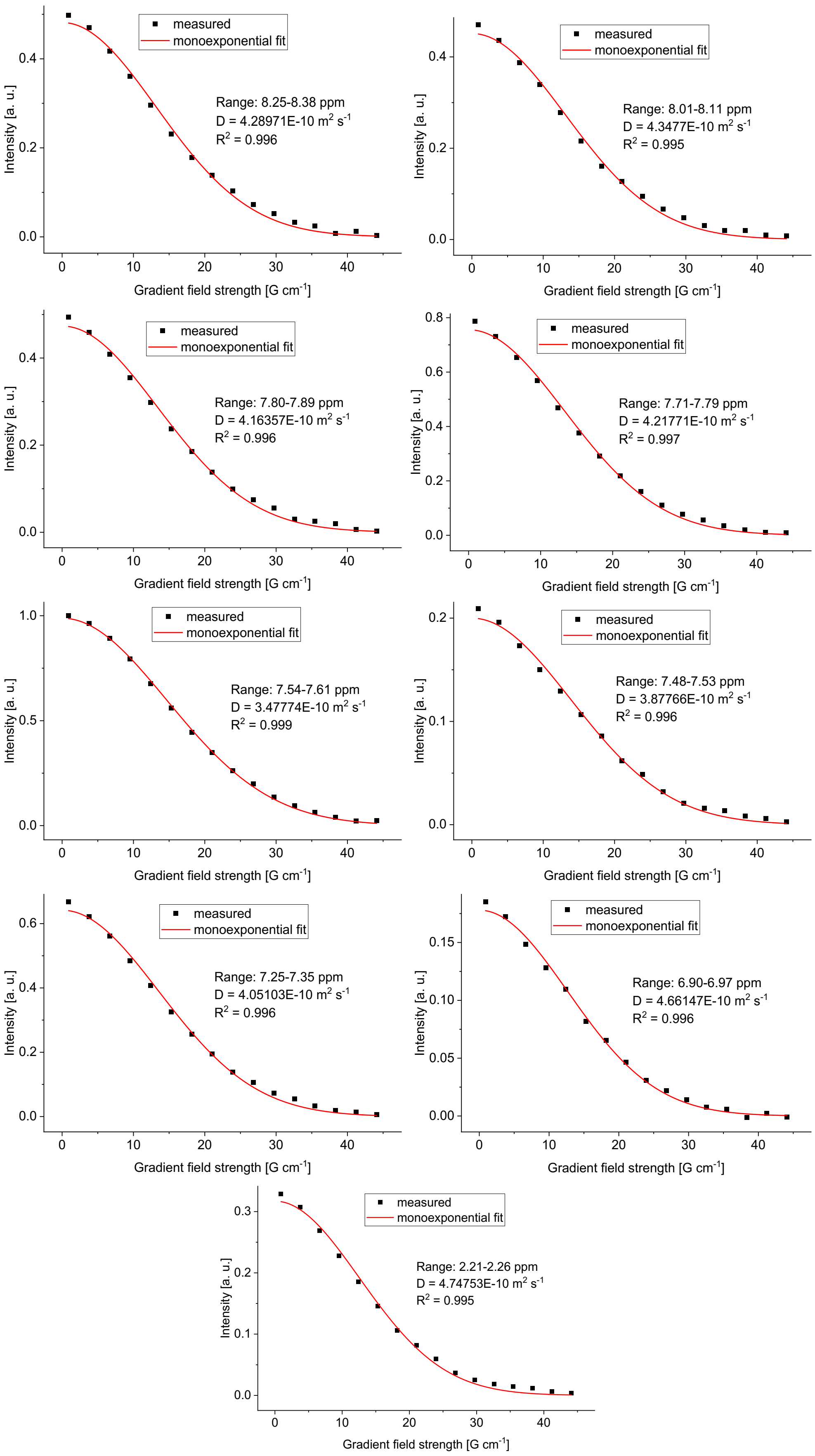

Figure S63. Monoexponential fit curves for the amplitude decay for the proton signals obtained during ^{1}H DOSY NMR measurements of *pic*[**Ru₅C₄**]*pic* (led, 600 MHz, CD_2Cl_2/TFE-d_3 9:1, 295.6 K, ascorbic acid, TMSS).

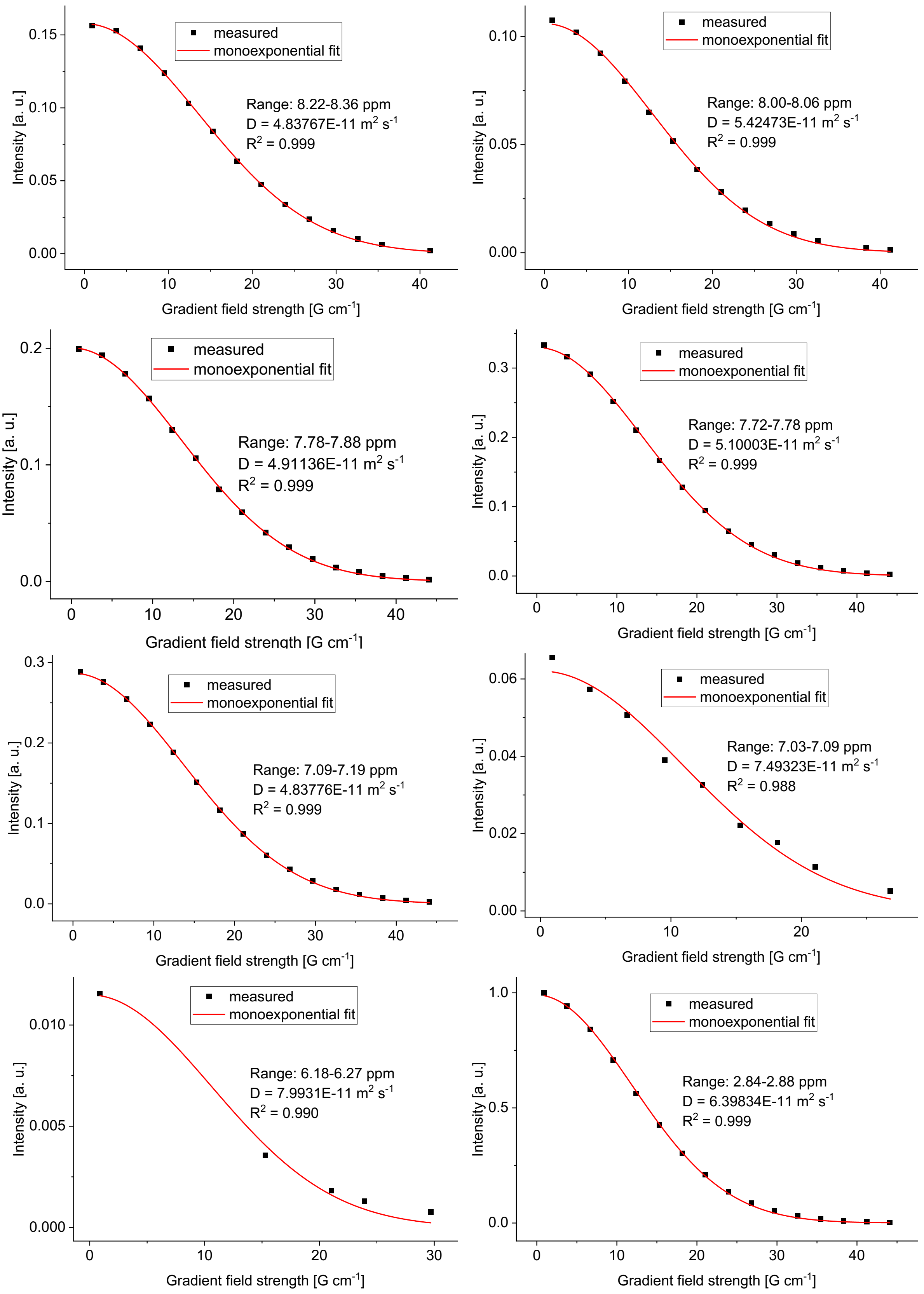

Figure S64. Monoexponential fit curves for the amplitude decay for the proton signals obtained during ^{1}H DOSY NMR measurements of *dmap*[**Ru$_4$*B*$_3$**]*dmap* (led, 600 MHz, CD_2Cl_2/TFE-d_3 9:1, 295.6 K, ascorbic acid, TMSS).

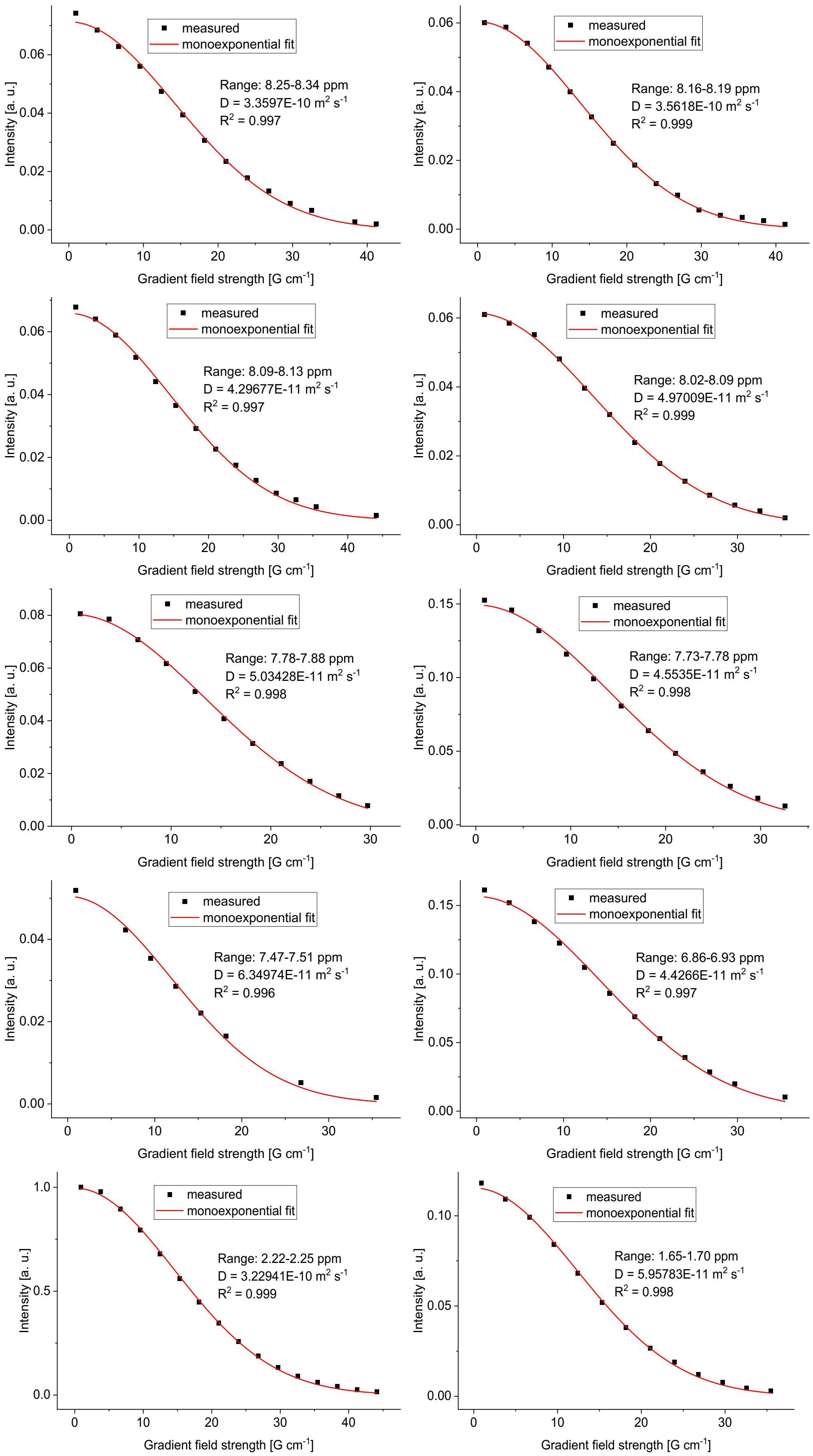

Figure S65. Monoexponential fit curves for the amplitude decay for the proton signals obtained during ^{1}H DOSY NMR measurements of *pic*[**Ru$_4$*C*^{Me}$_3$**]*pic* (dste, 600 MHz, CD_2Cl_2/TFE-*d*$_3$ 9:1, 295.6 K, ascorbic acid, TMSS).

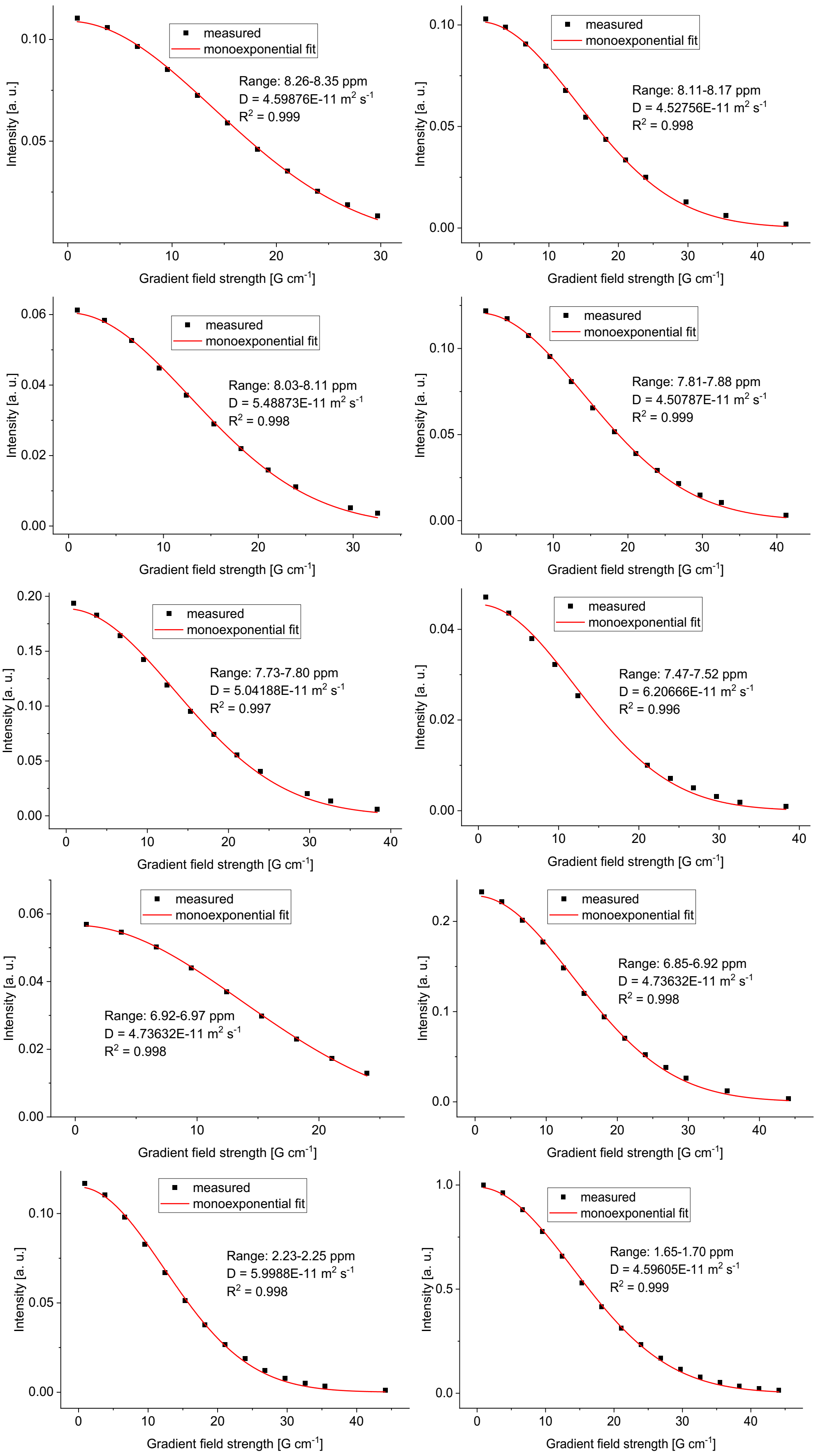

Figure S66. Monoexponential fit curves for the amplitude decay for the proton signals obtained during ^{1}H DOSY NMR measurements of *pic*[**Ru$_5$*C*^{Me}$_4$**]*pic* (led, 600 MHz, CD_2Cl_2/TFE-*d*$_3$ 9:1, 295.6 K, ascorbic acid, TMSS).

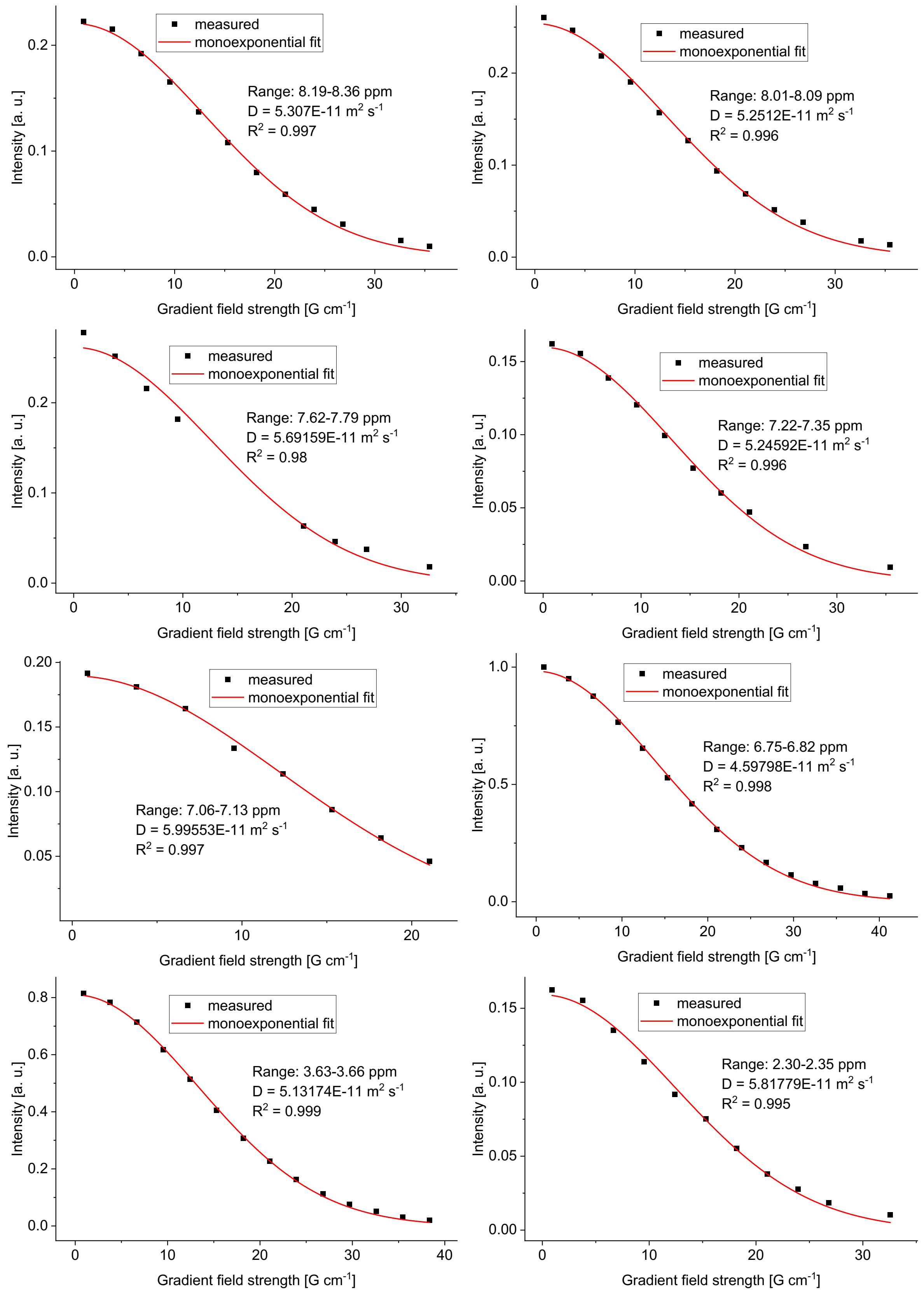

Figure S67. Monoexponential fit curves for the amplitude decay for the proton signals obtained during ^{1}H DOSY NMR measurements of *pic*[$\mathbf{Ru_4C^{\mathit{OMe}}}_3$]*pic* (led, 600 MHz, CD_2Cl_2/TFE-d_3 9:1, 295.6 K, ascorbic acid, TMSS).

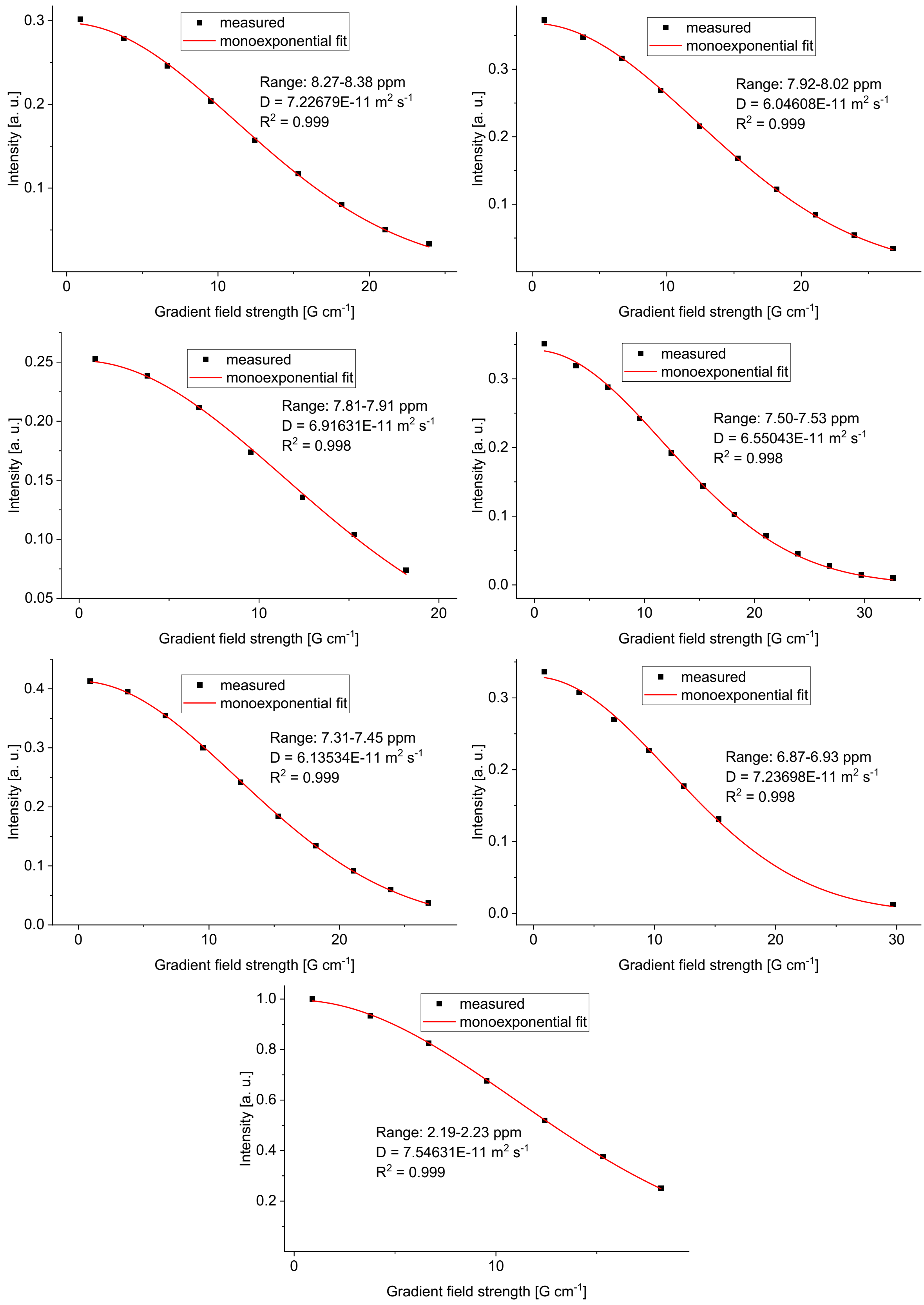

Figure S68. Monoexponential fit curves for the amplitude decay for the proton signals obtained during ^{1}H DOSY NMR measurements of *pic*[**Ru$_n$*A*$_{n-1}$**]*pic* (dste, 600 MHz, CD_2Cl_2/TFE-d_3 9:1, 295.6 K, ascorbic acid, TMSS).

6 Molecular Modelling

Molecular modelling was performed with the program *Materials Studio*, Version 17.1, BIOVIA. [S19] Geometry Optimization with the Universal Force-Field (UFF) was done with the *forcite* task.

Images of the geometry-optimized molecular structures were produced with Mercury, Version 2021.2.0, Build 327562. [S20]

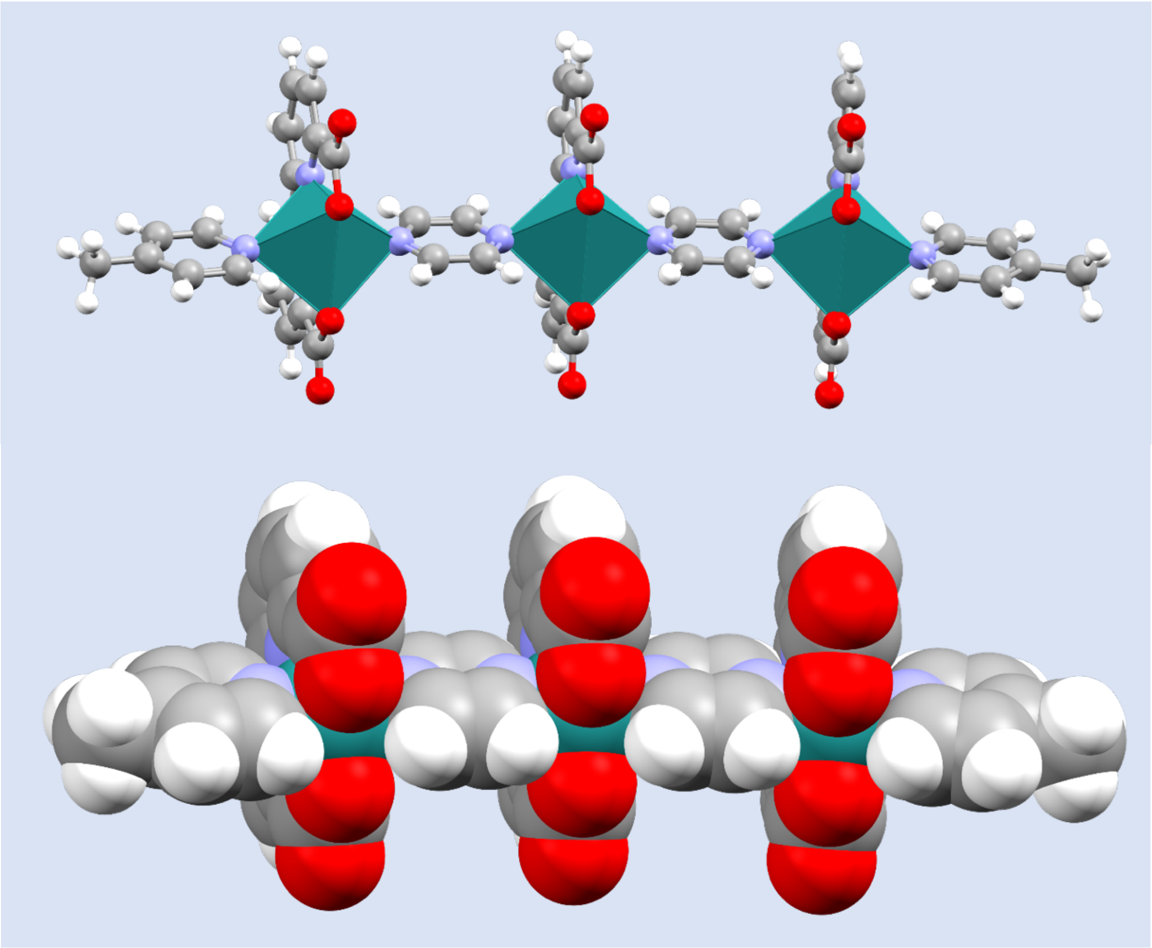

Figure S69. Ball-and-stick model with metal coordination polyhedra (top) and space-filling model (bottom) of hypothetical *pic*[**Ru$_3$*A*$_2$**]*pic* (C grey, H white, O red, N blue, Ru turquoise).

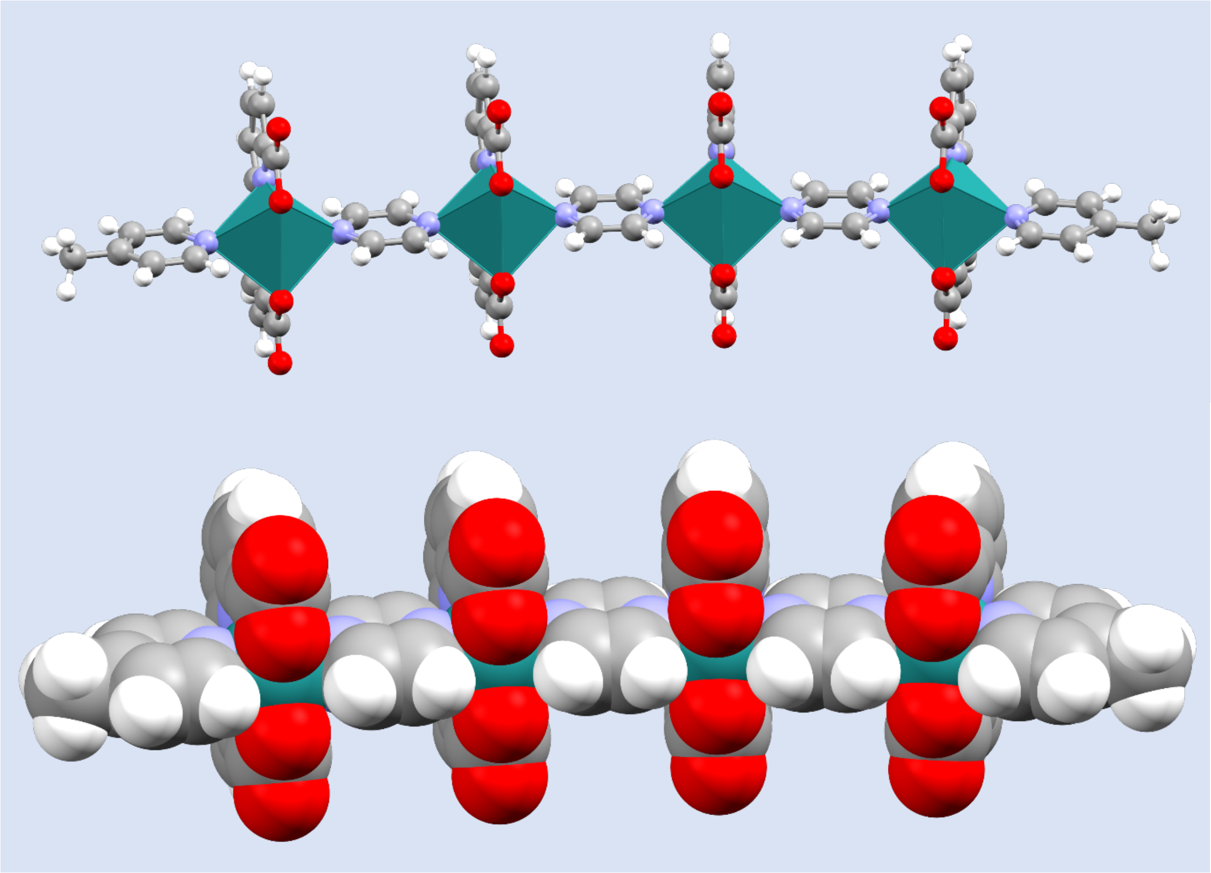

Figure S70. Ball-and-stick model with metal coordination polyhedra (top) and space-filling model (bottom) of hypothetical *pic*[$\mathbf{Ru_4}\boldsymbol{A_3}$]*pic* (C grey, H white, O red, N blue, Ru turquoise).

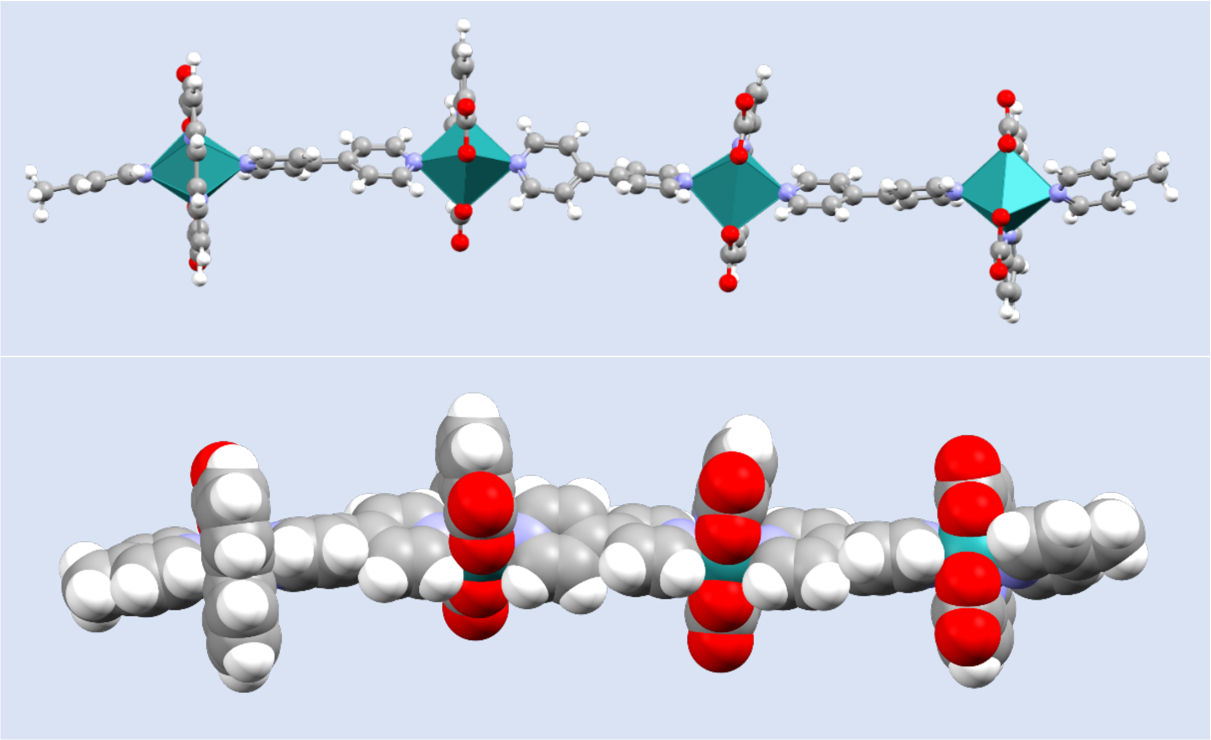

Figure S71. Ball-and-stick model with metal coordination polyhedra (top) and space-filling model (bottom) of *pic*[$\mathbf{Ru_4}\boldsymbol{B_3}$]*pic* (C grey, H white, O red, N blue, Ru turquoise).

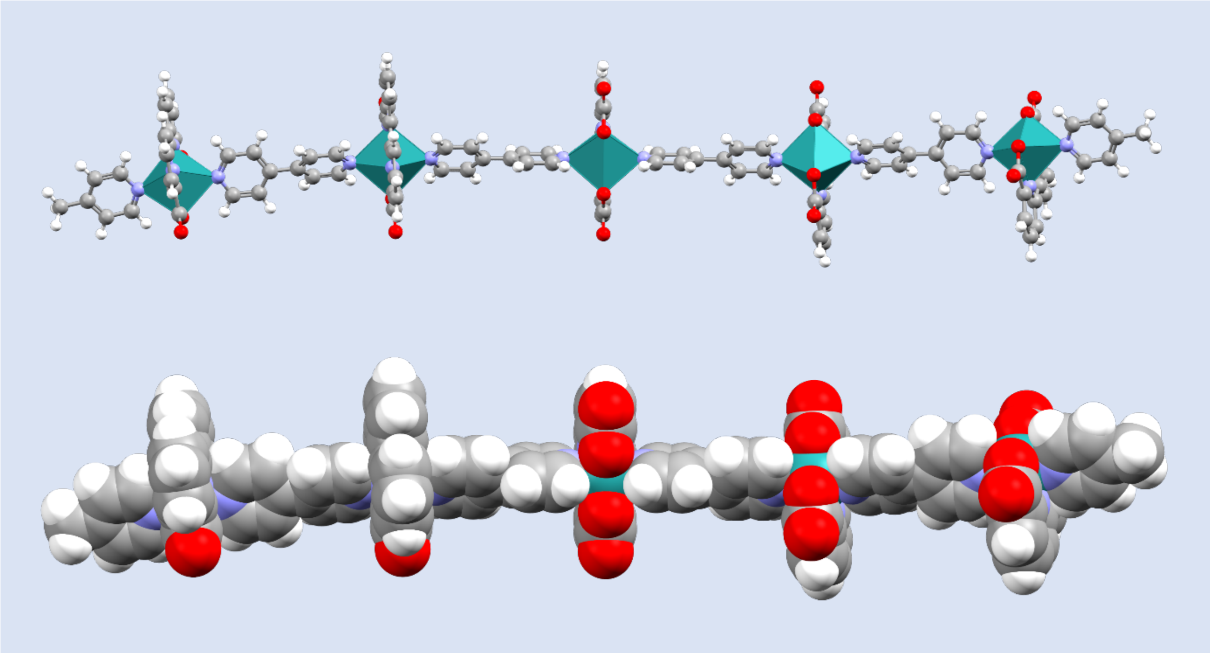

Figure S72. Ball-and-stick model with metal coordination polyhedra (top) and space-filling model (bottom) of *pic*[**Ru$_5$*B*$_4$**]*pic* (C grey, H white, O red, N blue, Ru turquoise).

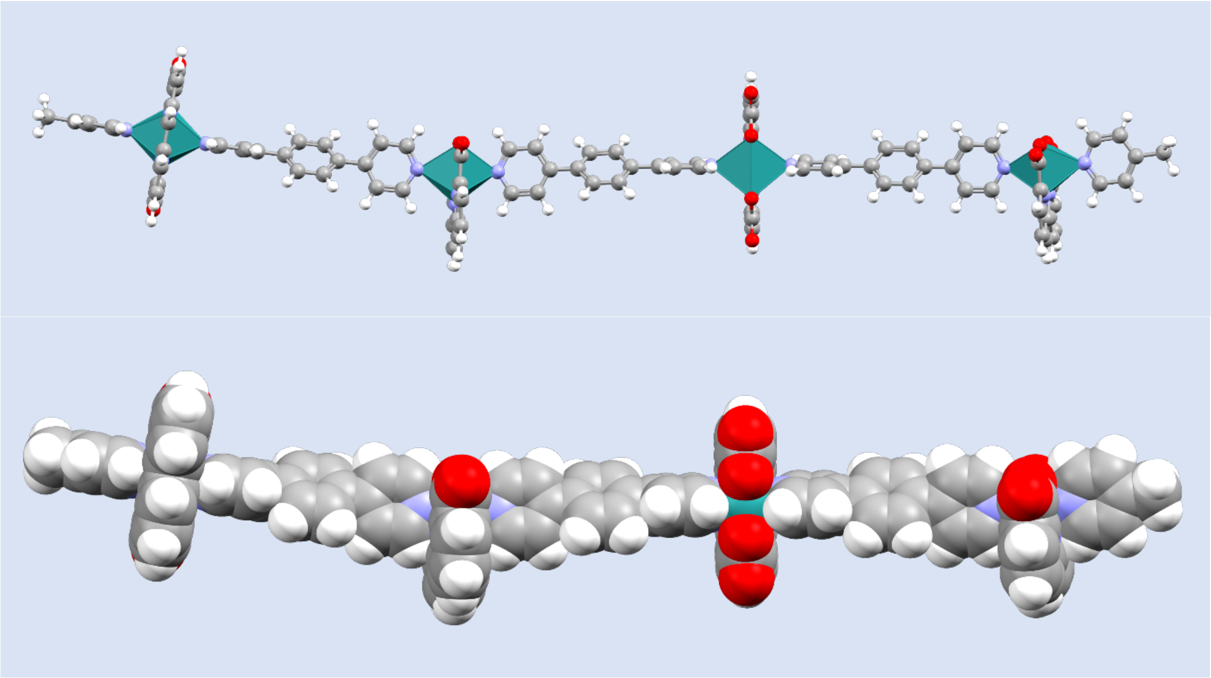

Figure S73. Ball-and-stick model with metal coordination polyhedra (top) and space-filling model (bottom) of *pic*[**Ru$_4$*C*$_3$**]*pic* (C grey, H white, O red, N blue, Ru turquoise).

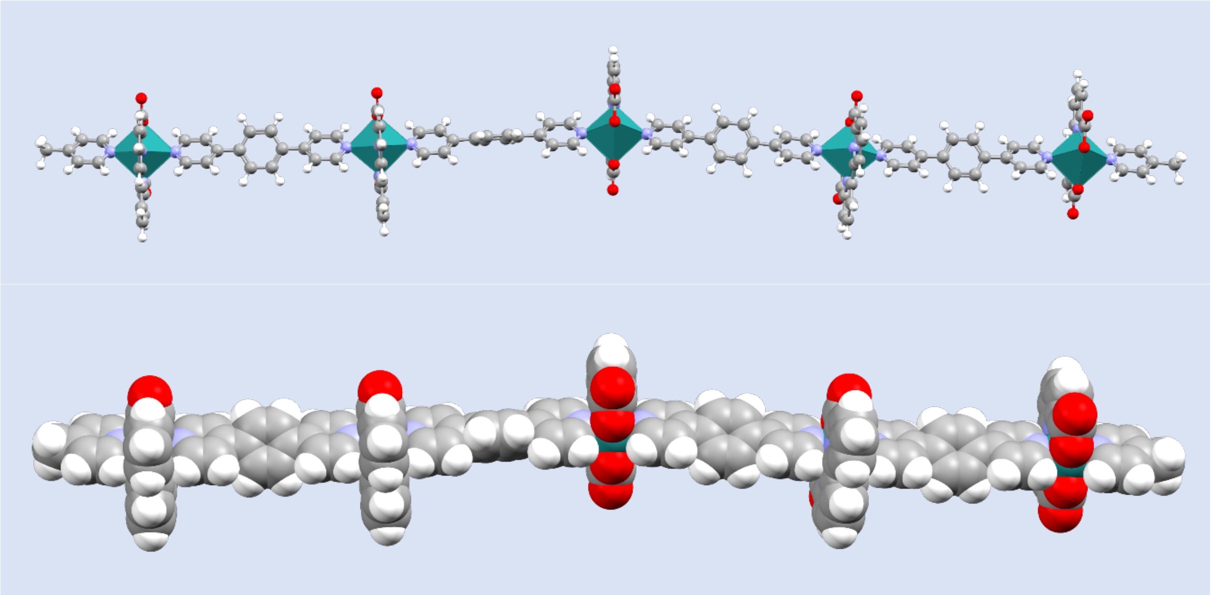

Figure S74. Ball-and-stick model with metal coordination polyhedra (top) and space-filling model (bottom) of *pic*[**Ru$_5$*C*$_4$**]*pic* (C grey, H white, O red, N blue, Ru turquoise).

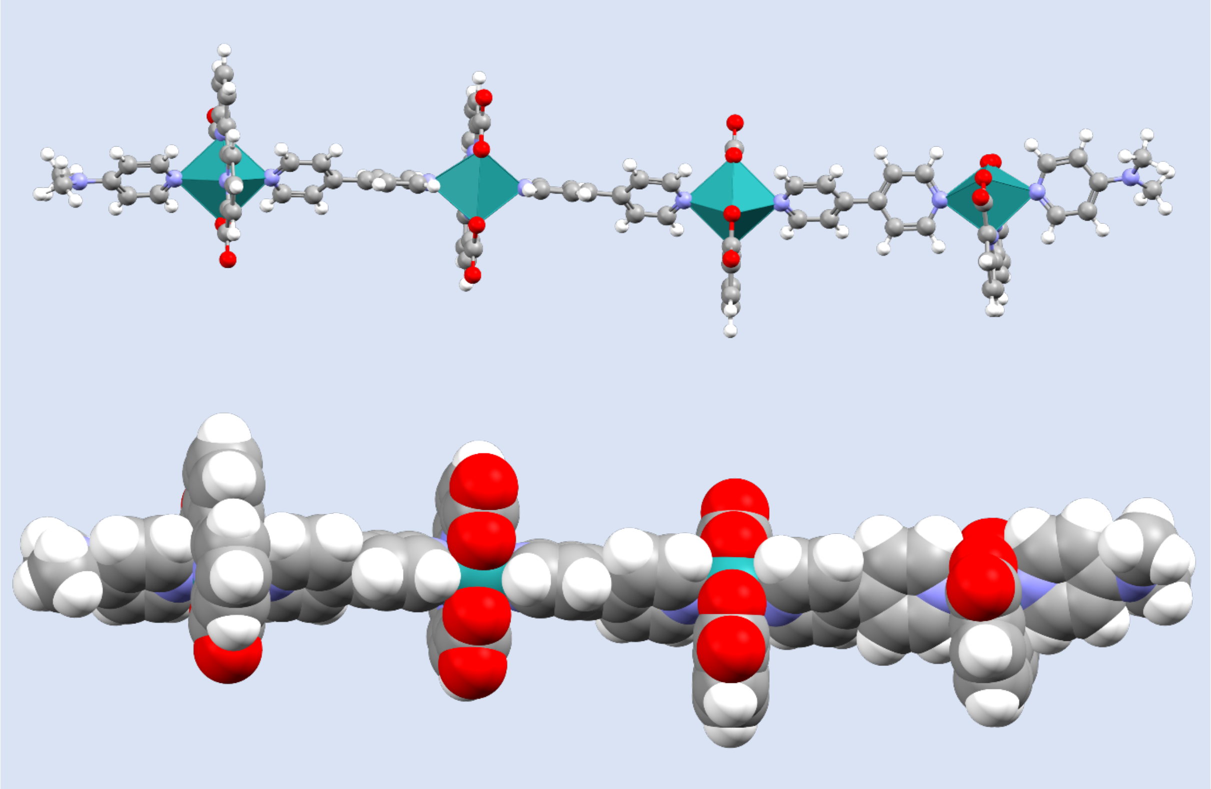

Figure S75. Ball-and-stick model with metal coordination polyhedra (top) and space-filling model (bottom) of *dmap*[**Ru$_4$*B*$_3$**]*dmap* (C grey, H white, O red, N blue, Ru turquoise).

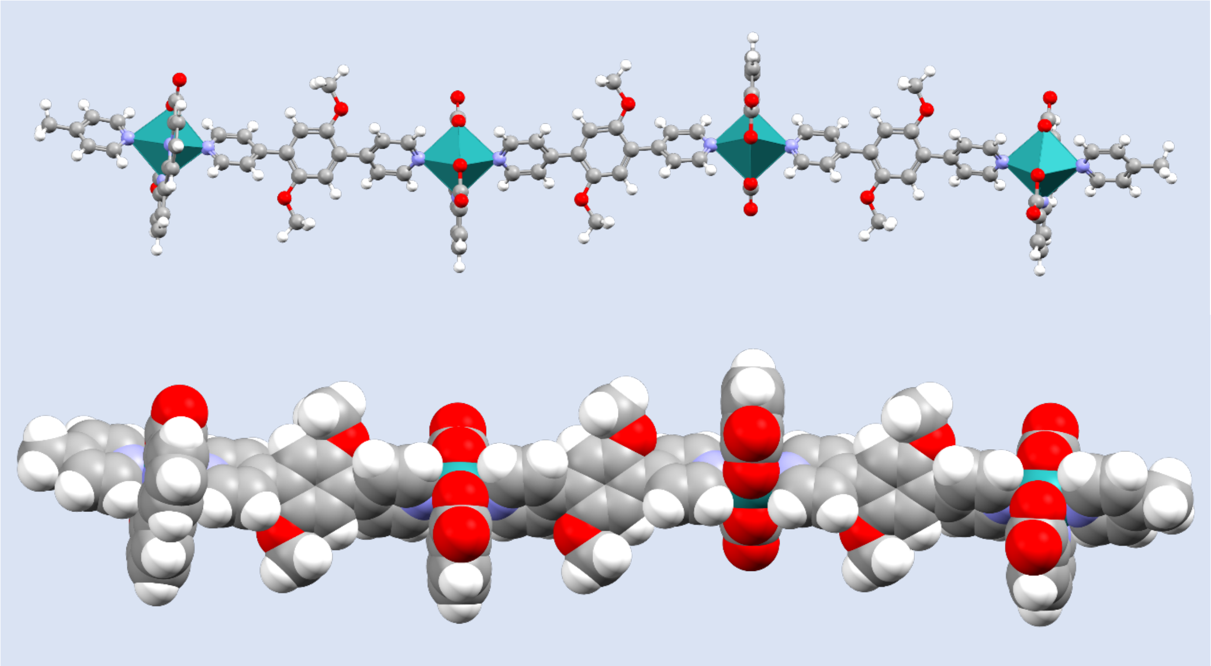

Figure S76. Ball-and-stick model with metal coordination polyhedra (top) and space-filling model (bottom) of *pic*[**Ru₄*C*^{OMe}₃**]*pic* (C grey, H white, O red, N blue, Ru turquoise).

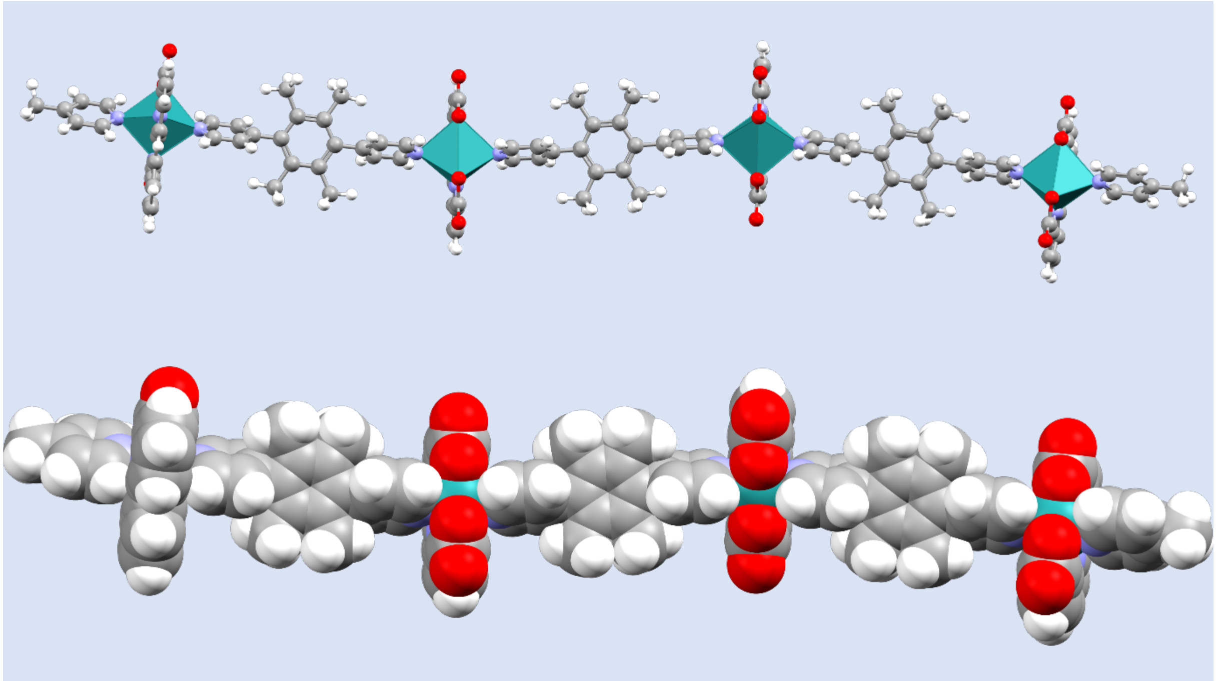

Figure S77. Ball-and-stick model with metal coordination polyhedra (top) and space-filling model (bottom) of *pic*[**Ru₄*C*^{Me}₃**]*pic* (C grey, H white, O red, N blue, Ru turquoise).

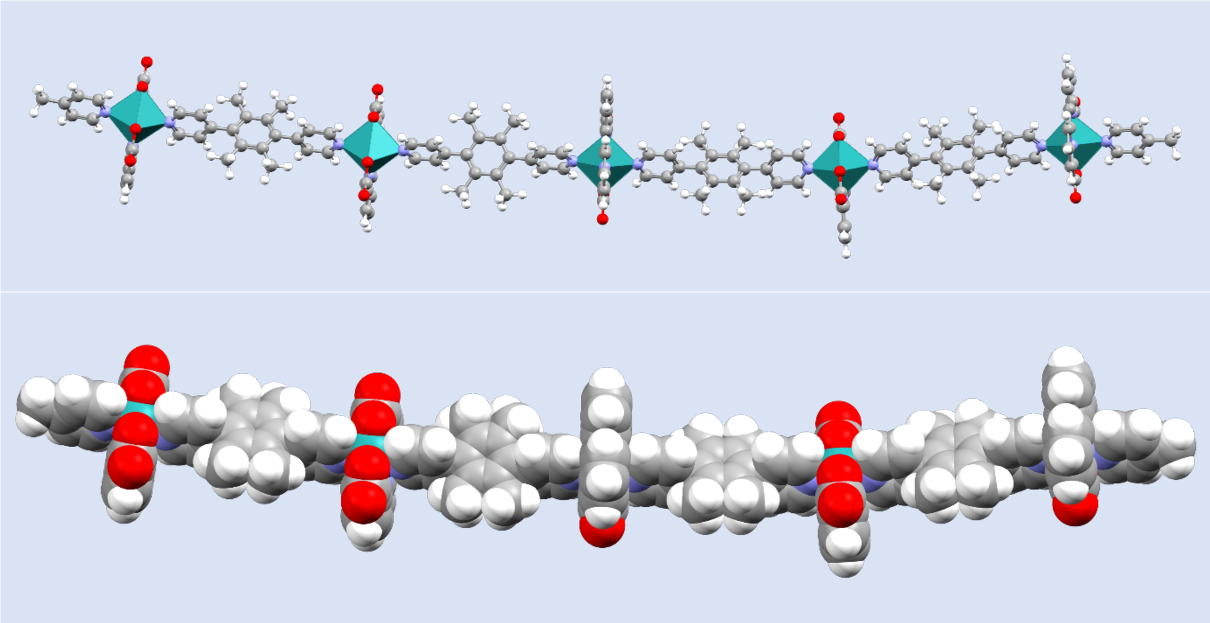

Figure S78. Ball-and-stick model with metal coordination polyhedra (top) and space-filling model (bottom) of *pic*[**Ru₅*C*^*Me*^₄**]*pic* (C grey, H white, O red, N blue, Ru turquoise).

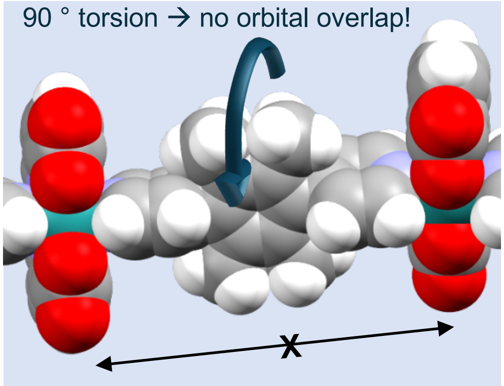

Figure S79. Section of a molecular model of *pic*[**Ru₄*C*^*Me*^₃**]*pic* that highlights the orthogonal torsion angle in linker **C^Me^**.

7 References

[S1] G. R. Fulmer, A. J. M. Miller, N. H. Sherden, H. E. Gottlieb, A. Nudelman, B. M. Stoltz, J. E. Bercaw, K. I. Goldberg, *Organometallics* **2010**, *29*, 2176–2179.

[S2] J. Novotný, M. Sojka, S. Komorovsky, M. Nečas, R. Marek, *J. Am. Chem. Soc.* **2016**, *138*, 8432–8445.

[S3] *TopSpin 4.0*, Bruker, www.bruker.com.

[S4] F. Li, B. Zhang, X. Li, Y. Jiang, L. Chen, Y. Li, L. Sun, *Angew. Chem. Int. Ed.* **2011**, *50*, 12276–12279.

[S5] C. L. Donnici, D. H. Máximo Filho, L. L. C. Moreira, G. T. d. Reis, E. S. Cordeiro, I. M. F. d. Oliveira, S. Carvalho, E. B. Paniago, *J. Braz. Chem. Soc.* **1998**, *9*, 455–460.

[S6] E. Dulière, M. Devillers, J. Marchand-Brynaert, *Organometallics* **2003**, *22*, 804–811.

[S7] Y. Jiang, F. Li, F. Huang, B. Zhang, L. Sun, *Chinese J. Catal.* **2013**, *34*, 1489–1495.

[S8] K. Biradha, M. Fujita, *J. Chem. Soc., Dalton Trans.* **2000**, 3805–3810.

[S9] K.-i. Yamashita, K.-i. Sato, M. Kawano, M. Fujita, *New J. Chem.* **2009**, *33*, 264–270.

[S10] O. Karagiaridi, W. Bury, E. Tylianakis, A. A. Sarjeant, J. T. Hupp, O. K. Farha, *Chem. Mater.* **2013**, *25*, 3499–3503.

[S11] B. D. Sherman, Y. Xie, M. V. Sheridan, D. Wang, D. W. Shaffer, T. J. Meyer, J. J. Concepcion, *ACS Energy Lett.* **2017**, *2*, 124–128.

[S12] A. Vidal, F. Adamo, E. Iengo, E. Alessio, *Inorg. Chim. Acta* **2021**, *516*, 120143.

[S13] D. C. Aluthge, J. M. Ahn, P. Mehrkhodavandi, *Chem. Sci.* **2015**, *6*, 5284–5292.

[S14] G. Li, J. X. Tang, *Phys. Rev. E* **2004**, *69*, 061921.

[S15] M. M. Tirado, C. L. Martínez, J. G. d. l. Torre, *J. Chem. Phys.* **1984**, *81*, 2047–2052.

[S16] A. V. Anantaraman, *Can. J. Chem.* **1986**, *64*, 46–50.

[S17] M. Holz, X. a. Mao, D. Seiferling, A. Sacco, *J. Chem. Phys.* **1996**, *104*, 669–679.

[S18] a) *Handbook of chemistry and physics: a ready-reference book of chemical and physical data*, 76th ed., CRC Press, Cleveland, **1996**; b) K.-S. Kim, H. Lee, *J. Chem. Eng. Data* **2002**, *47*, 216–218.

[S19] BIOVIA, Dassault Systèmes, BIOVIA Materials Studio 2017, Release 17.1.0.48, San Diego: Dassault Systèmes, 2017.

[S20] C. F. Macrae, I. Sovago, S. J. Cottrell, P. T. A. Galek, P. McCabe, E. Pidcock, M. Platings, G. P. Shields, J. S. Stevens, M. Towler, P. A. Wood, *J. Appl. Crystallogr.* **2020**, *53*, 226–235.
